# Indium‐Catalysed Transfer Hydrogenation for the Reductive Cyclisation of 2‐Alkynyl Enones towards Trisubstituted Furans

**DOI:** 10.1002/anie.202109266

**Published:** 2021-10-01

**Authors:** Luomo Li, Sascha Kail, Sebastian M. Weber, Gerhard Hilt

**Affiliations:** ^1^ Institut für Chemie Carl von Ossietzky Universität Oldenburg Carl-von-Ossietzky-Strasse 9–11 26111 Oldenburg Germany

**Keywords:** 1,4-cyclohexadienes, cyclisation, deuterium labelling, furans, indium

## Abstract

Indium tribromide catalysed the transfer hydrogenation from dihydroaromatic compounds, such as the commercially available γ‐terpinene, to enones, which resulted in the cyclisation to trisubstituted furan derivatives. The reaction was initiated by a Michael addition of a hydride nucleophile to the enone subunit followed by a Lewis‐acid‐assisted cyclisation and the formation of a furan–indium intermediate and a Wheland intermediate derived from the dihydroaromatic starting material. The product was formed by protonation from the Wheland complex and replaced the indium tribromide substituent. In addition, a site‐specific deuterium labelling of the dihydroaromatic HD surrogates resulted in site specific labelling of the products and gave useful insights into the reaction mechanism by H–D scrambling.

## Graphical abstract

InBr_3_ is used as functional‐group‐tolerant catalyst for the transfer hydrogenation from γ‐terpinene as H_2_ surrogate to initiate the cyclisation of 2‐alkynyl‐substituted enones to trisubstituted furans in good yields. *Design of Experiments* was used to optimise the reaction conditions with a few experiments to almost quantitative yields and deuterium‐labelling experiments gave valuable mechanistic insights.

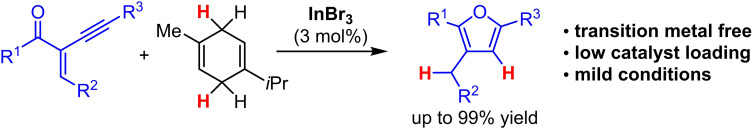

Multiple substituted furans play an important role in organic chemistry, not only as key structural motives in natural products (e.g., crassifogenin A^[1a]^ and plakorsin D,^[1b]^ Figure 1) but also in materials and pharmaceuticals. The synthesis of furans has a long tradition in organic synthesis, and recent developments have been highlighted in several reviews.^[2]^

**Figure 1:**
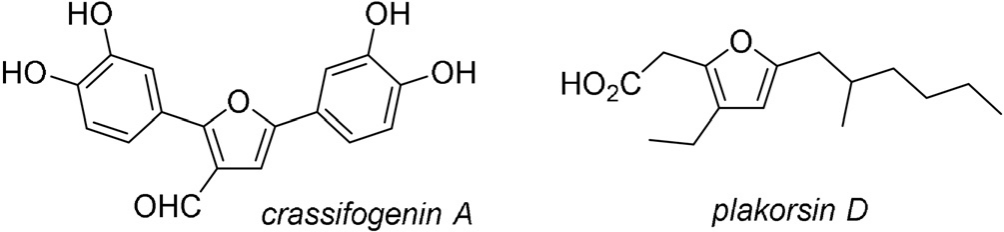
Highly functionalised furan derivative pharmaceuticals and natural products.

Among the numerous synthetic approaches towards furans, the cyclisation of 2‐alkynyl‐substituted 1,3‐conjugated enones **1** with alcohols was first reported by Larock^[3]^ in 2004 (Scheme 1) utilising catalytic amounts of gold(III) and various nucleophiles for the synthesis of trisubstituted furans. Further developments by Zhang, Liu, and several other groups (Scheme 1) utilising transition metal catalysts, such as gold,[^4a^, ^4b^, ^4c^, ^4d^, ^4e^, ^4f^, ^4g^, ^4h^, ^4i^, ^4j^, ^4k^, ^4l^, ^4m^, ^4n^] palladium,[^4o^, ^4p^, ^4q^, ^4r^] rhodium,^[4s]^ copper,[^4t^, ^4u^] and silver,[^4v^, ^4w^, ^4x^] were reported over the last two decades. In these transition‐metal‐catalysed cyclisation reactions of alkynyl enones of type **1**, a large number of different types of nucleophiles were reported for the synthesis of highly substituted and functionalised furans and annulated bicyclic systems. However, the use of a hydride source as nucleophile seems to be missing, probably because the transition metal catalysts are incompatible with hydride donors. Nevertheless, these applications utilised transition metal catalysts for the synthesis of highly substituted/functionalised furans via a π‐Lewis acid activation of the alkyne moiety. In an outstanding report by Selander,^[5]^ InBr_3_ catalysed the synthesis of furan derivatives starting from **1** in an annulation process with in situ generated enamines (Scheme 1).

**Scheme 1:**
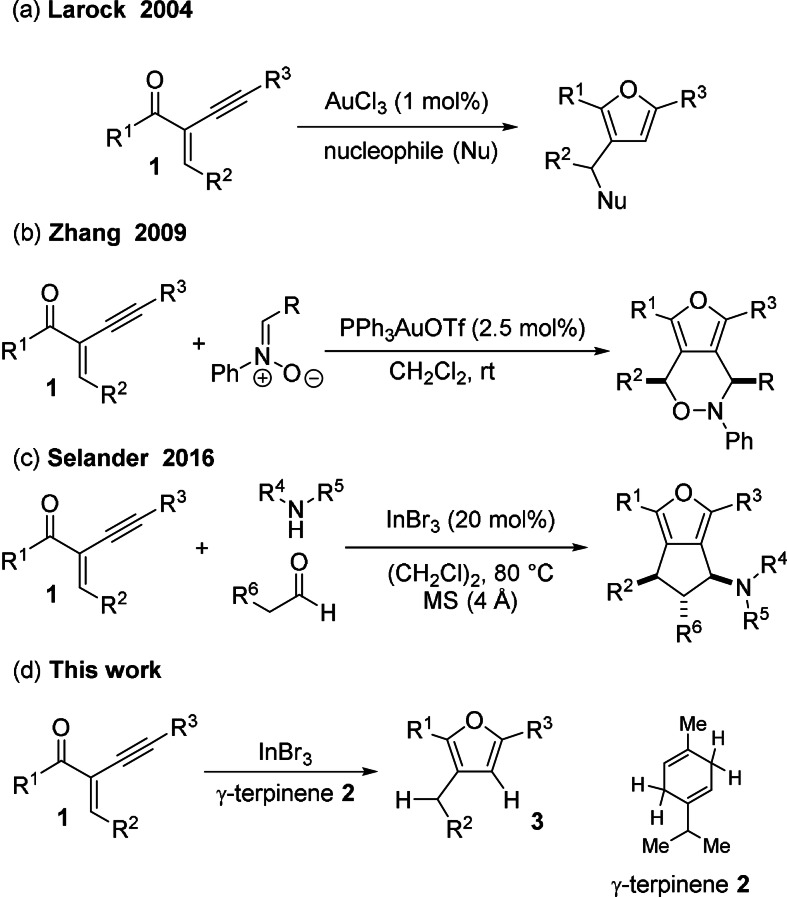
Previous work concerning the cyclisation of 2‐alkynyl‐substituted enones with nucleophiles.

The Oestreich group pioneered the transfer hydrogenation of alkenes and imines by using dihydroaromatic compounds as the H_2_ surrogate catalysed by B(C_6_F_5_)_3_ or strong Brønsted acids^[6]^ and applied regiospecific deuterated dihydroaromatic cyclohexadiene as a HD surrogate.^[6g]^ In 2020 our group reported the regiodivergent hydrodeuterogenation and the deuterohydrogenation utilising two specific deuterium‐labelled dihydroaromatic compounds.^[7]^ Therefore, we became interested in expanding the cyclisation of enone **1** with a hydride nucleophile from a dihydroaromatic compound. Herein, an InBr_3_‐catalysed cyclisation of alkynyl enones **1** with the cost‐efficient and commercially available dihydroaromatic compound γ‐terpinene (<100 € kg^−1^) as H_2_ surrogate towards furans under C−O bond formation is described.

For the optimisation of the cyclisation reaction, we focused on the following parameters:

- the catalyst loading (3–10 mol %, continuous)
- the reaction temperature (0–50 °C, continuous)
- the reaction time (1–15 h, continuous)
- the substrate concentration (0.2–2 m, continuous)
- the reducing agent loading (0.95–1.5 equiv., continuous)
- the type of the reductant (1,4‐cyclohexadiene or γ‐terpinene, categorical)

For the efficient optimisation of all these variables and to reduce the number of needed experiments, the *Design of Experiments* (DoE) approach,^[8]^ with 2‐benzylidene‐1,4‐diphenylbut‐3‐yn‐1‐one **1 a** as test substrate (Scheme 2), was applied. All of the parameters were optimised in only 16 experiments (Figure 2; for further details, see Supporting Information).

**Figure 2:**
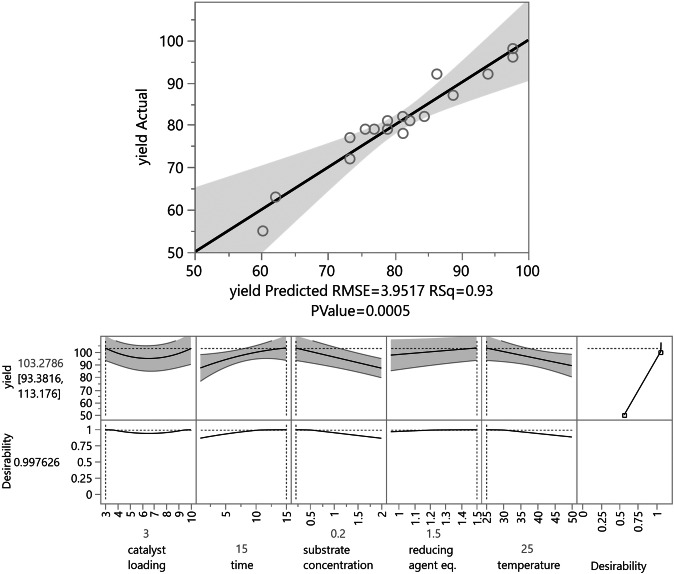
Reaction optimisation for the cyclisation of alkenyl enone **1 a** with H_2_ surrogates. The predicted yields are plotted vs. the measured yields. Total number of reactions: 19; 16 for the model, and three duplicates for the lack of fit. The yields were determined by GC/FID analysis using mesitylene as internal standard.

**Scheme 2:**
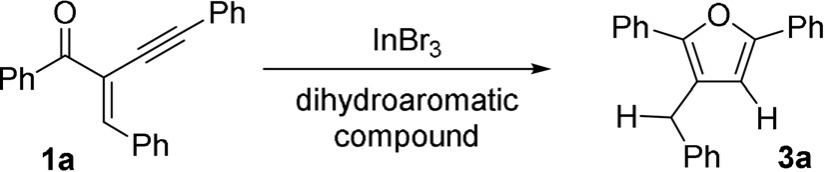
Test reaction for the optimisation for the reaction parameters by DoE.

The resulting model identified eight relevant factors with an *R* ^2^=0.93 (see Supporting Information). The most relevant factors were the substrate concentration and the reaction time (*p*‐values <0.01). The quadratic temperature and the cross‐interaction between time and temperature (*p*‐values=0.3) also affected the design.

Surprisingly, the model showed that the catalyst loading (*p*‐values=0.8) had a marginal impact while a low concentration of the substrate and a low reaction temperature improved the yield and 1.5 equiv. of reductant were sufficient for the reaction. According to the calculated model, the optimal reaction conditions (Scheme 3) were applied for the synthesis of **3 a**, which was isolated in 98 % yield (predicted yield around 100 %), and a variety of alkynyl enones of type **1** were transformed in the InBr_3_‐catalysed cyclisation towards the furans of type **3** (Scheme 3). The results of the InBr_3_‐catalysed transfer‐hydrogenation‐induced cyclisation reactions are summarised in Table 1.

**Scheme 3:**
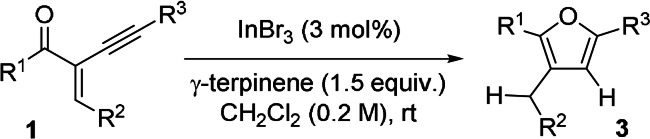
InBr_3_‐catalysed cyclisation of 2‐alkynyl‐substituted enones **1**.

**Table 1:**
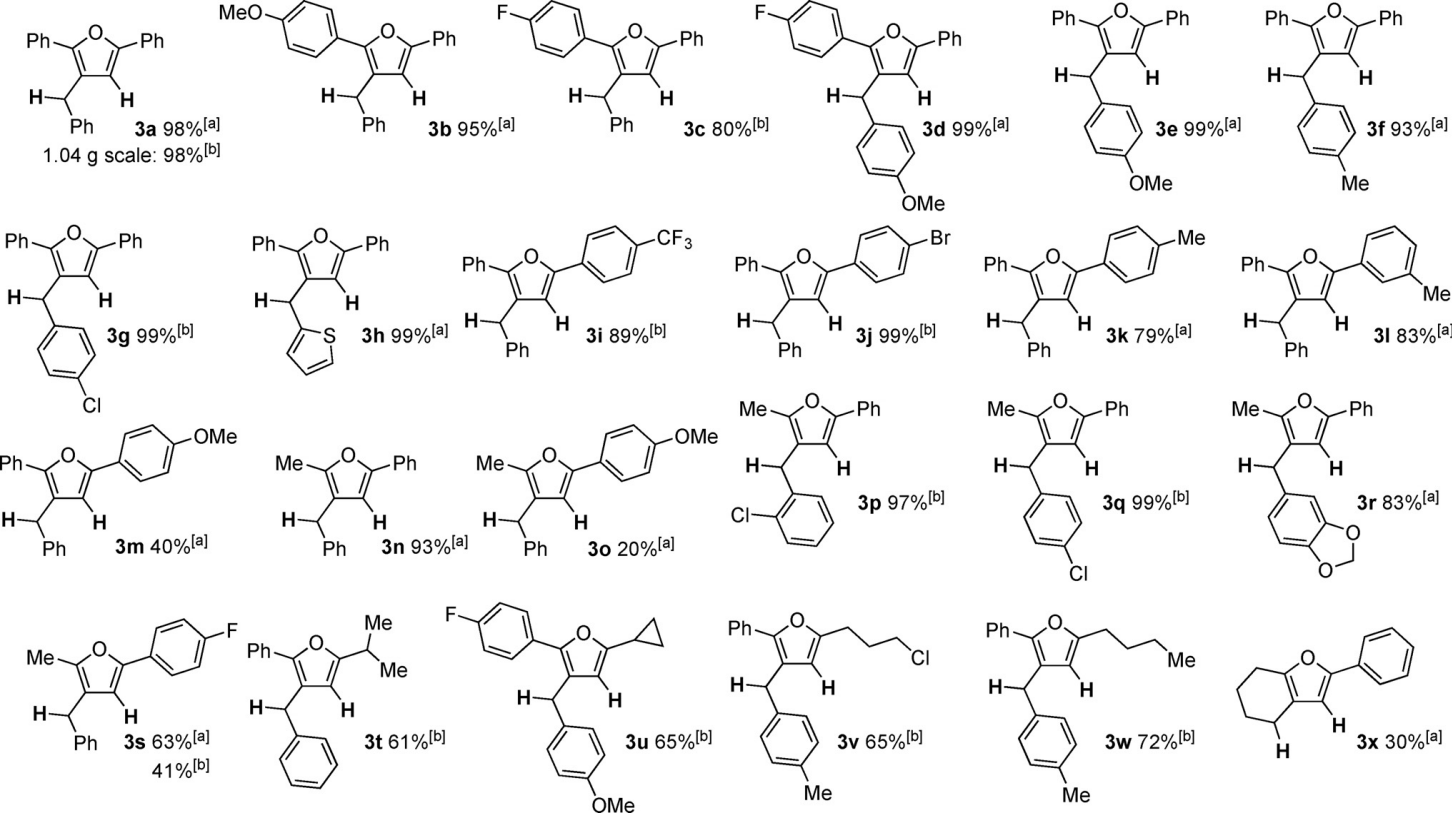
Scope of the InBr_3_‐catalysed transfer hydrogenation and cyclisation of 2‐alkynyl‐substituted enones **1**. 
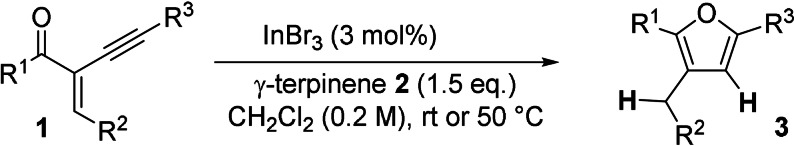

General reaction conditions: alkynyl enones **1** (1.00 mmol, 1.0 equiv.), g‐terpinene **2** (1.50 mmol, 1.5 equiv.), InBr_3_ (0.03 mmol, 3 mol %), and CH_2_Cl_2_ (5 mL); 15–24 h and for the products **3 h** and **3 r** up to 72 h. [a] The reaction was performed at rt. [b] The reaction was performed at 50 °C.

Fortunately, the indium catalyst system tolerated a broad range of functional groups. Enones comprising electron‐rich as well as electron‐deficient aromatic substituents on the acyl moiety (R^1^) led to products **3 a**–**3 d** in good to excellent yields. Moreover, the substituent on the acyl part (R^1^) can be an aliphatic group as well (**3 n**–**3 s**), which had little impact on the yield. The alkenyl moiety (R^2^) with both electron‐rich and electron‐deficient aryl groups was compatible (**3 e**–**3 h**), including the 2‐thiophenyl group. Aryl substituents on the alkynyl moiety (R^3^, **3 i**–**3 m**) were also tolerated while electron‐deficient aryl groups, such as 4‐trifluoromethyl‐substituted substrate **1 i** and the 4‐bromo‐substituted aryl substrate **1 j**, led to lower reactivities at ambient temperature, but the corresponding products (**3 i** and **3 j**) were formed with good and almost quantitative yields at 50 °C. Also, the benzo[*d*][1,3]dioxole derivative **1 r** led to the formation of product **3 r** in a good yield (83 %), while the reaction required a long time (more than 3 days) to reach completion, probably by lowering the catalyst activity upon weak coordination. Also, moderate to good yields were obtained for the products (**3 t**–**3 w**) comprising alkyl groups on the alkynyl moiety R^3^, including a cyclopropyl group (**3 u**) and a ω‐chloroalkyl substituent in product **3 v**. Unfortunately, the investigation of substrates with an aliphatic side‐chain as substituent R^2^ could not be realised. The route for the synthesis for this type of starting material with aliphatic substituents as R^2^ led either to decomposition, side‐reactions towards unidentified products, or only trace amounts of the desired enone derivatives. However, one exception is the cyclohexanone derivative **1 x**, which could be reacted successfully to afford **3 x** in a moderate yield thus indicating that also alkyl chains are tolerated as R^2^, but other synthetic routes must be used to access such starting materials. On the basis of the results described by Oestreich and us, substrates bearing strongly Lewis‐basic groups, such as a 4‐nitrophenyl or a pyridyl substituent, are poor substrates for the transfer hydrogenation from diaromatic compounds.^[6g]^ In our previous work (unpublished), the CN group decreased the reactivity of indium catalyst for the transfer hydrogenation and lead to low or no conversion. Notably, this indium‐catalysed cyclisation of alkynyl enones can be easily scaled up. A gram‐scale reaction of alkynyl enone **1 a** was examined at 50 °C, providing 1.05 g (3.39 mmol) of **3 a** in 98 % yield.

To gain a better understanding of the mechanism of this alkynyl enone cyclisation, we conducted regiodiverse deuterium‐labelling experiments utilising the two regioselectively substituted dihydroaromatic compounds **4** (99 % D incorporation) and **5** (96 % D incorporation) developed by our group in 2020 as HD surrogates.[^7^, ^9^] The reaction of **1 f** with surrogate **4** providing a deuteride (D^−^) and a proton (H^+^) in the presence of InBr_3_ at 50 °C afforded the furan **6** in 85 % yield and 99 % deuterium incorporation into the methylene group (Scheme 4).

**Scheme 4:**
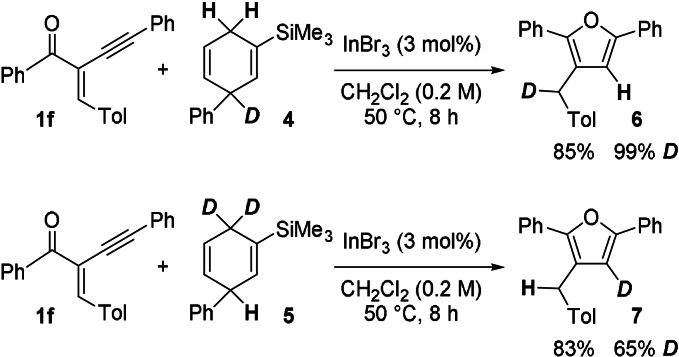
Hydrodeuterogenation and deuterohydrogenation of enone **1 f**.

The same reaction with the surrogate **5** providing a deuterium cation (D^+^) a hydride (H^−^) afforded the furan **7** with 83 % yield and 65 % deuterium incorporation into the 3‐position of the furan ring was detected. The significant loss of deuterium labelling for the HD surrogate **5** was surprising. Neither deuterium labelling at other positions of product **7** nor additional deuterium incorporation in the oxidised HD surrogate (4‐deutero[1,1′‐biphenyl]‐3‐yl)trimethylsilane) were detectable by GCMS analysis.

Nevertheless, for the mechanistic considerations of this InBr_3_‐catalysed reaction the considerable loss of deuterium labelling might be informative. For the gold‐catalysed cyclisation of alkynyl enones (see Scheme 1), a plausible mechanism was proposed by Larock.^[3]^ The cyclisation was initiated by a AuCl_3_‐catalysed π‐activation of the alkyne. However, compared to the gold catalyst, InBr_3_ is believed to be more oxophilic. This led to a mechanistic proposal shown in Scheme 5.

**Scheme 5:**
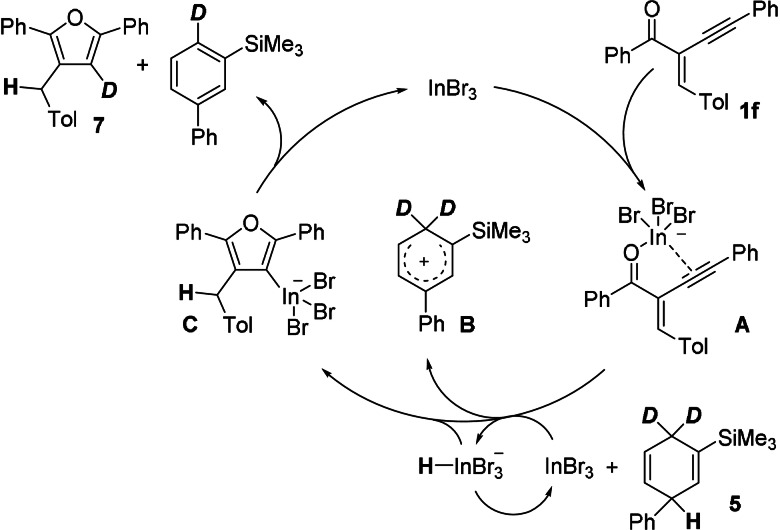
Possible mechanism for the InBr_3_‐catalysed cyclisation of enone **1 f** with HD surrogate **5**.

The InBr_3_ catalyst could first coordinate to the alkynyl enone **1 f**, forming the intermediate complex **A** with indium interacting with the carbonyl oxygen atom and the alkynyl moiety. Then a hydride, originating from the HD surrogate **5**, is transferred in terms of a Michael addition to the activated alkenone moiety resulting in the formation of the Wheland intermediate **B**. This hydride transfer is either catalysed by InBr_3_, via a [H−InBr_3_]^−^ reactive intermediate, similar to the indium hydride published by Baba,^[10]^ or by direct hydride transfer from **5** to the activated enone **A**. The cyclisation of the carbonyl oxygen onto the alkynyl moiety leads to the formation of the furan ring with covalently bound indium at the 3‐position in the intermediate complex **C**. The Wheland complex **B**, which is a strong proton donor, then replaces the InBr_3_ moiety by a proton to afford product **7**. Compared to other transfer hydrogenations of dihydroaromatic surrogates catalysed by Lewis acids, this proposed mechanism would be unprecedented.

When the transfer hydrogenation of alkenes was discussed in the literature,[^6^, ^7^] the addition of hydride and proton was reversed; the first steps in these reaction mechanisms were the protonation of the alkene starting materials by the Wheland complex to afford stabilised carbenium ions followed by hydride transfers from the surrogate to the carbenium ions. Accordingly, an alternative reaction mechanism would start with the protonation of the starting material **1 f** by the Wheland complex **B** towards intermediate **D** (Scheme 6).

**Scheme 6:**
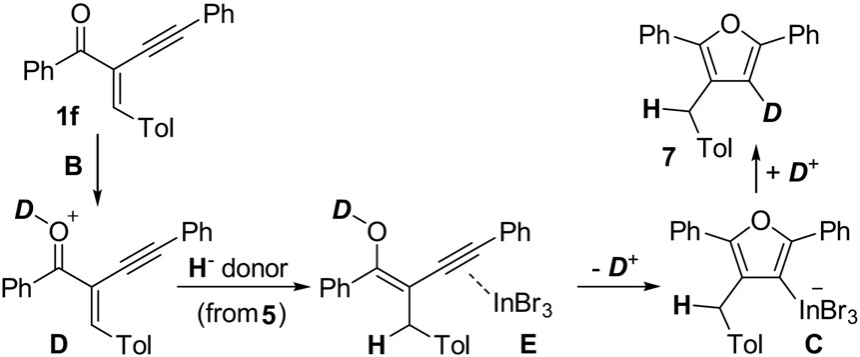
Alternative mechanism based on the deuterium‐labelling experiments for the InBr_3_‐catalysed cyclisation of enone **1 f** with HD surrogate **5**.

An InBr_3_‐assisted cyclisation via a coordinative indium‐bonded intermediate **E** towards **C** would result in the formation of the furan backbone in **7** but could also account for the loss of considerable amounts of deuterium labelling via dedeuteration/redeuteration when going from intermediate **E** via **C** to the desired product **7**. Even small amounts of a proton source (e.g. H_2_O) could result in a considerable loss of deuterium incorporation in product **7**.

Based on these considerations outlined in Scheme 6, the reaction of **1 f** with the HD surrogate **4** (Scheme 4) should not show a loss of deuterium labelling in product **6** when small amounts of H_2_O are present. In an attempt to verify the hydrogen/deuterium scrambling from intermediate **E** via **C** to the furan product **7**, the control experiment as outlined in Scheme 7 was conducted.

**Scheme 7:**
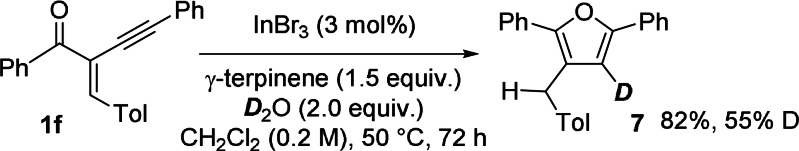
InBr_3_‐catalysed hydrodeuterogenation of **1 f** utilising γ‐terpinene and D_2_O as deuterium source.

Fortunately, InBr_3_‐catalysed transfer hydrogenations are not highly sensitive to traces of H_2_O (or D_2_O)[^5b^, ^5c^, ^5d^, ^5e^, ^5f^, ^5g^] although the reactivity of the catalyst was significantly diminished so that a prolonged reaction time was needed. As expected, the proton from the Wheland complex derived from γ‐terpinene (corresponding to **B**) protonated the starting material **1 f** and the transfer hydrogenation from γ‐terpinene to the enone moiety in **D** generated the intermediate **E** without any incorporation of deuterium next to the tolyl substituent. In the experiment shown in Scheme 7 the hydrogen/deuterium scrambling took place and the product **7** was isolated in 82 % with 55 % deuterium incorporation in the 3‐position. In a control experiment, the H‐labelled product **3 f** was reacted with D_2_O in the presence of InBr_3_ but no deuterated product **7** was detected, indicating that the loss of the deuterium labelling in the original reaction (**1 f**→**7**, Scheme 4) might be associated with traces of H_2_O.

In conclusion, we developed an InBr_3_‐catalysed cyclisation of alkynyl enones utilising γ‐terpinene as H_2_ surrogate. The optimisation of the reaction with the *Design of Experiments* (DoE) approach identified the crucial reaction parameters. This transition‐metal‐free reaction tolerated a wide range of aromatic and aliphatic functional groups and provided the substituted furans in high yields under mild reaction conditions and low catalyst loading. Moreover, deuterium‐labelling studies utilising regioselectively substituted dihydroaromatic compounds as HD surrogates were conducted to gain insights into the reaction mechanism.

## Conflict of interest

The authors declare no conflict of interest.

## Supporting information

As a service to our authors and readers, this journal provides supporting information supplied by the authors. Such materials are peer reviewed and may be re‐organized for online delivery, but are not copy‐edited or typeset. Technical support issues arising from supporting information (other than missing files) should be addressed to the authors.

Supporting Information
