# Supplementary material for: Indium‐Catalysed Transfer Hydrogenation for the Reductive Cyclisation of 2‐Alkynyl Enones towards Trisubstituted Furans
Source: Angew Chem Int Ed Engl. 2021 Oct 1;60(44):23661–6. doi: 10.1002/anie.202109266 (PMC8597135; doi:10.1002/anie.202109266)
Supplement: Supplementary file 1 — Supporting Information [file ANIE-60-23661-s001.pdf]

Angewandte Chemie
Eine Zeitschrift der Gesellschaft Deutscher Chemiker

Supporting Information

Indium-Catalysed Transfer Hydrogenation for the Reductive Cyclisation of 2-Alkynyl Enones towards Trisubstituted Furans

*Luomo Li, Sascha Kail, Sebastian M. Weber, and Gerhard Hilt**

anie_202109266_sm_miscellaneous_information.pdf

Supporting Information

Contents

1 General Information **1**

2 Preparation of Substrates **2**

3 $InBr_3$-catalysed Transfer-Hydrogenation for the Synthesis of Furans **14**

4 Isotope Labeling Investigation **29**

5 Reaction Optimization **31**

6 References **35**

7 Spectra **36**

1 General Information

All solvents were purified prior to use by common techniques. All anhydrous solvents were dried over molecular sieve (3 Å) and stored under nitrogen atmosphere. All reactions with air and/or moisture sensitive substances were carried out under a nitrogen atmosphere using standard Schlenk techniques with magnetic stirring. Reagents obtained from commercial sources were used without further purifications. Non-commercial reagents were prepared according to literature-known procedures.

Thin layer chromatography (TLC) was carried out on prefabricated plates (silica gel 60, F254 with fluorescence indicator) by Macherey-Nagel and visualised by fluorescence quenching under UV-light. In addition, TLC-plates were stained using a cerium sulfate/phosphomolybdic acid stain ($Ce(SO_4)_2$: 2.0 g, conc. H_2SO_4: 50 mL, $(NH_4)_6Mo_7O_{24}\cdot 4H_2O$: 50 g, H_2O: 400 mL), potassium permanganate stain (K_2CO_3: 60.0 g, $KMnO_4$: 9.0 g, H_2O: 900 mL, acetic acid: 1 mL) or an I_2 stain.

Column chromatography was performed with silica gel 60 M (40-63 µm, 230-400 mesh) from Macherey-Nagel as solid phase with the indicated solvent system.

High resolution mass spectra (EI) were recorded on a Thermo Scientific DFS spectrometer. The ionization was accomplished by electron ionization (EI) at an energy of 70 eV. IR spectra were recorded on a Shimadzu IR Spirit T spectrophotometer equipped with a diamond ATR unit. The absorption bands are given in wave number (cm^{-1}).

^{1}H NMR, ^{19}F NMR and proton decoupled ^{13}C NMR spectra were recorded on *Bruker Fourier* 300HD, or a Bruker Avance III 500HD spectrometer at ambient temperature utilizing pre-set pulse programs. The chemical shifts are given in parts per million (ppm). NMR standards were used as follows: ^{1}H NMR spectroscopy: δ = 7.26 ppm ($CDCl_3$), δ = 2.50 ppm (DMSO-d_6). ^{19}F NMR spectroscopy: δ = 0.0 ppm ($CFCl_3$). ^{13}C NMR spectroscopy: δ = 77.16 ppm ($CDCl_3$), δ = 39.52 ppm (DMSO-d_6). Data are reported as follows: s = singlet, d = doublet, t = triplet, q = quartet, m = multiplet.

Melting points were determined on a Schorpp Gerätetechnik MPM-HV3 melting point apparatus.

2 Preparation of Substrates

The known alkynyl enones **1a**, **1k**, **1n**, **1o**, **1q**, **1s**, and **1x** were synthesized according to the literature procedures.[1-6] Unknown alkynyl enones were prepared from the corresponding α-bromo-α,β-unsaturated ketones and alkynes using the same method (see General Procedure 1).

General Procedure 1

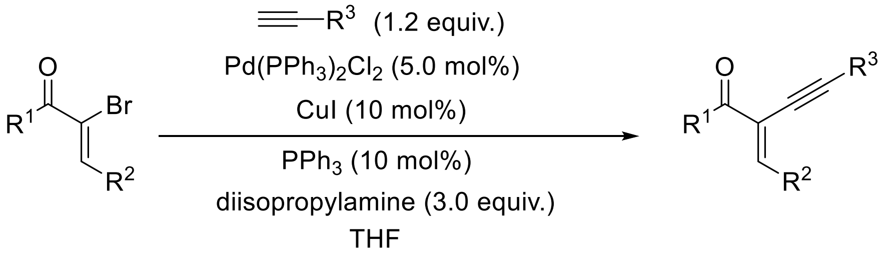

Under N_2 atmosphere $Pd(PPh_3)_2Cl_2$ (175 mg, 0.25 mmol, 5.0 mol%), CuI (95.0 mg, 0.50 mmol, 10 mol%), PPh_3 (131 mg, 0.50 mmol, 10 mol%), diisopropylamine (2.10 mL, 1.50 mmol, 3.0 eq.) and α-bromo-α,β-enone (5.0 mmol, 1.0 equiv.) were dissolved in THF (20 mL). After the mixture was cooled to 0 °C, the corresponding alkyne (6.0 mmol, 1.2 equiv.) was added dropwise. The mixture was warmed to ambient temperature, stirred, and the conversion was monitored by TLC. Upon completion, the reaction mixture was diluted with H_2O (40 mL) and the phases were separated. The aqueous phase was extracted with Et_2O (3 x 40 mL). The combined organic phase was washed with 1 M HCl, water, and brine and dried over $MgSO_4$. Then, it was filtered and the solvent was evaporated under reduced pressure. The crude product was purified by flash column chromatography (SiO_2, *n*-pentane/diethyl ether) to afford the alkynyl enone **1**.

2-Benzylidene-1-(4-methoxyphenyl)-4-phenylbut-3-yn-1-one (1b)

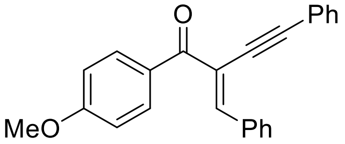

According to the general procedure 1 the title compound was prepared using 2-bromo-1-(4-methoxyphenyl)-3-phenylprop-2-en-1-one (1.59 g, 5.0 mmol, 1.0 equiv.) and phenyl acetylene (0.671 mL, 6.0 mmol, 1.2 equiv.). The product was obtained as a yellow solid (1.45 g, 4.3 mmol, 86%).

R_f = 0.23 (*n*-pentane:diethyl ether = 20:1).

^{1}H NMR (500 MHz, $CDCl_3$): δ = 8.17–8.02 (m, 4H), 7.56 (s, 1H), 7.50–7.40 (m, 5H), 7.39–7.31 (m, 3H), 7.02–6.95 (m, 2H), 3.90 (s, 3H) ppm. **^{13}C NMR** (126 MHz, $CDCl_3$): δ = 191.9, 163.5, 144.2, 135.2, 132.5, 131.5, 130.43, 130.35, 129.8, 128.9, 128.7, 128.6, 123.2, 121.5, 113.5, 100.9, 87.5, 55.6 ppm.

MS: (EI^+) m/z = 338.0, 321.0, 307.0, 295.0, 277.0, 261.0, 238.0, 231.0, 210.0, 202.0, 165.0, 149.0, 135.0, 107.0, 92.0, 77.0, 57.0, 44.0.

HRMS (EI^+) m/z for $C_{24}H_{18}O_2$ [M^+]: calc.: 338.1301, found: 338.1292.

IR (ATR, neat): $\tilde{\nu}$ = 3053, 2742, 1653, 1596, 1563, 1512, 1487, 1464, 1442, 1420, 1317, 1307, 1256, 1207, 1199, 1169, 1094, 1020, 1000, 963, 950, 924, 904, 853, 813, 773, 764, 757, 744, 681, 639, 621, 553, 547, 520, 510 cm^{-1}.

2-Benzylidene-1-(4-fluorophenyl)-4-phenylbut-3-yn-1-one (1c)

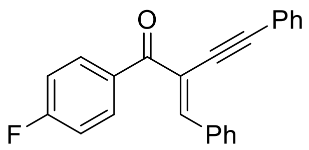

According to the general procedure 1 the title compound was prepared using 2-bromo-1-(4-fluorophenyl)-3-phenylprop-2-en-1-one (1.53 g, 5.0 mmol, 1.0 equiv.) and phenyl acetylene (0.671 mL, 6.0 mmol, 1.2 equiv.). The product was obtained as a yellow solid (1.26 g, 3.9 mmol, 77%).

R_f = 0.55 (*n*-pentane:diethyl ether = 20:1).

^{1}H NMR (500 MHz, $CDCl_3$): δ = 8.18–8.11 (m, 2H), 8.11–8.03 (m, 2H), 7.63 (s, 1H), 7.51–7.44 (m, 3H), 7.42–7.39 (m, 2H), 7.38–7.33 (m, 3H), 7.19–7.14 (m, 2H) ppm. **^{13}C NMR** (126 MHz, $CDCl_3$): δ = 191.9, 165.7 (d, J = 254.2 Hz), 145.2, 135.1, 133.6 (d, J = 2.9 Hz), 132.7 (d, J = 9.2 Hz), 131.6, 130.9, 130.6, 129.2, 128.9, 128.8, 123.0, 120.9, 115.5 (d, J = 21.8 Hz), 101.4, 87.4 ppm. **^{19}F NMR** (470 MHz, $CDCl_3$): δ = -105.69 ppm.

MS: (EI^+) m/z = 326.0, 309.0, 297.0, 277.0, 249.0, 231.0, 221.0, 202.0, 196.0, 176.0, 151.0, 123.0, 105.0, 95.0, 77.0, 69.0, 51.0.

HRMS (EI^+) m/z for $C_{23}H_{17}OF$ [M^+]: calc.: 326.1101, found: 326.1102.

IR (ATR, neat): $\tilde{\nu}$ = 3060, 3027, 2189, 1717, 1664, 1596, 1564, 1506, 1487, 1449, 1440, 1409, 1316, 1306, 1263, 1230, 1203, 1187, 1154, 1090, 1069, 1026, 1014, 999, 963, 930, 853, 823, 774, 761, 753, 690, 679, 639, 620, 556, 546, 520, 504 cm^{-1}.

1-(4-Fluorophenyl)-2-(4-methoxybenzylidene)-4-phenylbut-3-yn-1-one (1d)

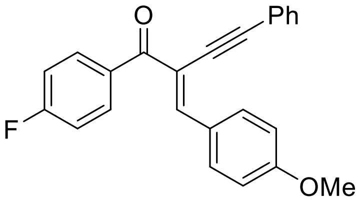

According to the general procedure 1 the title compound was prepared using 2-bromo-1-(4-fluorophenyl)-3-(4-methoxyphenyl)prop-2-en-1-one (1.68 g, 5.0 mmol, 1.0 equiv.) and phenyl acetylene (0.671 mL, 6.0 mmol, 1.2 equiv.). The product was obtained as a yellow solid (962 mg, 2.7 mmol, 54%).

R*f* = 0.3 (*n*-pentane:diethyl ether = 20:1).

^{1}H NMR (500 MHz, $CDCl_3$): δ = 8.15–8.10 (m, 2H), 8.09–8.01 (m, 2H), 7.65 (s, 1H), 7.43–7.38 (m, 2H), 7.38–7.31 (m, 3H), 7.18–7.13 (m, 2H), 7.01–6.95 (m, 2H), 3.89 (s, 3H) ppm. **^{13}C NMR** (126 MHz, $CDCl_3$): δ = 192.1, 165.4 (d, *J* = 253.5 Hz), 161.9, 145.4, 133.9 (d, *J* = 3.0 Hz), 132.7, 132.4 (d, *J* = 9.1 Hz), 131.4, 128.9, 128.7, 127.9, 123.2, 118.0, 115.2 (d, *J* = 21.8 Hz), 114.3, 100.8, 87.8, 55.6 ppm. **^{19}F NMR** (470 MHz, $CDCl_3$): δ = -106.31 ppm.

MS: (EI^+) m/z = 356.0, 339.0, 325.0, 313.0, 297.0, 279.0, 261.0, 256.0, 233.0, 218.0, 202.0, 189.0, 161.0, 135.0, 123.0, 115.0, 95.0, 77.0, 44.0.

HRMS (EI^+) m/z for $C_{24}H_{17}O_2F$ [M^+]: calc.: 356.1207, found: 356.1203.

IR (ATR, neat): $\tilde{\nu}$ = 3062, 3021, 2973, 2934, 2843, 1824, 1659, 1596, 1553, 1512, 1506, 1489, 1464, 1443, 1422, 1409, 1337, 1320, 1312, 1303, 1297, 1257, 1236, 1223, 1206, 1177, 1159, 1092, 1070, 1031, 1016, 999, 984, 966, 944, 913, 854, 843, 824, 803, 797, 761, 751, 721, 687, 634, 617, 603, 586, 541, 523, 507 cm^{-1}.

2-(4-Methoxybenzylidene)-1,4-diphenylbut-3-yn-1-one (1e)

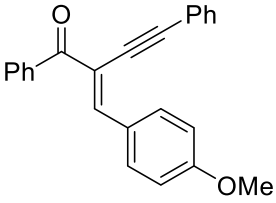

According to the general procedure 1 the title compound was prepared using 2-bromo-3-(4-methoxyphenyl)-1-phenylprop-2-en-1-one (1.59 g, 5.0 mmol, 1.0 equiv.) and phenyl acetylene (0.671 mL, 6.0 mmol, 1.2 equiv.). The product was obtained as a yellow solid (1.18 g, 3.5 mmol, 70%).

R*f* = 0.23 (*n*-pentane:diethyl ether = 20:1).

^{1}H NMR (500 MHz, $CDCl_3$): δ = 8.16–8.12 (m, 2H), 8.03–7.93 (m, 2H), 7.65 (s, 1H), 7.59–7.55 (m, 1H), 7.50–7.46 (m, 2H), 7.42–7.38 (m, 2H), 7.37–7.30 (m, 3H), 7.03–6.92 (m, 2H),

3.88 (s, 3H) ppm. **^{13}C NMR** (126 MHz, $CDCl_3$): δ = 193.7, 161.8, 145.4, 137.8, 132.6, 132.3, 131.4, 129.7, 128.8, 128.6, 128.1, 127.9, 123.3, 118.4, 114.3, 100.5, 87.8, 55.6 ppm.

MS: (EI^+) m/z = 338.0, 321.0, 307.0, 295.0, 279.0, 261.0, 252.0, 233.0, 218.0, 202.0, 189.0, 165.0, 149.0, 127.1, 115.0, 105.0, 97.1, 77.0, 57.0, 44.0.

HRMS (EI^+) m/z for $C_{24}H_{18}O_2$ [M^+]: calc.: 338.1301, found: 338.1296.

IR (ATR, neat): $\tilde{\nu}$ = 3026, 3002, 2933, 2836, 1879, 1663, 1597, 1580, 1557, 1510, 1487, 1449, 1440, 1423, 1386, 1366, 1320, 1309, 1257, 1226, 1174, 1093, 1069, 1024, 999, 963, 939, 926, 911, 896, 861, 820, 784, 754, 739, 726, 707, 687, 667, 629, 621, 583, 559, 541, 530, 523 cm^{-1}.

(*E, Z*)-2-(4-Methylbenzylidene)-1,4-diphenylbut-3-yn-1-one (1f)

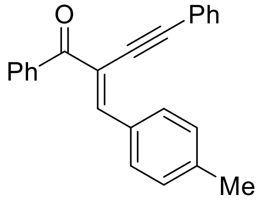

According to the general procedure 1 the title compound was prepared using 2-bromo-1-phenyl-3-(*p*-tolyl)prop-2-en-1-one (1.51 g, 5.0 mmol, 1.0 equiv.) and phenyl acetylene (0.671 mL, 6.0 mmol, 1.2 equiv.). The product was obtained as a yellow solid (1.27 g, 3.9 mmol, 79%).

R_f = 0.48 (*n*-pentane:diethyl ether = 20:1).

^{1}H NMR (500 MHz, $CDCl_3$): Major product: δ = 8.06–8.02 (m, 2H), 8.02–7.97 (m, 2H), 7.64 (s, 1H), 7.60–7.54 (m, 2H), 7.51–7.47 (m, 2H), 7.42–7.37 (m, 2H), 7.36–7.32 (m, 3H), 7.31–7.26 (m, 2H), 2.43 (s, 3H) ppm. Minor product: δ = 8.17–8.07 (m, 2H), 7.58 (s, 1H), 7.47–7.43 (m, 2H), 7.42–7.37 (m, 2H), 7.31–7.26 (m, 4H), 7.18–7.14 (m, 2H), 7.05–6.97 (m, 2H), 2.26 (s, 3H) ppm. **^{13}C NMR** (126 MHz, $CDCl_3$): Major product: δ = 193.6, 145.5, 140.4, 137.6, 134.0, 132.5, 132.4, 131.4, 130.6, 129.8, 129.5, 128.6, 128.2, 123.2, 120.0, 100.8, 87.6, 21.8 ppm. Minor product: δ = 194.4, 141.4, 139.3, 135.4, 134.0, 131.9, 131.7, 130.1, 129.4, 129.1, 128.8, 128.6, 128.4, 123.0, 122.4, 94.1, 88.1, 21.4 ppm.

MS: (EI^+) m/z = 322.0, 207.0, 279.0, 245.0, 341.0, 217.0, 217.0, 202.0, 189.0, 178.0, 165.0, 139.0, 115.0, 105.0, 91.0, 77.0, 65.0, 51.0.

HRMS (EI^+) m/z for $C_{24}H_{18}O$ [M^+]: calc.: 322.1352, found: 322.1347.

IR (ATR, neat): $\tilde{\nu}$ = 3054, 3026, 2917, 1657, 1596, 1576, 1554, 1010, 1489, 1446, 1412, 1680, 1333, 1319, 1264, 1219, 1206, 1179, 1156, 1094, 1067, 1031, 1016, 1000, 984, 964, 863, 810, 791, 771, 751, 709, 686, 659, 634, 623, 616, 551, 519 cm^{-1}.

2-(4-Chlorobenzylidene)-1,4-diphenylbut-3-yn-1-one (1g)

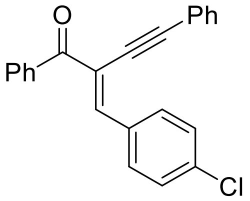

According to the general procedure 1 the title compound was prepared using 2-bromo-3-(4-chlorophenyl)-1-phenylprop-2-en-1-one (1.61 g, 5.0 mmol, 1.0 equiv.) and phenyl acetylene (0.671 mL, 6.0 mmol, 1.2 equiv.). The product was obtained as a yellow solid (1.25 g, 3.6 mmol, 73%).

R*f* = 0.58 (*n*-pentane:diethyl ether = 20:1).

^{1}H NMR (500 MHz, $CDCl_3$): δ = 8.08–8.04 (m, 2H), 8.03–7.97 (m, 2H), 7.62–7.56 (m, 2H), 7.51–7.47 (m, 2H), 7.45–7.42 (m, 2H), 7.40–7.37 (m, 2H), 7.36–7.32 (m, 3H) ppm. **^{13}C NMR** (126 MHz, $CDCl_3$): δ = 193.1, 143.4, 137.2, 136.5, 133.5, 132.8, 131.6, 131.5, 129.9, 129.2, 129.0, 128.7, 128.3, 122.8, 121.6, 101.8, 87.1 ppm.

MS: (EI^+) m/z = 342.0, 207.0, 279.0, 264.9, 237.0, 202.0, 176.0, 165.0, 135.0, 126.0, 105.0, 77.0, 51.0.

HRMS (EI^+) m/z for $C_{23}H_{15}OCl$ [M^+]: calc.: 342.0806, found: 342.0799.

IR (ATR, neat): $\tilde{\nu}$ = 3057, 2190, 1667, 1596, 1576, 1554, 1486, 1447, 1442, 1406, 1320, 1309, 1203, 1180, 1157, 1090, 1027, 1011, 999, 964, 946, 927, 920, 819, 794, 787, 753, 717, 706, 687, 641, 616, 550, 519 cm^{-1}.

1,4-Diphenyl-2-(thiophen-2-ylmethylene)but-3-yn-1-one (1h)

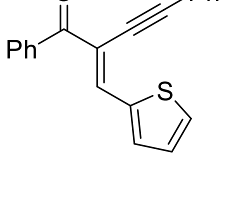

According to the general procedure 1 the title compound was prepared using 2-bromo-1-phenyl-3-(thiophen-2-yl)prop-2-en-1-one (1.47 g, 5.0 mmol, 1.0 equiv.) and phenyl acetylene (0.671 mL, 6.0 mmol, 1.2 equiv.). The product was obtained as a yellow solid (903 mg, 2.9 mmol, 58%).

R*f* = 0.75 (*n*-pentane:diethyl ether = 20:1).

^{1}H NMR (500 MHz, $CDCl_3$): δ = 8.07–7.94 (m, 3H), 7.63–7.55 (m, 3H), 7.53–7.46 (m, 4H), 7.38–7.34 (m, 3H), 7.16 (dd, *J* = 5.1, 3.7 Hz, 1H) ppm. **^{13}C NMR** (126 MHz, $CDCl_3$): δ = 192.5, 139.7, 139.2, 137.8, 135.0, 132.4, 131.5, 131.4, 129.7, 129.0, 128.6, 128.1, 127.4,

123.2, 117.7, 103.8, 87.9 ppm.

MS: (EI^+) m/z = 314.2, 297.2, 281.1, 253.2, 237.1, 208.1, 202.1, 176.1, 165.1, 150.1, 139.1, 126.1, 105.1, 77.0, 63.0, 51.0.

HRMS (EI^+) m/z for $C_{21}H_{14}OS$ [M^+]: calc.: 314.0760, found: 314.0770.

IR (ATR, neat): $\tilde{\nu}$ = 3057, 1653, 1596, 1589, 1570, 1553, 1512, 1500, 1487, 1446, 1416, 1339, 1314, 1264, 1210, 1177, 1159, 1096, 1070, 1053, 1024, 1014, 1000, 969, 953, 939, 914, 857, 826, 796, 774, 754, 710, 687, 674, 656, 639, 584, 573, 560 cm^{-1}.

2-Benzylidene-1-phenyl-4-(4-(trifluoromethyl)phenyl)but-3-yn-1-one (1i)

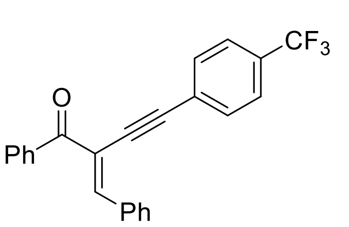

According to the general procedure 1 the title compound was prepared using 2-bromo-1,3-diphenylprop-2-en-1-one (1.47 g, 5.0 mmol, 1.0 equiv.) and 1-ethynyl-4-(trifluoromethyl)benzene (1.02 g, 6.0 mmol, 1.2 equiv.). The product was obtained as a yellow solid (1.64 g, 4.4 mmol, 87%).

$\mathbf{R}_f$ = 0.63 (*n*-pentane:diethyl ether = 20:1).

^{1}H NMR (500 MHz, $CDCl_3$): δ = 8.13–8.05 (m, 2H), 8.03–7.96 (m, 2H), 7.69 (s, 1H), 7.63–7.56 (m, 3H), 7.52–7.45 (m, 7H) ppm. **^{13}C NMR** (126 MHz, $CDCl_3$): δ = 193.2, 146.5, 137.2, 134.8, 132.7, 131.7, 131.1, 130.6, 129.8, 128.8, 128.3, 125.5 (q, *J* = 3.8 Hz), 99.1, 89.5 ppm. The signal for the CF_3 carbon was not detectable. **^{19}F NMR** (470 MHz, $CDCl_3$): δ = -62.92 ppm.

MS: (EI^+) m/z = 376.0, 359.0, 347.0, 322.0, 299.0, 271.0, 251.0, 231.0, 220.0, 202.0, 178.0, 167.0, 149.0, 131.0, 105.0, 77.0, 57.0, 44.0.

HRMS: (EI^+) m/z for $C_{24}H_{15}OF_3$ [M^+]: calc.: 376.1070, found: 376.1069.

IR (ATR, neat): $\tilde{\nu}$ = 3060, 3027, 2193, 1807, 1670, 1611, 1599, 1579, 1563, 1513, 1492, 1446, 1404, 1320, 1266, 1203, 1179, 1163, 1124, 1102, 1063, 1026, 1016, 1001, 963, 933, 927, 904, 839, 784, 767, 754, 736, 716, 694, 683, 670, 641, 623, 611, 594, 564, 519 cm^{-1}.

2-Benzylidene-4-(4-bromophenyl)-1-phenylbut-3-yn-1-one (1j)

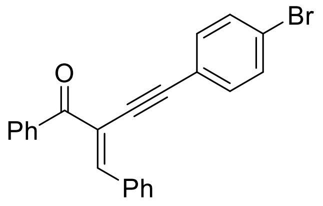

According to the general procedure 1 the title compound was prepared using 2-bromo-1,3-diphenylprop-2-en-1-one (1.47 g, 5.0 mmol, 1.0 equiv.) and 1-bromo-4-ethynylbenzene (1.09 g, 6.0 mmol, 1.2 equiv.). The product was obtained as a yellow solid (1.86 g, 4.8 mmol, 96%).

R*f* = 0.50 (*n*-pentane:diethyl ether = 20:1).

^{1}H NMR (500 MHz, $CDCl_3$): δ = 8.12–8.07 (m, 2H), 8.02–7.94 (m, 2H), 7.65 (s, 1H), 7.62–7.57 (m, 1H), 7.52–7.44 (m, 7H), 7.29–7.22 (m, 2H) ppm. **^{13}C NMR** (126 MHz, $CDCl_3$): δ = 193.3, 145. 7, 137.3, 134.9, 132.9, 132.7, 131.9, 130.9, 130.5, 129.8, 128.8, 128.3, 123.4, 121.9, 120.9, 99.7, 88.4 ppm.

MS: (EI^+) m/z = 387.9, 370.9, 359.8, 342.9, 307.0, 279.0, 252.0, 231.0, 202.0, 174.0, 150.0, 131.0, 105.0, 77.0, 51.0.

HRMS: (EI^+) m/z for $C_{13}H_{15}OBr$ [M^+]: calc.: 386.0301, found: 386.0293.

IR (ATR, neat): $\tilde{\nu}$ = 3303, 3060, 3023, 2194, 1907, 1793, 1663, 1599, 1589, 1577, 1562, 1492, 1483, 1446, 1390, 1337, 1323, 1313, 1269, 1202, 1184, 1176, 1157, 1104, 1093, 1066, 1027, 1004, 966, 924, 897, 846, 827, 810, 801, 783, 747, 719, 704, 690, 680, 673, 634, 630, 614, 561 cm^{-1}.

2-Benzylidene-1-phenyl-4-(*m*-tolyl)but-3-yn-1-one (1l)

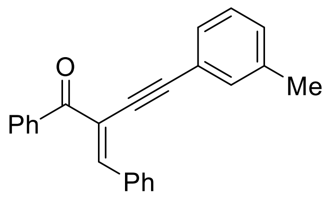

According to the general procedure 1 the title compound was prepared using 2-bromo-1,3-diphenylprop-2-en-1-one (1.47 g, 5.0 mmol, 1.0 equiv.) and 1-ethynyl-3-methylbenzene (697 mg, 6.0 mmol, 1.2 equiv.). The product was obtained as a yellow solid (1.45 g, 4.5 mmol, 90%).

R*f* = 0.53 (*n*-pentane:diethyl ether = 20:1).

^{1}H NMR (500 MHz, $CDCl_3$): δ = 8.19–8.08 (m, 2H), 8.08–7.94 (m, 2H), 7.61 (s, 1H), 7.61–7.56 (m, 1H), 7.51–7.41 (m, 5H), 7.24–7.14 (m, 4H), 2.34 (s, 3H) ppm. **^{13}C NMR** (126 MHz, $CDCl_3$): δ = 193.5, 145.0, 138.3, 137.4, 135.1, 132.6, 132.1, 130.7, 130.5, 129.9, 128.7, 128.6, 128.5, 128.2, 122.9, 121.2, 101.3, 87.0, 21.4 ppm.

MS: (EI^+) m/z = 322.0, 307.0, 293.0, 279.0, 245.0, 231.0, 215.0, 202.0, 189.0, 167.0, 139.0, 119.0, 105.0, 91.0, 77.0, 65.0, 51.0.

HRMS (EI^+) m/z for $C_{24}H_{18}O$ [M^+]: calc.: 322.1352, found: 322.1347.

IR (ATR, neat): $\tilde{\nu}$ =3053, 3024, 2919, 1709, 1654, 1596, 1577, 1484, 1446, 1320, 1294, 1252, 1229, 1213, 1177, 1109, 1073, 1026, 1000, 957, 927, 876, 784, 746, 726, 689, 669, 616, 587, 560 cm^{-1}.

2-Benzylidene-4-(4-methoxyphenyl)-1-phenylbut-3-yn-1-one (1m)

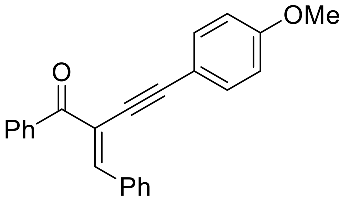

According to the general procedure 1 the title compound was prepared using 2-bromo-1,3-diphenylprop-2-en-1-one (1.47 g, 5.0 mmol, 1.0 equiv.) and 1-ethynyl-4-methoxybenzene (0.778 mL, 6.0 mmol, 1.2 equiv.). The product was obtained as a brown liquid (1.69 g, 5.0 mmol, 99%).

$\mathbf{R}_f$ = 0.23 (*n*-pentane:diethyl ether = 20:1).

^{1}H NMR (500 MHz, $CDCl_3$): δ = 8.15–8.10 (m, 2H), 8.05–7.96 (m, 2H), 7.62–7.55 (m, 2H), 7.51–7.41 (m, 5H), 7.36–7.31 (m, 2H), 6.90–6.84 (m, 2H), 3.82 (s, 3H) ppm. **^{13}C NMR** (126 MHz, $CDCl_3$): δ = 193.6, 160.3, 144.2, 137.4, 135.2, 133.0, 132.5, 130.5, 130.4, 129.9, 128.7, 128.2, 121.4, 115.2, 114.3, 101. 4, 86.3, 55.5 ppm.

MS: (EI^+) m/z = 338.0, 323.0, 307.0, 295.0, 278.0, 261.0, 252.0, 233.0, 218.0, 202.0, 189.0, 183.0, 159.0, 135.0, 126.9, 115.0, 105.0, 92.0, 77.0, 51.0.

HRMS (EI^+) m/z for $C_{24}H_{18}O_2$ [M^+]: calc.: 338.1301, found: 338.1297.

IR (ATR, neat): $\tilde{\nu}$ = 3057, 2957, 2933, 2837, 2540, 2190, 1660, 1604, 1597, 1564, 1507, 1492, 1463, 1446, 1414, 1394, 1317, 1303, 1290, 1246, 1204, 1170, 1106, 1094, 1072, 1026, 1001, 961, 941, 924, 829, 806, 790, 773, 753, 716, 687, 671, 641, 616, 553, 531 cm^{-1}.

3-(2-Chlorobenzylidene)-5-phenylpent-4-yn-2-one (1p)

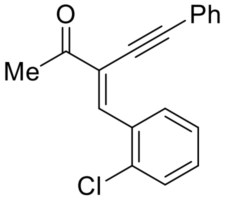

According to the general procedure 1 the title compound was prepared using 3-bromo-4-(2-chlorophenyl)but-3-en-2-one (1.30 g, 5.0 mmol, 1.0 equiv.) and phenyl acetylene (0.671 mL, 6.0 mmol, 1.2 equiv.). The product was obtained as a yellow solid (828 mg, 2.7 mmol, 59%).

$\mathbf{R}_f$ = 0.50 (*n*-pentane:diethyl ether = 20:1).

^{1}**H NMR** (500 MHz, $CDCl_3$): δ = 8.60–8.39 (m, 1H), 8.19 (s, 1H), 7.51–7.44 (m, 3H), 7.42–7.36 (m, 3H), 7.36–7.32 (m, 2H), 2.63 (s, 3H) ppm. ^{13}**C NMR** (126 MHz, $CDCl_3$): δ = 195.8, 139.0, 136.2, 132.9, 131.6, 131.4, 130.2, 130.0, 129.2, 128.7, 126.6, 122.7, 122.6, 99.1, 86.3, 28.2 ppm.

MS (EI^+) m/z = 280.0, 265.0, 245.0, 237.0, 215.0, 202.0, 175.0, 151.0, 126.0, 115.0, 105.0, 83.0, 77.0, 51.0, 43.0.

HRMS (EI^+) m/z for $C_{18}H_{13}OCl$ [M^+]: calc.: 280.0649, found: 280.0643.

IR (ATR, neat): $\tilde{\nu}$ = 3079, 3044, 2192, 1692, 1597, 1572, 1559, 1489, 1469, 1442, 1354, 1293, 1247, 1224, 1186, 1126, 1069, 1044, 1021, 996, 974, 953, 913, 903, 861, 846, 770, 756, 749, 821, 714, 690, 684, 627, 564, 546, 526, 520 cm^{-1}.

3-(Benzo[*d*][1,3]dioxol-5-ylmethylene)-5-phenylpent-4-yn-2-one (1r)

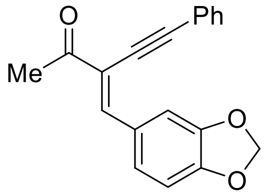

According to the general procedure 1 the title compound was prepared using 4-(benzo[*d*][1,3]dioxol-5-yl)-3-bromobut-3-en-2-one (1.34 g, 5.0 mmol, 1.0 equiv.) and phenyl acetylene (0.671 mL, 6.0 mmol, 1.2 equiv.). The product was obtained as a yellow solid (802 mg, 2.8 mmol, 55%).

$\mathbf{R_f}$ = 0.28 (*n*-pentane:diethyl ether = 20:1).

^{1}**H NMR** (500 MHz, $CDCl_3$): δ = 7.93 (d, *J* = 1.7 Hz, 1H), 7.74 (s, 1H), 7.60–7.51 (m, 2H), 7.44 (dd, *J* = 8.3, 1.8 Hz, 1H), 7.43–7.33 (m, 3H), 6.87 (d, *J* = 8.1 Hz, 1H), 6.03 (s, 2H), 2.60 (s, 3H) ppm. ^{13}**C NMR** (126 MHz, $CDCl_3$): δ = 196.3, 150.1, 148.1, 142.7, 131.4, 129.2, 129.0, 128.8, 128.1, 123.1, 118.0, 109.3, 108.6, 101.8, 99.5, 87.5, 28.2 ppm.

MS (EI^+) m/z = 290.0, 275.0, 260.0, 247.0, 232.0, 217.0, 203.0, 189.0, 163.0, 151.0, 139.0, 126.0, 115.0, 105.0, 87.0, 77.0, 63.0, 43.0.

HRMS (EI^+) m/z for $C_{24}H_{18}O_2$ [M^+]: calc.: 290.0937, found: 290.0934.

IR (ATR, neat): $\tilde{\nu}$ = 3096, 3077, 3054, 3003, 2957, 2907, 2787, 1687, 1617, 1604, 1560, 1504, 1486, 1416, 1354, 1264, 1213, 1199, 1186, 1179, 1103, 1069, 1034, 1019, 974, 940, 924, 916, 866, 841, 813, 783, 754, 726, 686, 633, 627, 614, 597, 554, 541, 527, 519, 507 cm^{-1}.

2-Benzylidene-5-methyl-1-phenylhex-3-yn-1-one (1t)

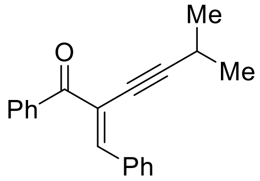

According to the general procedure 1 the title compound was prepared using 2-bromo-1,3-diphenylprop-2-en-1-one (1.47 g, 5.0 mmol, 1.0 equiv.) and 3-methyl-1-butyne (0.645 mL, 6.0 mmol, 1.2 equiv.). The product was obtained as a brown liquid (1.06 g, 3.9 mmol, 77%).

R$_f$ = 0.53 (*n*-pentane:diethyl ether = 20:1).

^{1}H NMR (500 MHz, $CDCl_3$): δ = 8.09–8.02 (m, 2H), 7.95–7.91 (m, 2H), 7.57–7.53 (m, 1H), 7.48–7.38 (m, 6H), 2.81 (hept, *J* = 6.9 Hz, 1H), 1.22 (d, *J* = 6.9 Hz, 6H) ppm. **^{13}C NMR** (126 MHz, $CDCl_3$): δ = 194.3, 144.1, 137.4, 135.1, 132.4, 130.4, 130.2, 129.9, 128.5, 128.1, 121.7, 108.1, 77.7, 22.5, 21.9 ppm.

MS (EI^+) m/z = 274.0, 259.0, 245.0, 231.0, 215.0, 203.0, 178.0, 169.0, 153.0, 141.0, 128.0, 115.0, 105.0, 91.0, 77.0, 65.0, 51.0, 43.0.

HRMS (EI^+) m/z for $C_{20}H_{18}O$ [M^+]: calc.: 274.1352, found: 274.1348.

IR (ATR, neat): $\tilde{\nu}$ = 3060, 3026, 2969, 2932, 2870, 1656, 1597, 1579, 1566, 1493, 1464, 1447, 1383, 1363, 1317, 1259, 1210, 1177, 1159, 1134, 1090, 1074, 1029, 1003, 989, 930, 913, 897, 881, 847, 821, 801, 783, 756, 719, 689, 670, 617, 554 cm^{-1}.

4-Cyclopropyl-1-(4-fluorophenyl)-2-(4-methoxybenzylidene)but-3-yn-1-one (1u)

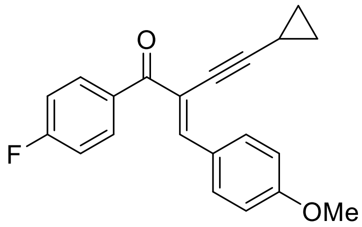

According to the general procedure 1 the title compound was prepared using 2-bromo-1-(4-fluorophenyl)-3-(4-methoxyphenyl)prop-2-en-1-one (1.68 g, 5.0 mmol, 1.0 equiv.) and cyclopropylacetylene (0.523 mL, 6.0 mmol, 1.2 equiv.). The product was obtained as a brown liquid (962 mg, 2.7 mmol, 54%).

R$_f$ = 0.30 (*n*-pentane:diethyl ether = 20:1).

^{1}H NMR (500 MHz, $CDCl_3$): δ = 8.04–7.98 (m, 2H), 7.97–7.91 (m, 2H), 7.45 (s, 1H), 7.14–7.08 (m, 2H), 6.96–6.91 (m, 2H), 3.86 (s, 3H), 1.49 (tt, *J* = 8.3, 5.0 Hz, 1H), 0.92–0.85 (m, 2H), 0.79–0.71 (m, 2H) ppm. **^{13}C NMR** (126 MHz, $CDCl_3$): δ = 192.7, 165.3 (d, *J* = 253.3 Hz), 161.5, 144.3, 134.0 (d, *J* = 3.0 Hz), 132.3 (d, *J* = 9.1 Hz), 132.1, 127.9, 118.8, 115.1 (d, *J* = 21.8 Hz), 114.1, 105.7, 73.9, 55.5, 8.8, 1.0 ppm. **^{19}F NMR** (470 MHz, $CDCl_3$): δ = -106.67 ppm.

MS (EI$^+$) m/z = 320.0, 305.0, 289.0, 277.0, 263.0, 246.0, 233.0, 220.0, 197.0, 182.0, 169.0, 165.0, 153.0, 139.0, 126.0, 123.0, 115.0, 95.0, 77.0, 69.0, 45.0.

HRMS (EI$^+$) m/z for $C_{21}H_{17}O_2F$ [M$^+$]: calc.: 320.1207, found: 320.1217.

IR (ATR, neat): $\tilde{\nu}$ = 3074, 3007, 2934, 2839, 2212, 1656, 1596, 1559, 1504, 1462, 1442, 1423, 1406, 1343, 1306, 1252, 1173, 1154, 1117, 1086, 1053, 1027, 993, 946, 921, 884, 826, 803, 764, 734, 723, 697, 633, 611, 531, 517 cm^{-1}.

7-Chloro-2-(4-methylbenzylidene)-1-phenylhept-3-yn-1-one (1v)

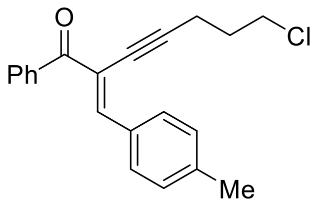

According to the general procedure 1 the title compound was prepared using 2-bromo-1-phenyl-3-(*p*-tolyl)prop-2-en-1-one (1.51 g, 5.0 mmol, 1.0 equiv.) and 5-chloropent-1-yne (0.649 mL, 6.0 mmol, 1.2 equiv.). The product was obtained as a yellow solid (996 mg, 3.3 mmol, 67%).

R*f* = 0.30 (*n*-pentane:diethyl ether = 20:1).

^{1}H NMR (500 MHz, $CDCl_3$): δ = 7.95–7.91 (m, 2H), 7.90–7.86 (m, 2H), 7.57–7.53 (m, 1H), 7.48–7.43 (m, 3H), 7.26–7.21 (m, 2H), 3.58 (t, *J* = 6.3 Hz, 2H), 2.67 (t, *J* = 6.7 Hz, 2H), 2.41 (s, 3H), 2.00 (p, *J* = 6.5 Hz, 2H) ppm. **^{13}C NMR** (126 MHz, $CDCl_3$): δ = 194.4, 145.2, 141.2, 137.7, 132.4, 132.2, 130.3, 129.7, 129.4, 128.2, 120.5, 100.4, 79.3, 43.7, 31.1, 21.8, 17.6 ppm.

MS (EI$^+$) m/z = 322.0, 307.0, 294.0, 273.0, 259.0, 245.0, 231.0, 215.0, 202.0, 189.0, 181.0, 165.0, 153.0, 139.0, 119.0, 105.0, 91.0, 77.0, 65.0, 51.0, 41.0.

HRMS (EI$^+$) m/z for $C_{21}H_{19}ClO$ [M$^+$]: calc.: 322.1124, found: 322.1116.

IR (ATR, neat): $\tilde{\nu}$ = 3026, 2959, 2919, 2216, 1660, 1597, 1579, 1559, 1509, 1446, 1427, 1379, 1353, 1317, 1290, 1259, 1210, 1183, 1157, 1120, 1036, 1020, 1001, 986, 970, 916, 871, 851, 836, 814, 794, 747, 720, 710, 693, 661, 633, 617, 541, 521 cm^{-1}.

2-(4-Methylbenzylidene)-1-phenyloct-3-yn-1-one (1w)

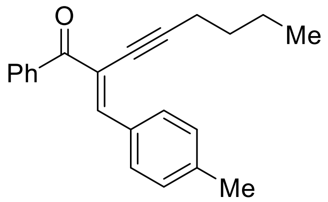

According to the general procedure 1 the title compound was prepared using 2-bromo-1-phenyl-3-(*p*-tolyl)prop-2-en-1-one (1.51 g, 5.0 mmol, 1.0 equiv.) and 5-chloropent-1-yne (0.710 mL, 6.0 mmol, 1.2 equiv.). The product was obtained as a yellow solid (1.02 g, 3.3 mmol, 68%).

$\mathbf{R}_f$ = 0.73 (*n*-pentane:diethyl ether = 20:1).

^{1}**H NMR** (500 MHz, $CDCl_3$): δ = 7.99–7.94 (m, 2H), 7.93–7.87 (m, 2H), 7.58–7.52 (m, 1H), 7.48–7.41 (m, 3H), 7.23–7.22 (m, 2H), 2.47 (t, *J* = 7.0 Hz, 2H), 2.40 (s, 3H), 1.59–1.52 (m, 2H), 1.45–1.36 (m, 2H), 0.90 (t, *J* = 7.4 Hz, 3H) ppm. ^{13}**C NMR** (126 MHz, $CDCl_3$): δ = 194.6, 144.5, 140.9, 137.6, 132.34, 132.26, 130.2, 129.8, 129.3, 128.1, 120.9, 103.0, 78.3, 30.4, 22.1, 21.8, 19.9, 13.7 ppm.

MS: (EI^+) m/z = 302.0, 287.0, 273.0, 259.0, 245.0, 231.0, 221.0, 215.0, 191.0, 181.0, 165.0, 155.0, 143.0, 129.0, 115.0, 105.0, 91.0, 77.0, 65.0, 51.0.

HRMS (EI^+) m/z for $C_{22}H_{22}O$ [M^+]: calc.: 302.1665, found: 302.1668.

IR (ATR, neat): $\tilde{\nu}$ = 3026, 2957, 2929, 2216, 1660, 1596, 1579, 1559, 1509, 1447, 1427, 1413, 1389, 1319, 1246, 1210, 1177, 1106, 1069, 1034, 1019, 1001, 973, 953, 903, 860, 814, 796, 759, 747, 717, 704, 693, 660, 643, 610, 551, 520 cm^{-1}.

3 $InBr_3$-catalysed Transfer-Hydrogenation for the Synthesis of Furans

General Procedure 2

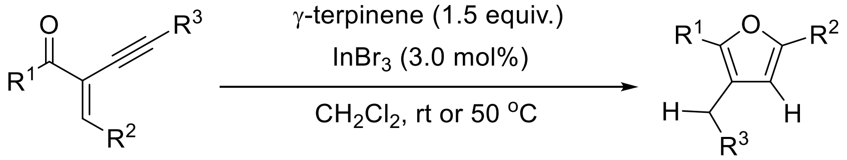

A sealed tube was charged with indium tribromide (10.6 mg, 0.03 mmol, 3.0 mol%) and a stir bar. The sealed tube was evacuated and heated to 100 °C for 30 min.[a] Thereafter, the sealed tube was charged with N_2. Then 5.0 mL CH_2Cl_2, γ-terpinene (240 μL, 1.50 mmol, 1.5 equiv.) and the alkynyl enone (1 mmol) were added in sequence. The mixture was stirred at rt or heated to 50 °C until the reaction was complete (monitored by TLC or GC-MS). Then the reaction mixture was purified by flash column chromatography (SiO_2, *n*-pentane → *n*-pentane:diethyl ether = 20:1) to afford the pure furan product.

a. The $InBr_3$ was not stored in a glove box, so it should be pre-dried before the reaction.

3-Benzyl-2,5-diphenylfuran (3a)

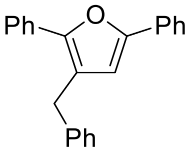

According to the general procedure 2 the title compound was prepared using 2-benzylidene-1,4-diphenylbut-3-yn-1-one (308 mg, 1.0 mmol, 1.0 equiv.) at rt. The product was obtained as a colourless solid (304 mg, 0.98 mmol, 98%). Gram-scale Procedure: A Schlenk flask was charged with indium tribromide (35.5 mg, 0.1 mmol, 3.0 mol%) and a stir bar. The sealed tube was evacuated and heated to 100 °C for 30 min. Thereafter, the sealed tube was charged with N_2. Then 17.0 mL CH_2Cl_2, γ-terpinene (1.4 mL, 1.50 mmol, 1.5 equiv.) and the 2-benzylidene-1,4-diphenylbut-3-yn-1-one (1.05 g, 3.4 mmol, 1.0 equiv.) were added in sequence. The mixture was stirred at rt until the reaction was complete (monitored by TLC or GC-MS). Then the reaction mixture was purified by flash column chromatography (SiO_2, *n*-pentane → *n*-pentane:diethyl ether = 20:1) to afford the title compound (1.03 g, 3.3 mmol, 98%).

R$_f$ = 0.59 (*n*-pentane:diethyl ether = 40:1).

^{1}H NMR (500 MHz, $CDCl_3$): δ = 7.71–7.65 (m, 4H), 7.43–7.34 (m, 4H), 7.33–7.19 (m, 7H), 6.52 (s, 1H), 4.07 (s, 2H, CH_2) ppm. **^{13}C NMR** (125 MHz, $CDCl_3$): δ = 152.7, 149.3, 140.3, 131.8, 131.1, 129.1, 129.0, 128.9, 127.7, 127.6, 126.7, 126.1, 124.2, 122.2, 110.2, 32.5 ppm. The analytical data are in accordance with the literature.[7]

3-Benzyl-2-(4-methoxyphenyl)-5-phenylfuran (3b)

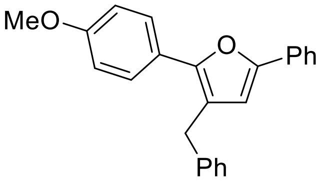

According to the general procedure 2 the title compound was prepared using 2-benzylidene-1-(4-methoxyphenyl)-4-phenyl-but-3-yn-1-one (338 mg, 1.0 mmol, 1.0 equiv.) at rt. The product was obtained as a light yellow solid (323 mg, 0.95 mmol, 95%).

Mp: 103-104 °C.

R$_f$ = 0.31 (*n*-pentane:diethyl ether = 40:1).

^{1}H NMR (500 MHz, $CDCl_3$): δ = 7.73–7.65 (m, 2H), 7.65–7.58 (m, 2H), 7.39–7.29 (m, 4H), 7.29–7.19 (m, 4H), 6.99–6.91 (m, 2H), 6.52 (s, 1H), 4.04 (s, 2H), 3.84 (s, 3H) ppm. **^{13}C NMR** (126 MHz, $CDCl_3$): δ = 159.4, 151.9, 149.2, 140.3, 130.9, 128.8, 128.72, 128.65, 127.3, 127.2, 126.4, 124.4, 123.7, 120.4, 114.3, 109.8, 55.5, 32.2 ppm.

MS: (EI^+) m/z = 340.2, 325.1, 309.1, 278.1, 263.0, 235.1, 220.0, 202.0, 189.0, 178.0, 170.1, 165.0, 152.1, 135.1, 131.2, 127.1, 105.0, 91.0, 77.0, 65.0, 51.0.

HRMS: (EI^+) m/z for $C_{24}H_{20}O$ [M^+]: calc.: 340.1458, found: 340.1457.

IR (ATR, neat): $\tilde{\nu}$ = 2957, 2833, 1606, 1572, 1504, 1486, 1452, 1442, 1303, 1244, 1174, 1107, 1072, 1059, 931, 906, 833, 757, 724, 686, 667, 620, 607, 577, 521 cm^{-1}.

3-Benzyl-2-(4-fluorophenyl)-5-phenylfuran (3c)

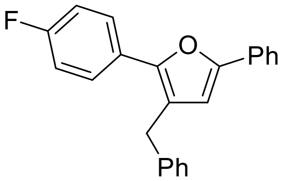

According to the general procedure 2 the title compound was prepared using 2-benzylidene-1-(4-fluorophenyl)-4-phenylbut-3-yn-1-one (328 mg, 1.0 mmol, 1.0 equiv.) at 50 °C. The product was obtained as a light yellow solid (293 mg, 0.89 mmol, 89%).

Mp: 85-86 °C

R$_f$ = 0.58 (*n*-pentane:diethyl ether = 40:1).

^{1}H NMR (500 MHz, $CDCl_3$): δ = 7.69–7.65 (m, 2H), 7.65–7.60 (m, 2H), 7.38–7.34 (m, 2H), 7.33–7.28 (m, 2H), 7.26–7.20 (m, 4H), 7.11–7.06 (m, 2H), 6.51 (s, 1H), 4.03 (s, 2H) ppm. **^{13}C NMR** (126 MHz, $CDCl_3$): δ = 162.1 (d, J = 247.5 Hz), 152.5, 148.3, 139.9, 130.7, 128.8 (d, J = 3.7 Hz), 128.6, 127.6, 127.5 (d, J = 4.7 Hz), 126.5, 123.9, 121.5, 115.8 (d, J = 21.7 Hz), 109.9, 32.2 ppm. **^{19}F NMR** (470 MHz, $CDCl_3$): δ = -114.22 ppm.

MS: (EI^+) m/z = 328.1, 209.0, 283.3, 270.0, 251.0, 233.0, 221.0, 202.0, 183.0, 164.1, 135.2, 127.1, 123.1, 116.2, 105.1, 95.0, 91.0, 77.0, 65.0, 51.0.

HRMS: (EI^+) m/z for $C_{23}H_{17}OF$ [M^+]: calc.: 328.1258, found: 328.1258.

IR (ATR, neat): $\tilde{\nu}$ = 3062, 3027, 2959, 1602, 1590, 1577, 1549, 1502, 1494, 1483, 1450, 1437, 1323, 1230, 1219, 1157, 1124, 1096, 1070, 1059, 1027, 970, 929, 907, 833, 817, 776, 756, 731, 719, 696, 690, 683, 666, 636, 619, 604, 579, 553, 510 cm^{-1}.

2-(4-Fluorophenyl)-3-(4-methoxybenzyl)-5-phenylfuran (3d)

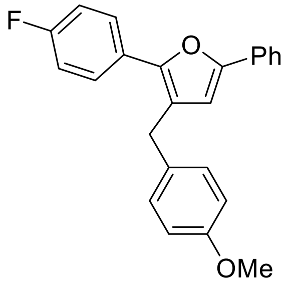

According to the general procedure 2 the title compound was prepared using 1-(4-fluorophenyl)-2-(4-methoxybenzylidene)-4-phenylbut-3-yn-1-one (356 mg, 1.0 mmol, 1.0 equiv.) at rt. The product was obtained as a light yellow liquid solid (358 mg, 1.0 mmol, 99%).

R_f = 0.38 (*n*-pentane:diethyl ether = 40:1).

^{1}H NMR (500 MHz, $CDCl_3$): δ = 7.70–7.66 (m, 2H), 7.66–7.61 (m, 2H), 7.38–7.25 (m, 2H), 7.26–7.21 (m, 1H), 7.19–7.15 (m, 2H), 7.13–7.07 (m, 2H), 6.89–6.83 (m, 2H), 6.51 (s, 1H), 3.97 (s, 2H), 3.79 (s, 3H) ppm. **^{13}C NMR** (126 MHz, $CDCl_3$): δ = 162.1 (d, J = 247.4 Hz), 158.4 152.4, 148.1, 131.9, 130.8, 129.5, 128.8, 127.8 (d, J = 3.2 Hz), 127.6, 127.5 (d, J = 1.7 Hz), 123.9, 122.0 115.8 (d, J = 21.7 Hz), 114.2, 109.9, 55.4, 31.3 ppm. **^{19}F NMR** (470 MHz, $CDCl_3$): δ = -114.27 ppm.

MS: (EI^+) m/z = 358.0, 343.0, 237.0, 297.0, 281.0, 266.0, 253.0, 238.0, 221.0, 209.0, 179.1, 147.0, 135.0, 123.0, 105.0, 95.0, 77.0, 69.0, 55.0, 43.0.

HRMS: (EI^+) m/z for $C_{24}H_{19}O_2F$ [M^+]: calc.: 358.1364, found: 358. 1358.

IR (ATR, neat): $\tilde{\nu}$ = 2834, 1604, 1549, 1510, 1502, 1484, 1463, 1440, 1302, 1176, 1157, 1094, 1072, 1056, 1034, 931, 99, 834, 811, 757, 717, 690, 667, 619, 600, 540, 511 cm^{-1}.

3-(4-Methoxybenzyl)-2,5-diphenylfuran (3e)

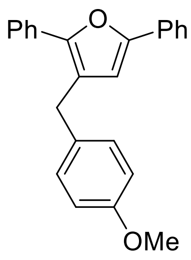

According to the general procedure 2 the title compound was prepared using 2-(4-methoxybenzylidene)-1,4-diphenylbut-3-yn-1-one (338 mg, 1.0 mmol, 1.0 equiv.) at rt. The product was obtained as a colourless solid (340 mg, 1.0 mmol, 99%).

R*f* = 0.38 (*n*-pentane:diethyl ether = 40:1).

^{1}H NMR (500 MHz, $CDCl_3$): δ = 7.77–7.61 (m, 4H), 7.46–7.33 (m, 4H), 7.32–7.22 (m, 2H), 7.22–7.15 (m, 2H), 6.93–6.80 (m, 2H), 6.51 (s, 1H), 4.02 (s, 2H), 3.79 (s, 3H) ppm. **^{13}C NMR** (126 MHz, $CDCl_3$): δ = 158.3, 152.3, 148.8, 132.1, 131.5, 130.8, 129.6, 128.8, 127.4, 127.2, 125.8, 123.9, 122.4, 114.1, 109.9, 55.4, 31.4 ppm.

HRMS: (EI^+) m/z for $C_{24}H_{20}O$ [M^+]: calc.: 340.1458, found: 340.1462.

The analytical data are in accordance with the literature.[7]

3-(4-Methylbenzyl)-2,5-diphenylfuran (3f)

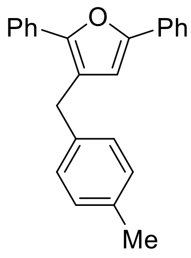

According to the general procedure 2 the title compound was prepared using 2-(4-methylbenzylidene)-1,4-diphenylbut-3-yn-1-one (322 mg, 1.0 mmol, 1.0 equiv.) at rt. The product was obtained as a light yellow solid (301 mg, 0.93 mmol, 93%).

Mp: 104-105 °C.

R*f* = 0.58 (*n*-pentane:diethyl ether = 40:1).

^{1}H NMR (500 MHz, $CDCl_3$): δ = 7.11–6.98 (m, 4H), 7.35–7.25 (m, 4H), 7.22–7.13 (m, 2H), 7.11–6.98 (m, 4H), 6.43 (s, 1H), 3.95 (s, 2H), 2.25 (s, 3H) ppm. **^{13}C NMR** (126 MHz, $CDCl_3$): δ = 152.4, 148.9, 137.0, 135.9, 131.6, 130.9, 129.4, 128.8, 128.6, 127.4, 127.3, 125.8, 123.9, 122.2, 110.0, 31.8, 21.2 ppm.

MS (EI^+) m/z = 324.1, 309.1, 291.1, 279.1, 265.1, 247.1, 233.0, 219.1, 204.1, 189.1, 178.0, 139.2, 127.1, 123.2, 115.1, 105.0, 91.0, 77.0, 65.0, 51.0.

HRMS: (EI^+) m/z for $C_{24}H_{20}O$ [M^+]: calc.: 324.1509, found: 324.1508.

IR (ATR, neat): $\tilde{\nu}$ = 3017, 2953, 1609, 1592, 1513, 1493, 1480, 1443, 1432, 1330, 1309, 1197, 1156, 1120, 1109, 1070, 1054, 1033, 1024, 1004, 963, 931, 910, 851, 811, 760, 690, 636, 584, 514 cm^{-1}.

3-(4-Chlorobenzyl)-2,5-diphenylfuran (3g)

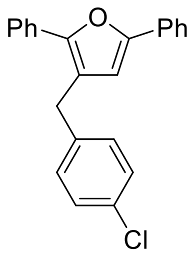

According to the general procedure 2 the title compound was prepared using 2-(4-chlorobenzylidene)-1,4-diphenylbut-3-yn-1-one (342 mg, 1.0 mmol, 1.0 equiv.) at 50 °C. The product was obtained as a brown solid (344 mg, 1.0 mmol, 99%).

Mp: 107-108 °C.

$\mathbf{R}_f$ = 0.57 (*n*-pentane:diethyl ether = 40:1).

^{1}H NMR (500 MHz, $CDCl_3$): δ = 7.75–7.56 (m, 4H), 7.37 (dt, *J* = 18.2, 7.7 Hz, 4H), 7.30–7.20 (m, 4H), 7.17–7.16 (m, *J* = 8.3 Hz, 2H), 6.47 (s, 1H), 4.01 (s, 2H) ppm. **^{13}C NMR** (126 MHz, $CDCl_3$): δ = 152.6, 149.1, 138.6, 132.3, 131.3, 130.7, 130.0, 128.9, 128.8, 127.6, 127.5, 125.8, 123.9, 121.4, 109.7, 31.7 ppm.

MS: (EI^+) m/z = 344.1, 309.1, 291.1, 268.2, 242.2, 231.2, 211.2, 202.1, 165.0, 156.0, 139.0, 122.0, 111.0, 105.0, 83.9, 77.0, 51.0.

HRMS: (EI^+) m/z for $C_{23}H_{17}OCl$ [M^+]: calc.: 344.0962, found: 344.0958.

IR (ATR, neat): $\tilde{\nu}$ = 3062, 1663, 1594, 1490, 1449, 1363, 1317, 1262, 1219, 1176, 1090, 1013, 1001, 961, 931, 820, 757, 687, 667, 647, 616, 519, 506 cm^{-1}.

2,5-Diphenyl-3-(thiophen-2-ylmethyl)furan (3h)

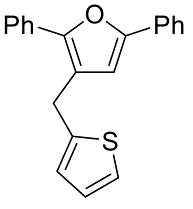

According to the general procedure 2 the title compound was prepared using 1,4-diphenyl-2-(thiophen-2-ylmethylene)but-3-yn-1-one (314 mg, 1.0 mmol, 1.0 equiv.) at rt. The product was obtained as a brown liquid (312 mg, 0.99

mmol, 99%).

R*f* = 0.59 (*n*-pentane:diethyl ether = 40:1).

^{1}H NMR (500 MHz, $CDCl_3$): δ = 7.72–7.65 (m, 4H), 7.43–7.34 (m, 4H), 7.31–7.21 (m, 2H), 7.15 (dd, *J* = 5.1, 1.2 Hz, 1H), 6.93 (dd, *J* = 5.1, 3.4 Hz, 1H), 6.87 (dt, *J* = 3.4, 1.2 Hz, 1H), 6.63 (s, 1H), 4.21 (s, 2H) ppm. **^{13}C NMR** (126 MHz, $CDCl_3$): δ = 152.6, 149.0, 143.3, 131.3, 130.8, 128.83, 128.81, 127.6, 127.5, 127.1, 126.0, 125.2, 124.0, 123.9, 121.6, 109.6, 26.9 ppm.

MS: (EI^+) m/z = 316.1, 297.1, 281.1, 253.2, 239.2, 211.2, 202.1, 178.1, 165.1, 152.1, 127.1, 115.1, 105.0, 97.0, 97.0, 84.0, 77.0, 63.0, 51.0, 43.0.

HRMS: (EI^+) m/z for $C_{21}H_{16}OS$ [M^+]: calc.: 316.0916, found: 316.0915.

IR (ATR, neat): $\tilde{\nu}$ = 3059, 2956, 1594, 1493, 1482, 1444, 1383, 1229, 1200, 1180, 1157, 1106, 1072, 1054, 1026, 1006, 991, 933, 910, 850, 814, 757, 687, 553 cm^{-1}.

3-Benzyl-2-phenyl-5-(4-(trifluoromethyl)phenyl)furan (3i)

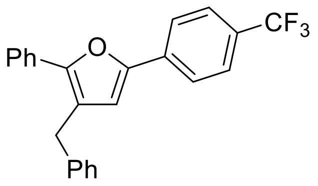

According to the general procedure 2 the title, compound was prepared using 2-benzylidene-1-phenyl-4-(4-(trifluoromethyl)-phenyl)but-3-yn-1-one (376 mg, 1.0 mmol, 1.0 equiv.) at 50 °C. The product was obtained as a light yellow liquid (338 mg, 0.89 mmol, 89%).

R*f* = 0.68 (*n*-pentane:diethyl ether = 40:1).

^{1}H NMR (500 MHz, $CDCl_3$): δ = 7.80–7.77 (m, 2H), 7.73–7.69 (m, 2H), 7.64–7.60 (m, 2H), 7.47–7.42 (m, 2H), 7.36–7.32 (m, 3H), 7.29–7.24 (m, 3H), 6.64 (s, 1H), 4.10 (s, 2H) ppm. **^{13}C NMR** (126 MHz, $CDCl_3$): δ = 150.9, 150.2, 139.8, 133.9, 131.1, 128.9, 128.8, 128.7, 127.8, 126.6, 126.1, 125.8 (q, *J* = 3.7 Hz), 123.8, 122.3, 111.8, 32.2 ppm. The signal for the CF_3 carbon was not detectable. **^{19}F NMR** (470 MHz, $CDCl_3$): δ = -62.51 ppm.

MS: (EI^+) m/z = 378.0, 359.0, 341.0, 316.0, 301.0, 259.0, 233.0, 221.1, 205.1, 190.0, 173.0, 145.0, 127.0, 105.0, 91.0, 77.0, 57.0.

HRMS: (EI^+) m/z for $C_{24}H_{17}OF_3$ [M^+]: calc.: 378.1226, found: 378.1228.

IR (ATR, neat): $\tilde{\nu}$ = 3027, 2959, 2926, 1617, 1600, 1487, 1453, 1446, 1417, 1320, 1260, 1217, 1164, 1120, 1107, 1067, 1051, 1030, 1014, 934, 911, 843, 829, 760, 727, 692, 669, 594 cm^{-1}.

3-Benzyl-5-(4-bromophenyl)-2-phenylfuran (3j)

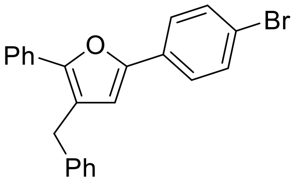

According to the general procedure 2 the title compound was prepared using 2-benzylidene-4-(4-bromophenyl)-1-phenylbut-3-yn-1-one (387 mg, 1.0 mmol, 1.0 equiv.) at 50 °C. The product was obtained as a colourless solid (387 mg, 0.99 mmol, 99%).

R*f* = 0.69 (*n*-pentane:diethyl ether = 40:1).

^{1}H NMR (500 MHz, $CDCl_3$): δ = 7.68–7.64 (m, 2H), 7.54–7.50 (m, 2H), 7.48–7.45 (m, 2H), 7.42–7.37 (m, 2H), 7.32–7.26 (m, 3H), 7.24–7.20 (m, 3H), 6.49 (s, 1H), 4.05 (s, 2H) ppm. **^{13}C NMR** (126 MHz, $CDCl_3$): δ = 151.4, 149.4, 139.9, 131.9, 131.3, 129.8, 128.84, 128.78, 128.69, 127.5, 126.5, 125.9, 125.4, 122.1, 121.2, 110.5, 32.2 ppm.

MS (EI^+) m/z =390.1, 371.1, 339.2, 326.9, 313.9, 291.1, 279.1, 265.0, 221.0, 203.0, 182.9, 155.0, 127.1, 115.3, 105.0, 91.0, 77.0, 65.0, 41.0.

HRMS: (EI^+) m/z for $C_{24}H_{17}OBr$ [M^+]: calc.: 388.0457, found: 388.0455.

IR (ATR, neat): $\tilde{\nu}$ = 3060, 3026, 2957, 1600, 1542, 1492, 1476, 1453, 1407, 1383, 1307, 1262, 1216, 1177, 1100, 1072, 1051, 1030, 1007, 931, 911, 819, 759, 730, 691, 667 cm^{-1}.

3-Benzyl-2-phenyl-5-(*p*-tolyl)furan (3k)

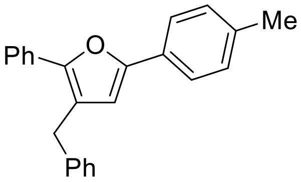

According to the general procedure 2 the title compound was prepared using 2-benzylidene-1-phenyl-4-(*p*-tolyl)but-3-yn-1-one (322 mg, 1.0 mmol, 1.0 equiv.) at rt. The product was obtained as a light yellow solid (256 mg, 0.79 mmol, 79%).

R*f* = 0.64 (*n*-pentane:diethyl ether = 40:1).

^{1}H NMR (500 MHz, $CDCl_3$): δ = 7.72–7.64 (m, 2H), 7.61–7.55 (m, 2H), 7.41–7.37 (m, 2H), 7.33–7.24 (m, 5H), 7.24–7.19 (m, 1H), 7.18–7.15 (m, 2H), 6.45 (s, 1H), 4.06 (s, 2H), 2.34 (s, 3H) ppm. **^{13}C NMR** (126 MHz, $CDCl_3$): δ = 152.7, 148.6, 140.2, 137.3, 131.6, 129.5, 128.8, 128.72, 128.71, 128.2, 127.2, 126.4, 125.8, 123.9, 121.9, 109.2, 32.3, 21.4 ppm.

MS: (EI^+) m/z = 324.1, 305.1, 280.1, 247.1, 231.0, 219.1, 204.1, 178.0, 141.2, 127.1, 123.2, 119.1, 105.0, 91.0, 77.0, 65.0, 51.0, 43.0.

HRMS: (EI^+) m/z for $C_{24}H_{20}O$ [M^+]: calc.: 324.1509, found: 324.1513.

IR (ATR, neat): $\tilde{\nu}$ = 3026, 2956, 2922, 1599, 1500, 1493, 1487, 1452, 1377, 1316, 1260, 1214, 1182, 1157, 1073, 1053, 1029, 929, 906, 813, 761, 724, 714, 689, 669, 504 cm^{-1}.

3-Benzyl-2-phenyl-5-(*m*-tolyl)furan (3l)

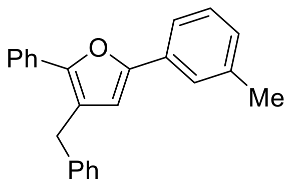

According to the general procedure 2 the title compound was prepared using 2-benzylidene-1-phenyl-4-(*m*-tolyl)but-3-yn-1-one (322 mg, 1.0 mmol, 1.0 equiv.) at rt. The product was obtained as a light yellow liquid (269 mg, 0.83 mmol, 83%).

R*f* = 0.68 (*n*-pentane:diethyl ether = 40:1).

^{1}H NMR (500 MHz, $CDCl_3$): δ = 7.74–7.69 (m, 2H), 7.55–7.51 (m, 2H), 7.45–7.42 (m, 2H), 7.37–7.31 (m, 3H), 7.31–7.29 (m, 3H), 7.28–7.25 (m, 1H), 7.11–7.07 (m, 1H), 6.53 (s, 1H), 4.10 (s, 2H), 2.40 (s, 3H) ppm. **^{13}C NMR** (126 MHz, $CDCl_3$): δ = 152.6, 148.9, 140.1, 138.4, 131.6, 130.8, 128.8, 128.73, 128.71, 128.3, 127.3, 126.4, 125.8, 124.5, 121.9, 121.1, 109.9, 32.3, 21.6 ppm.

MS: (EI^+) m/z = 324.1, 305.1, 279.1, 247.1, 231.0, 219.1, 204.1, 178.0, 165.0, 161.0, 141.0, 127.0, 123.1, 119.0, 105.0, 91.0, 77.0, 65.0, 51.0.

HRMS: (EI^+) m/z for $C_{24}H_{20}O$ [M^+]: calc.: 324.1509, found: 324.1500.

IR (ATR, neat): $\tilde{\nu}$ = 3026, 2960, 2920, 1599, 1492, 1380, 1317, 1260, 1216, 1092, 1073, 1060, 1029, 951, 906, 876, 840, 783, 760, 724, 691, 523 cm^{-1}.

3-Benzyl-5-(4-methoxyphenyl)-2-phenylfuran (3m)

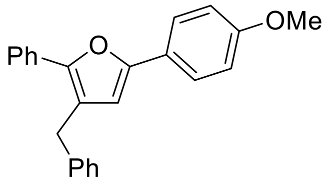

According to the general procedure 2 the title compound was prepared using 2-benzylidene-4-(4-methoxyphenyl)-1-phenyl-but-3-yn-1-one (338 mg, 1.0 mmol, 1.0 equiv.) at rt. The product was obtained as a light yellow solid (136 mg, 0.40 mmol, 40%).

Mp: 131-133 °C.

R*f* = 0.34 (*n*-pentane:diethyl ether = 40:1).

^{1}H NMR (500 MHz, $CDCl_3$): δ = 7.72–7.67 (m, 2H), 7.67–7.63 (m, 2H), 7.45–7.40 (m, 2H), 7.36–7.31 (m, 2H), 7.31–7.27 (m, 3H), 7.27–7.23 (m, 1H), 6.96–6.91 (m, 2H), 6.41 (s, 1H),

4.09 (s, 2H), 3.84 (s, 3H) ppm. **^{13}C NMR** (126 MHz, $CDCl_3$): δ = 159.3, 152.6, 148.4, 140.2, 131.6, 128.8, 128.7, 127.1, 126.4, 125.7, 125.4, 124.0, 121.9, 114.3, 108.4, 55.5, 32.3 ppm.

MS: (EI^+) m/z = 340.2, 325.0, 311.1, 287.1, 279.1, 263.0, 235.1, 221.0, 202.0, 189.0, 165.0, 152.1, 135.1, 131.2, 127.1, 105.0, 91.0, 77.0, 65.0, 51.0.

HRMS: (EI^+) m/z for $C_{24}H_{20}O$ [M^+]: calc.: 340.1458, found: 340.1453.

IR (ATR, neat): $\tilde{\nu}$ = 2833, 1602, 1583, 1599, 1456, 1439, 1422, 1300, 1246, 1184, 1172, 1106, 1073, 1053, 1027, 970, 934, 830, 813, 804, 764, 727, 696, 669, 657, 619, 596, 540, 520 cm^{-1}.

3-Benzyl-2-methyl-5-phenylfuran (3n)

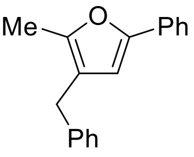

According to the general procedure 2 the title compound was prepared using 3-benzylidene-5-phenylpent-4-yn-2-one (246 mg, 1.0 mmol, 1.0 equiv.) at rt. The product was obtained as a colourless solid (231 mg, 0.93 mmol, 93%).

R*f* = 0.36 (*n*-pentane).

^{1}H NMR (500 MHz, $CDCl_3$): δ = 7.61–7.57 (m, 2H,), 7.36–7.28 (m, 4H), 7.24–7.18 (m, 4H), 6.41 (s, 1H), 3.74 (s, 2H), 2.34 (s, 3H) ppm. **^{13}C NMR** (125 MHz, $CDCl_3$): δ = 151.4, 147.7, 140.7, 131.3, 128.5, 128.4, 128.3, 126.7, 126.1, 123.2, 120.0, 107.6, 31.2, 11.7 ppm.

The analytical data are in accordance with the literature.[8]

3-Benzyl-5-(4-methoxyphenyl)-2-methylfuran (3o)

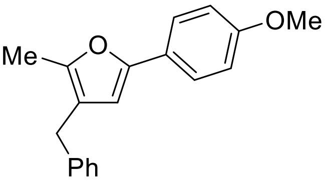

According to the general procedure 2 the title compound was prepared using 3-benzylidene-5-(4-methoxyphenyl)pent-4-yn-2-one (276 mg, 1.0 mmol, 1.0 equiv.) at rt. The product was obtained as a colourless solid (54.5 mg, 0.20 mmol, 20%).

Mp: 59-60 °C.

R*f* = 0.11 (*n*-pentane).

^{1}H NMR (500 MHz, $CDCl_3$): δ = 7.55–7.50 (m, 2H), 7.32–7.28 (m, 2H), 7.24–7.19 (m, 3H), 6.90–6.86 (m, 2H), 6.28 (s, 1H), 3.82 (s, 3H), 3.73 (s, 2H), 2.32 (s, 3H) ppm. **^{13}C NMR** (125 MHz, $CDCl_3$): δ = 158.8, 151.6, 147.1, 141.0, 128.6, 128.5, 126.2, 124.8, 124.5, 120.0, 114.2, 106.2, 55.4, 31.4, 11.8 ppm.

MS: (EI^+) m/z = 278.1, 263.1, 245.2, 235.2, 220.2, 202.1, 185.1, 178.1, 165.1, 157.1, 135.1, 128.1, 115.1, 102.0, 91.0, 77.0, 65.0, 51.0.

HRMS: (EI^+) m/z for $C_{19}H_{18}O_2$ [M^+]: calc.: 278.1301, found: 278.1306.

IR (ATR, neat): $\tilde{\nu}$ = 3029, 3003, 2962, 2947, 2916, 2840, 1633, 1610, 1583, 1557, 1499, 1493, 1469, 1462, 1452, 1443, 1317, 1303, 1276, 1243, 1200, 1176, 1113, 1103, 1054, 1027, 961, 930, 837, 833, 821, 813, 801, 794, 774, 731, 700, 670, 657, 639, 630, 616, 599, 567, 521 cm^{-1}.

3-(2-Chlorobenzyl)-2-methyl-5-phenylfuran (3p)

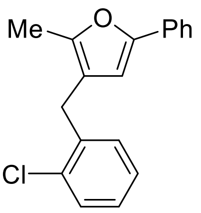

According to the general procedure 2 the title compound was prepared using 3-(2-chlorobenzylidene)-5-phenylpent-4-yn-2-one (280 mg, 1.0 mmol, 1.0 equiv.) at 50 °C. The product was obtained as a brown liquid (275 mg, 0.97 mmol, 97%).

R_f = 0.78 (*n*-pentane:diethyl ether = 40:1).

^{1}H NMR (500 MHz, $CDCl_3$): δ = 7.60–7.55 (m, 2H), 7.35 (dd, *J* = 7.4, 1.6 Hz, 1H), 7.33–7.28 (m, 2H), 7.20–7.10 (m, 4H), 6.43 (s, 1H), 3.81 (s, 2H), 2.32 (s, 3H) ppm. **^{13}C NMR** (126 MHz, $CDCl_3$): δ = 151.6, 148.3, 138.4, 134.1, 131.2, 130.3, 129.6, 128.7, 127.7, 127.0, 126.9, 123.4, 118.6, 107.7, 29.0, 12.0 ppm.

MS: (EI^+) m/z = 282.0, 263.0, 247.0, 229.0, 215.0, 202.0, 189.0, 171.0, 139.1, 127.1, 115.1, 105.0, 89.0, 77.0, 63.0, 51.0, 43.0.

HRMS: (EI^+) m/z for $C_{18}H_{15}OCl$ [M^+]: calc.: 282.0806, found: 282.0802.

IR (ATR, neat): $\tilde{\nu}$ = 3060, 2917, 1672, 1597, 1553, 1487, 1472, 1374, 1347, 1327, 1256, 1213, 1159, 1127, 1103, 1072, 1051, 1037, 1001, 971, 930, 909, 886, 864, 810, 790, 747, 719, 689, 660, 626, 616, 556 cm^{-1}.

3-(4-Chlorobenzyl)-2-methyl-5-phenylfuran (3q)

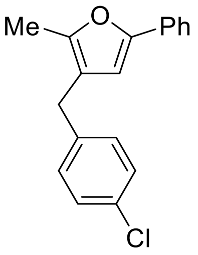

According to the general procedure 2 the title compound was prepared using 3-(4-chlorobenzylidene)-5-phenylpent-4-yn-2-one (280 mg, 1.0 mmol, 1.0 equiv.) at 50 °C. The product was obtained as a colourless solid (282 mg, 1.0 mmol, 99%).

R$_f$ = 0.42 (*n*-pentane).

^{1}H NMR (500 MHz, $CDCl_3$): δ = 7.62–7.53 (m, 2H), 7.34–7.30 (m, 2H), 7.27–7.21 (m, 2H), 7.21–7.17 (m, 1H), 7.15–7.09 (m, 2H), 6.36 (s, 1H), 3.68 (s, 2H), 2.31 (s, 3H) ppm. **^{13}C NMR** (126 MHz, $CDCl_3$): δ = 151.6, 148.0, 139.3, 132.0, 131.1, 129.9, 128.71, 128.68, 126.9, 123.4, 119.7, 107.5, 30.7, 11.9 ppm.

MS: (EI^+) m/z = 282.1, 267.1, 247.1, 229.1, 217.0, 204.0, 189.0, 177.0, 171.0, 157.0, 147.0, 141.0, 125.0, 115.0, 105.0, 84.0, 77.0, 69.0, 49.0, 43.0.

HRMS: (EI^+) m/z for $C_{18}H_{15}OCl$ [M^+]: calc.: 282.0806, found: 282.0802.

IR (ATR, neat): $\tilde{\nu}$ = 3060, 3030, 2956, 2915, 1852, 1600, 1553, 1489, 1447, 1433, 1409, 1327, 1312, 1290, 1276, 1202, 1176, 1110, 1089, 1072, 1054, 1026, 1014, 963, 930, 909, 844, 803, 756, 690, 661, 646, 634, 610 cm^{-1}.

5-((2-Methyl-5-phenylfuran-3-yl)methyl)benzo[*d*][1,3]dioxole (3r)

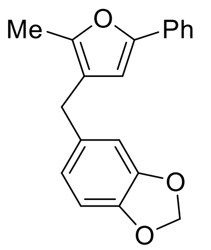

According to the general procedure 2 the title compound was prepared using 3-(benzo[*d*][1,3]dioxol-5-ylmethylene)-5-phenylpent-4-yn-2-one (290 mg, 1.0 mmol, 1.0 equiv.) at rt. The product was obtained as a brown liquid (240 mg, 0.82 mmol, 82%).

R$_f$ = 0.42 (*n*-pentane:diethyl ether = 40:1).

^{1}H NMR (500 MHz, $CDCl_3$): δ = 7.67–7.53 (m, 2H), 7.38–7.30 (m, 2H), 7.24–7.16 (m, 1H), 6.86–6.57 (m, 3H), 6.40 (s, 1H), 5.92 (s, 2H), 3.65 (s, 2H), 2.33 (s, 3H) ppm. **^{13}C NMR** (126 MHz, $CDCl_3$): δ = 151.6, 147.9, 147.8, 146.0, 134.8, 131.2, 128.7, 126.8, 123.4, 121.3, 120.4, 109.1, 108.3, 107.6, 101.0, 31.1, 11.9 ppm.

MS: (EI^+) m/z = 292.1, 277.1, 262.2, 247.2, 233.2, 219.2, 203.1, 189.1, 170.1, 157.1, 129.1, 115.1, 105.0, 77.0, 63.0, 51.0, 43.0.

HRMS: (EI^+) m/z for $C_{19}H_{16}O_3$ [M^+]: calc.: 292.1094, found: 292.1096.

IR (ATR, neat): $\tilde{\nu}$ = 2896, 2774, 1600, 1553, 1502, 1486, 1442, 1359, 1292, 1244, 1230, 1184, 1117, 1092, 1072, 1037, 963, 941, 930, 860, 803, 757, 714, 690, 660, 651, 629, 617, 584 cm^{-1}.

3-Benzyl-5-(4-fluorophenyl)-2-methylfuran (3s)

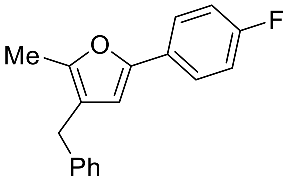

According to the general procedure 2 the title compound was prepared using 3-benzylidene-5-(4-fluorophenyl)pent-4-yn-2-one (264 mg, 1.0 mmol, 1.0 equiv.) at rt or 50 °C. The product was obtained as a light yellow liquid (rt: 168 mg, 0.63 mmol, 63%; 50 °C: 108 mg, 0.41 mmol, 41%).

R_f = 0.78 (*n*-pentane:diethyl ether = 40:1).

^{1}H NMR (500 MHz, $CDCl_3$): δ = 7.60–7.53 (m, 2H), 7.33–7.30 (m, 2H), 7.25–7.19 (m, 3H), 7.06–7.00 (m, 2H), 6.34 (s, 1H), 3.74 (s, 2H), 2.34 (s, 3H) ppm. **^{13}C NMR** (126 MHz, $CDCl_3$): δ = 161.9 (d, *J* = 246.1 Hz), 150.7, 147.8, 140.8, 128.6, 128.5, 127.7 (d, *J* = 3.2 Hz), 126.2, 125.1 (d, *J* = 7.9 Hz), 120.2, 115.7 (d, *J* = 22.9 Hz), 107.4, 31.4, 11.8 ppm. **^{19}F NMR** (470 MHz, $CDCl_3$): δ = -115.34 ppm.

MS: (EI^+) m/z = 266.1, 251.0, 247.0, 220.0, 207.0, 202.0, 196.0, 189.0, 183.0, 175.1, 159.0, 143.2, 133.1, 128.1, 123.1, 115.1, 105.0, 95.0, 91.0, 77.0, 65.0, 51.0, 43.0.

HRMS: (EI^+) m/z for $C_{18}H_{15}OF$ [M^+]: calc.: 266.1101, found: 266.1093.

IR (ATR, neat): $\tilde{\nu}$ = 3027, 2917, 1597, 1557, 1496, 1453, 1304, 1229, 1156, 1096, 1073, 1053, 1029, 1013, 963, 931, 834, 729, 697, 657, 637, 616, 591, 516 cm^{-1}.

3-Benzyl-5-isopropyl-2-phenylfuran (3t)

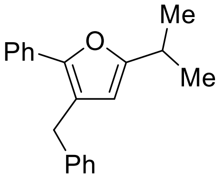

According to the general procedure 2 the title compound was prepared using 2-benzylidene-5-methyl-1-phenylhex-3-yn-1-one (274 mg, 1.0 mmol, 1.0 equiv.) at 50 °C. The product was obtained as a light yellow liquid (168 mg, 0.61 mmol, 61%).

R_f = 0.78 (*n*-pentane:diethyl ether = 40:1).

^{1}H NMR (500 MHz, $CDCl_3$): δ = 7.58–7.41 (m, 2H), 7.32–7.25 (m, 2H), 7.25–7.19 (m, 2H), 7.20–7.09 (m, 4H), 5.77 (s, 1H), 3.92 (s, 2H), 2.88 (heptd, *J* = 6.9, 1.1 Hz, 1H), 1.19 (d, *J* = 6.9 Hz, 6H) ppm. **^{13}C NMR** (126 MHz, $CDCl_3$): δ = 160.7, 147.5, 140.5, 132.0, 128.7, 128.6, 126.7, 126.2, 125.5, 120.0, 107.7, 32.2, 28.1, 21.3 ppm.

MS: (EI^+) m/z = 276.1, 261.2, 250.2, 221.2, 203.1, 178.1, 165.1, 145.1, 131.0, 115.1, 105.0,

91.1, 89.0, 77.0, 65.0, 51.0, 43.0.

HRMS: (EI^+) m/z for $C_{20}H_{20}O$ [M^+]: calc.: 276.1509, found: 276.1504.

IR (ATR, neat): $\tilde{\nu}$ = 3062, 3027, 2966, 2930, 2872, 1674, 1600, 1553, 1493, 1383, 1363, 1234, 1176, 1120, 1072, 1120, 1072, 1051, 1029, 993, 969, 911, 877, 811, 761, 727, 693, 664 cm^{-1}.

5-Cyclopropyl-2-(4-fluorophenyl)-3-(4-methoxybenzyl)furan (3u)

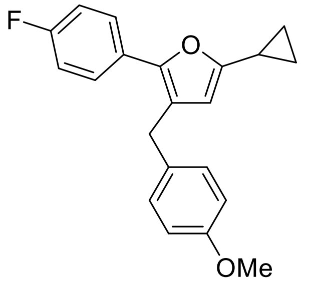

According to the general procedure 2 the title compound was prepared using 4-cyclopropyl-1-(4-fluorophenyl)-2-(4-methoxybenzylidene)but-3-yn-1-one (320 mg, 1.0 mmol, 1.0 equiv.) at rt. The product was obtained as a light yellow liquid (211 mg, 0.66 mmol, 66%).

R_f = 0.47 (*n*-pentane:diethyl ether = 40:1).

^{1}**H NMR** (500 MHz, $CDCl_3$): δ = 7.58–7.44 (m, 2H), 7.16–7.09 (m, 2H), 7.08–7.02 (m, 2H), 6.88–6.83 (m, 2H), 5.83 (s, 1H), 3.88 (s, 2H), 3.80 (s, 3H), 1.89 (tt, *J* = 8.4, 5.1 Hz, 1H), 0.92–0.86 (m, 2H), 0.86–0.78 (m, 2H) ppm. ^{13}**C NMR** (126 MHz, $CDCl_3$): δ = 161.8 (d, *J* = 246.4 Hz), 158.2, 156.3, 146.4, 132.3, 129.5, 128.1 (d, *J* = 3.2 Hz), 127.1 (d, *J* = 7.9 Hz), 120.6, 115.6 (d, *J* = 21.6 Hz), 114.1, 108.3, 55.4, 31.2, 9.1, 7.0 ppm. ^{19}**F NMR** (470 MHz, $CDCl_3$): δ = -115.31 ppm.

MS (EI^+) m/z = 322.1, 307.1, 291.1, 281.1, 269.1, 253.1, 238.0, 215.2, 183.1, 161.0, 140.0, 135.0, 123.0, 105.0, 95.0, 83.1, 77.0, 69.0, 43.0, 41.0.

HRMS: (EI^+) m/z for $C_{21}H_{19}O_2F$ [M^+]: calc.: 322.1364, found: 322.1368.

IR (ATR, neat): $\tilde{\nu}$ = 3006, 2834, 1612, 1590, 1563, 1510, 1502, 1463, 1440, 1337, 1300, 1243, 1232, 1174, 1157, 1090, 1034, 971, 904, 873, 834, 807, 763, 747, 717, 701, 669, 636, 597, 511 cm^{-1}.

5-(3-Chloropropyl)-3-(4-methylbenzyl)-2-phenylfuran (3v)

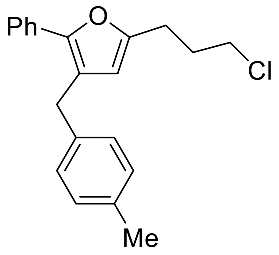

According to the general procedure 2 the title compound was prepared using 7-chloro-2-(4-methylbenzylidene)-1-phenylhept-3-yn-1-one (323 mg, 1.0 mmol, 1.0 equiv.) at rt. The product was obtained as a light yellow liquid (211 mg, 0.65 mmol, 65%).

R_f = 0.66 (*n*-pentane:diethyl ether = 40:1).

^{1}H NMR (500 MHz, $CDCl_3$): δ = 7.62–7.53 (m, 2H), 7.40–7.35 (m, 2H), 7.27–7.22 (m, 1H), 7.15–7.08 (m, 4H), 5.92 (s, 1H), 3.96 (s, 2H), 3.60 (t, *J* = 6.5 Hz, 2H), 2.82 (t, *J* = 7.3 Hz, 2H), 2.34 (s, 3H), 2.15 (p, *J* = 6.8 Hz, 2H) ppm. **^{13}C NMR** (126 MHz, $CDCl_3$): δ = 153.3, 148.2, 137.3, 135.8, 131.8, 129.4, 128.7, 128.5, 126.9, 125.6, 120.6, 110.6, 44.3, 31.7, 31.1, 25.5, 21.2 ppm.

MS: (EI^+) m/z = 324.1, 309.1, 290.1, 275.1, 261.1, 247.1, 233.0, 219.1, 204.1, 183.1, 169.0, 155.1, 141.0, 115.0, 105.0, 91.0, 77.0, 55.0, 41.0.

HRMS: (EI^+) m/z for $C_{21}H_{21}OCl$ [M^+]: calc.: 324.1275, found: 324.1281.

IR (ATR, neat): $\tilde{\nu}$ = 2957, 2920, 1600, 1556, 1513, 1490, 1444, 1294, 1217, 1082, 1072, 1021, 1009, 994, 962, 909, 757, 732, 693, 666 cm^{-1}.

5-Butyl-3-(4-methylbenzyl)-2-phenylfuran (3w)

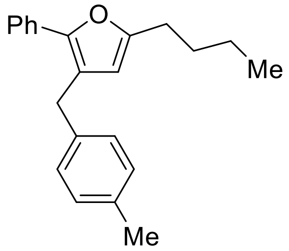

According to the general procedure 2 the title compound was prepared using 2-(4-methylbenzylidene)-1-phenyloct-3-yn-1-one (323 mg, 1.0 mmol, 1.0 equiv.) at rt. The product was obtained as a light yellow liquid (222 mg, 0.73 mmol, 73%).

R_f = 0.40 (*n*-pentane).

^{1}H NMR (500 MHz, $CDCl_3$): δ = 7.62–7.56 (m, 2H), 7.39–7.35 (m, 2H), 7.26–7.20 (m, 1H), 7.17–7.08 (m, 4H), 5.87 (s, 1H), 3.97 (s, 2H), 2.64 (t, *J* = 7.6 Hz, 2H), 2.34 (s, 3H), 1.66 (p, *J* = 7.5 Hz, 2H), 1.41 (dq, *J* = 14.7, 7.4 Hz, 2H), 0.95 (t, *J* = 7.4 Hz, 3H) ppm. **^{13}C NMR** (126 MHz, $CDCl_3$): δ = 155.6, 147.5, 137.4, 135.7, 132.0, 129.3, 128.7, 128.5, 126.7, 125.5, 120.5, 109.7, 31.8, 30.3, 28.0, 22.5, 21.2, 14.0 ppm.

MS: (EI^+) m/z = 304.2, 276.1, 261.2, 247.2, 236.2, 221.2, 203.1, 178.1, 165.1, 143.1, 119.1, 115.1, 105.0, 91.1, 77.0, 65.0, 57.1.

HRMS: (EI^+) m/z for $C_{22}H_{24}O$ [M^+]: calc.: 304.1822, found: 304.1824.

IR (ATR, neat): $\tilde{\nu}$ = 2957, 2929, 2972, 1672, 1599, 1557, 1513, 1490, 1447, 1379, 1244, 1177, 1070, 1021, 957, 910, 844, 763, 693, 643 cm^{-1}.

2-Phenyl-4,5,6,7-tetrahydrobenzofuran (3x)

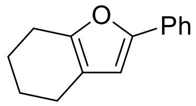

According to the general procedure 2 the title compound was prepared using 2-(phenylethynyl)cyclohex-2-en-1-one (196 mg, 1.0 mmol, 1.0 equiv.) at rt. The product was obtained as a light colourless liquid (59.1 mg, 0.30 mmol, 30%)

$\mathbf{R}_f$ = 0.65 (*n*-pentane).

^{1}H NMR (500 MHz, $CDCl_3$): δ = 7.65–7.59 (m, 2H), 7.38–7.30 (m, 2H), 7.22–7.16 (m, 1H), 6.47 (s, 1H), 2.67 (m, 2H), 2.47 (m, 2H), 1.87 (m, 2H), 1.76 (m, 2H) ppm. **^{13}C NMR** (125 MHz, $CDCl_3$): δ = 151.8, 150.9, 131.6, 128.7, 126.7, 123.4, 119.1, 106.1, 23.5, 23.3, 23.2, 22.3 ppm. The analytical data are in accordance with the literature.[9]

4 Isotope Labeling Investigation

The deuterated dihydroaromatic compounds **4** and **5** were prepared according our previous work.[10-11]

2,5-Diphenyl-3-(*p*-tolylmethyl-*d*)furan (6)

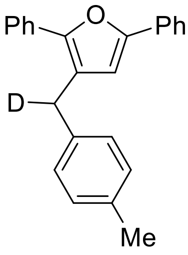

A sealed tube was charged with indium tribromide (5.3 mg, 0.015 mmol, 3.0 mol%) and a stir bar. The sealed tube was evacuated and heated to 100 °C for 30 min. Thereafter, the sealed tube was charged with N_2. Then 2.5 mL CH_2Cl_2, (1,4-dihydro-[1,1'-biphenyl]-3-yl-1-*d*)trimethylsilane (**4**, 172 mg, 0.75 mmol, 1.5 equiv.) and the 2-(4-methylbenzylidene)-1,4-diphenylbut-3-yn-1-one (**1f**, 161 mg, 0.5 mmol, 1.0 equiv.) were added in sequence. The mixture was stirred at rt or heated to 50 °C until the reaction was complete (monitored by TLC or GC-MS). Then the reaction mixture was purified by flash column chromatography (SiO_2, *n*-pentane → *n*-pentane:diethyl ether = 20:1) to afford the pure deuterated furan **6** (140 mg, 0.43 mmol, 86%, 99% D).

^{1}H NMR (500 MHz, $CDCl_3$): δ = 7.79–7.67 (m, 4H), 7.46–7.43 (m, 2H), 7.42–7.38 (m, 2H), 7.34–7.30 (m, 1H), 7.29–7.25 (m, 1H), 7.22–7.15 (m, 4H), 6.56 (s, 1H), 4.06 (d, *J* = 9.6 Hz, 1H), 2.37 (s, 3H) ppm. **^{2}H NMR** (77 MHz, $CHCl_3$:$CDCl_3$ = 20:1): δ = 4.07 (bs) ppm. **^{13}C NMR** (126 MHz, $CDCl_3$): δ = 152.4, 148.9, 136.9, 135.9, 131.5, 130.8, 129.4, 128.8, 128.6, 127.4, 127.2, 125.8, 123.8, 122.2, 110.0, 31.5 (t, *J* = 19.4 Hz), 21.2 ppm.

HRMS: (EI^+) m/z for $C_{24}H_{19}O^2H$ [M^+]: calc.: 325.1571, found: 325.1567.

3-(4-Methylbenzyl)-2,5-diphenylfuran-4-*d* (7)

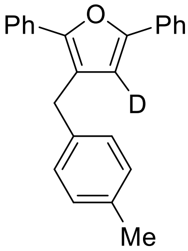

Method 1: A sealed tube was charged with indium tribromide (5.3 mg, 0.015 mmol, 3.0 mol%) and a stir bar. The sealed tube was evacuated and heated to 100 °C for 30 min. Thereafter, the sealed tube was charged with N_2. Then 2.5 mL CH_2Cl_2, (1,4-dihydro-[1,1'-biphenyl]-3-yl-4,4-d_2)trimethylsilane (**5**, 172 mg, 0.75 mmol, 1.5 equiv.) and the 2-(4-

methylbenzylidene)-1,4-diphenylbut-3-yn-1-one (**1f**, 161 mg, 0.5 mmol, 1.0 equiv.) were added in sequence. The mixture was heated to 50 °C and stirred until the reaction was complete (monitored by TLC or GC-MS). Then the reaction mixture was purified by flash column chromatography (SiO_2, n-pentane → n-pentane:diethyl ether = 20:1) to afford the pure deuterated furan **7** (141 mg, 0.433 mmol, 87%, 65% D).

Method 2: A sealed tube was charged with indium tribromide (5.3 mg, 0.015 mmol, 3.0 mol%) and a stir bar. The sealed tube was evacuated and heated to 100 °C for 30 min. Thereafter, the sealed tube was charged with N_2. Then 2.5 mL CH_2Cl_2, γ-terpinene (120 μL, 0.75 mmol, 1.5 equiv.), D_2O (18 μL, 1.0 mmol, 2.0 equiv.) and the 2-(4-methylbenzylidene)-1,4-diphenylbut-3-yn-1-one (**1f**, 161 mg, 0.5 mmol, 1.0 equiv.) were added in sequence. The mixture was heated to 50 °C and stirred until the reaction was complete (monitored by TLC or GC-MS). Then the reaction mixture was purified by flash column chromatography (SiO_2, *n*-pentane → *n*-pentane:diethyl ether = 20:1) to afford the pure deuterated furan **7** (133 mg, 0.41 mmol, 82%, 55% D).

^{1}H NMR (500 MHz, $CDCl_3$): δ = 7.80–7.61 (m, 4H), 7.47–7.35 (m, 4H), 7.33–7.23 (m, 3H), 7.20–7.12 (m, 4H), 4.06 (s, 2H), 2.35 (s, 3H) ppm. **^{2}H NMR** (77 MHz, $CHCl_3$:$CDCl_3$ = 20:1): δ = 6.58 ppm. **^{13}C NMR** (126 MHz, $CDCl_3$): δ = 152.4 (d, *J* = 7.9 Hz), 149.0, 137.0, 135.9, 131.6, 130.9, 129.4, 128.8, 128.6, 127.4, 127.3, 125.8, 123.9, 122.2 (d, *J* = 7.4 Hz), 110.1, 31.9, 31.8, 21.2 ppm.

HRMS: (EI^+) m/z for $C_{24}H_{19}O^2H$ [M^+]: calc.: 325.1571, found: 325.1567.

5 Reaction Optimization

A D-optimal screening design was generated by using *JMP 13* software package by SAS (version 13.2.1, SAS Institute Inc, Cary, NC, © 2016). The generated design considered all linear and quadratic terms of the numerical values. After running all initial experiments, the screening design was extended to consider possible cross interactions (temperature · time). All other tested cross interactions did not have high significance on the screening design. In total, the design consisted of 16 reactions, excluding six verified outliers. For the lack of fit value three experiments were duplicated.

General Procedure 3

A sealed tube was charged with indium tribromide (0.03 mmol to 0.1 mmol, 3 mol% to 10 mol%) and a stir bar. The sealed tube was evacuated and heated to 100 °C for 30 min. Thereafter, the sealed tube was charged with N_2. Then 0.25–2.5 mL CH_2Cl_2, γ-terpinene (76–120 µL, 0.475–0.75 mmol, 0.95–1.5 equiv.) and the 2-benzylidene-1,4-diphenylbut-3-yn-1-one (154 mg, 0.5 mmol, 1.0 equiv.) were added in sequence. After the desired reaction time, mesitylene (1.0 M in CH_2Cl_2, 0.5 mL, 500 µmol, 1.00 equiv.) were added via syringe. The yield of the product was determined via GC/FID. The optimal reaction conditions were tested and the product was isolated.

Table 1. Optimisation Reactions of *DoE* for the $InBr_3$-catalysed Transfer-Hydrogenation for the Synthesis of Furans.

All reactions were carried out on a 0.5 mmol scale, according to General Procedure 3. The predicted optimal reaction conditions were verified. CHD = 1,4-cyclohexadiene, GTP = γ-terpinene.

Entry	catalyst loading [mol%]	time [h]	substrate concentration [M]	reducing agent eq.	temperature [°C]	reducing agent	yield [%]
1	10	1	0.2	0.95	25	GTP	78
2	3	1	0.2	1.225	50	GTP	63
3	3	8	0.2	1.5	37.5	GTP	92
4	6.5	15	0.2	0.95	37.5	CHD	81
5	10	8	2	1.5	25	CHD	82
6	3	1	1.1	0.95	37.5	CHD	77
7	10	15	0.2	1.5	50	CHD	87
8	6.5	1	2	1.5	50	CHD	63
9	6.5	8	0.2	1.203	25	CHD	92
10	3	15	2	1.225	25	CHD	82
11	10	8	1.1	1.225	50	CHD	79
12	6.5	1	1.1	1.225	37.5	GTP	90
13	3	15	1.1	0.95	50	GTP	79
14	6.5	8	2	0.95	50	GTP	55
15	6.5	15	1.1	1.5	25	GTP	61
16	10	15	2	1.225	37.5	GTP	79
Replication	10	1	0.2	0.95	25	GTP	67
Replication	3	1	0.2	1.225	50	GTP	86
Replication	3	1	1.1	0.95	37.5	CHD	72
Replication	3	1	0.2	1.225	50	GTP	68
Replication	10	8	1.1	1.225	50	CHD	81
Replication	6.5	1	1.1	1.225	37.5	GTP	98
Optimal reaction	3	15	0.2	1.5	25	GTP	98

Response yield - Actual by Predicted Plot

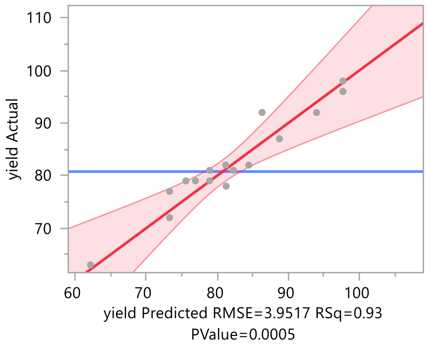

Effect Summary

Source	LogWorth	PValue
substrate concentration (0.2,2)	2.807	0.00156
time (1,15)	2.018	0.00959
catalyst loading*catalyst loading	1.729	0.01867
temperature (25,50)	1.683	0.02077
reducing agent eq. (0.95,1.5)	1.021	0.09529
time*time	0.512	0.30735
temperature*time	0.508	0.31045
catalyst loading (3,10)	0.067	0.85728 ^

Lack Of Fit

Source	DF	Sum of Squares	Mean Square	F Ratio
Lack Of Fit	5	108.43037	21.6861	3.9429
Pure Error	3	16.50000	5.5000	**Prob > F**
Total Error	8	124.93037		0.1441
				**Max RSq**
				0.9914

Residual by Predicted Plot

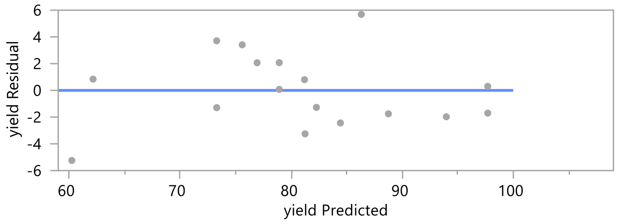

Studentized Residuals

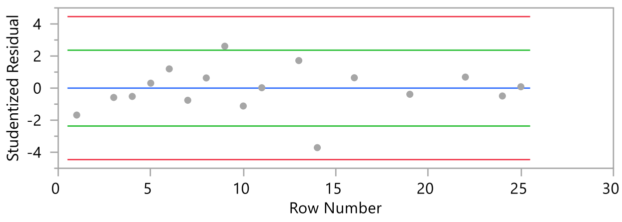

Externally studentized residuals with 95% simultaneous limits (Bonferroni) in red, individual limits in green.

Parameter Estimates

Term	Estimate	Std Error	t Ratio	Prob>|t|
Intercept	74.80685	2.461769	30.39	<.0001*
catalyst loading (3,10)	-0.244376	1.315765	-0.19	0.8573
time (1,15)	4.6561293	1.376004	3.38	0.0096*
substrate concentration (0.2,2)	-8.007134	1.707075	-4.69	0.0016*
reducing agent eq. (0.95,1.5)	2.8245839	1.493782	1.89	0.0953
temperature (25,50)	-3.727356	1.297874	-2.87	0.0208*
catalyst loading*catalyst loading	8.0926324	2.751243	2.94	0.0187*
time*time	-2.368997	2.172897	-1.09	0.3074
temperature*time	-3.288501	3.037058	-1.08	0.3105

Effect Tests

Source	Nparm	DF	Sum of Squares	F Ratio	Prob > F
catalyst loading (3,10)	1	1	0.53869	0.0345	0.8573
time (1,15)	1	1	178.80860	11.4501	0.0096*
substrate concentration (0.2,2)	1	1	343.57946	22.0013	0.0016*
reducing agent eq. (0.95,1.5)	1	1	55.83575	3.5755	0.0953
temperature (25,50)	1	1	128.79967	8.2478	0.0208*
catalyst loading*catalyst loading	1	1	135.11378	8.6521	0.0187*
time*time	1	1	18.56217	1.1886	0.3074
temperature*time	1	1	18.30913	1.1724	0.3105

Box-Cox Transformations

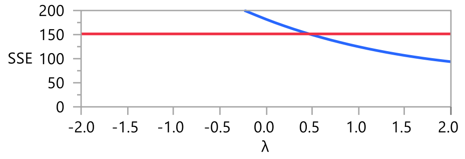

Best λ=2

Prediction Profiler

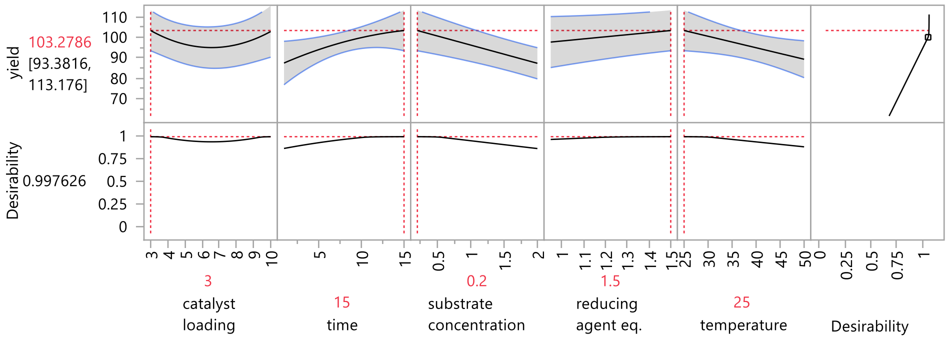

6 References

[1] T. He, P. Gao, S.-C. Zhao, Y.-D. Shi, S.-Y. Liu, Y.-M. Liang, *Adv. Synth. Catal.* **2013**, *355*, 365–369.

[2] H. Zhang, Q. Yao, L. Lin, C. Xu, X. Liu, X. Feng, *Adv. Synth. Catal.* **2017**, *359*, 3454–3459.

[3] Q. Du, J.-M. Neudörfl, H.-G. Schmalz, *Chem. Eur. J.* **2018**, *24*, 2379–2383.

[4] S. R. Pathipati, A. van der Werf, L. Eriksson, N. Selander, *Angew. Chem. Int. Ed.* **2016**, *55*, 11863–11866.

[5] P. H. Poulson, Y. Li, V. H. Lauridsen, D. K. B. Jørgensen, T. A. Palazzo, M. Meazza, K. A. Jørgensen, *Angew. Chem. Int. Ed.* **2018**, *57*, 10661–10665.

[6] T. Yao, X. Zhang, R. C. Larock, *J. Am. Chem. Soc.* **2004**, *126*, 11164–11165.

[7] S. E. Drewes, C. J. Hogan, *Synth. Commun,* **1989**, *19*, 2101–2108.

[8] R. Kotikalapudi, K. C. K. Swamy, *Tetrahedron Lett.* **2012**, *53*, 3831–3834.

[9] A. S. Dudnik, V. Gevorgyan, *Angew. Chem. Int. Ed.* **2007**, *46*, 5195–5197.

[10] L. Li, G. Hilt, *Org. Lett.* **2020**, *22*, 1628–1632.

[11] L. Li, G. Hilt, *Chem. Eur. J.* **2021**, *accepted* DOI: 10.1002/chem.202101259.

7 Spectra

2-Benzylidene-1-(4-methoxyphenyl)-4-phenylbut-3-yn-1-one (1b)

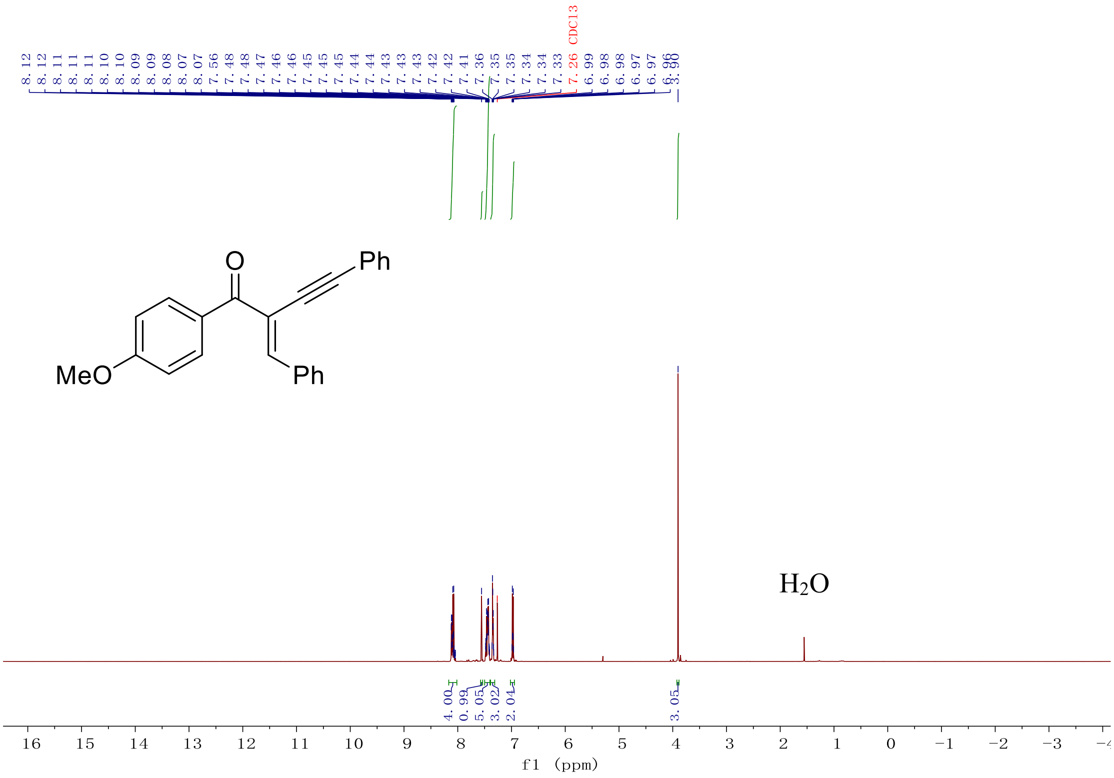

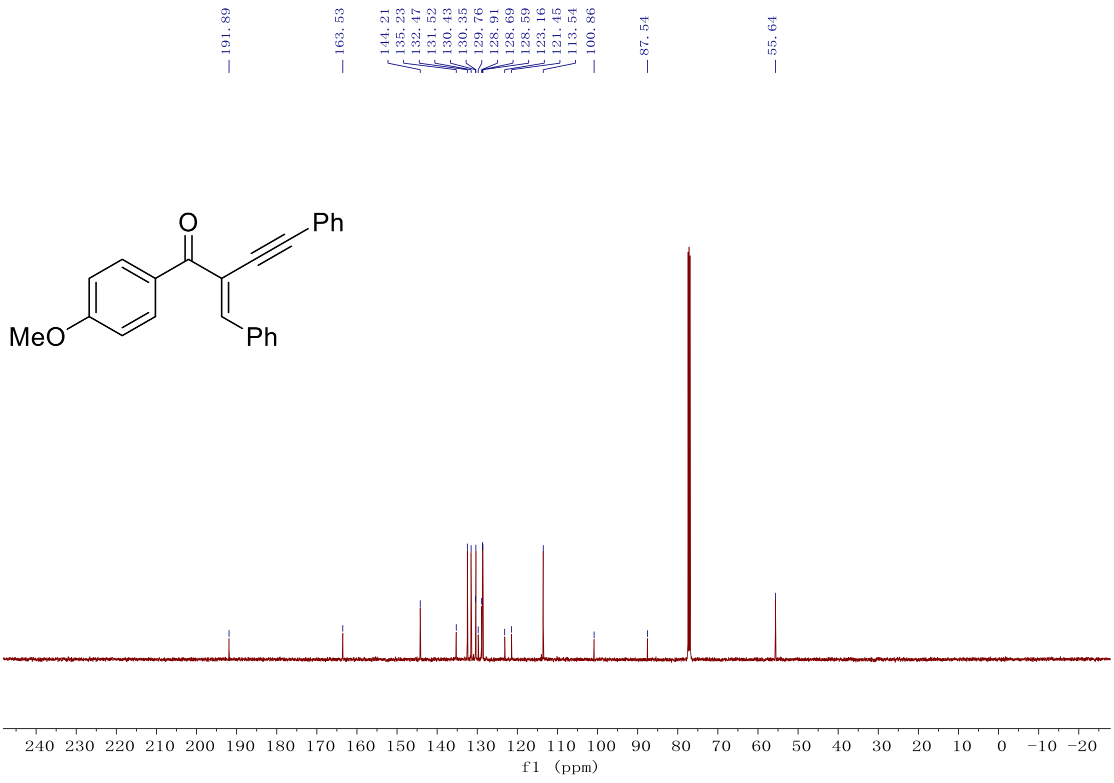

2-Benzylidene-1-(4-fluorophenyl)-4-phenylbut-3-yn-1-one (1c)

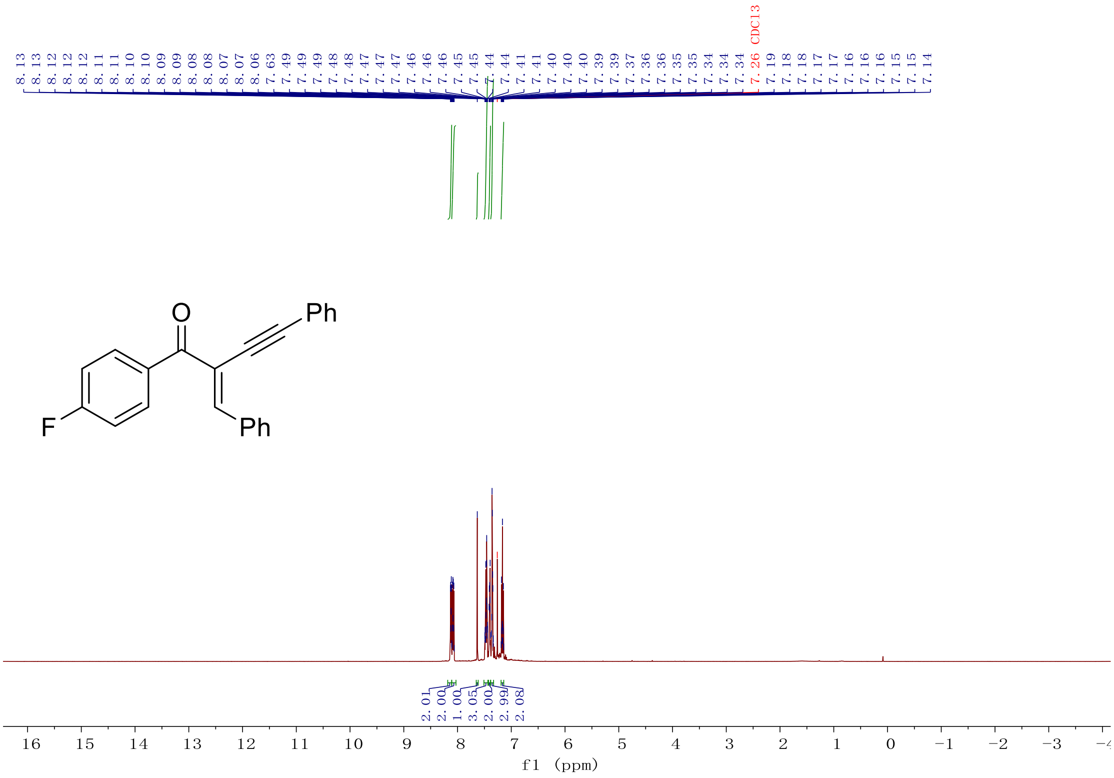

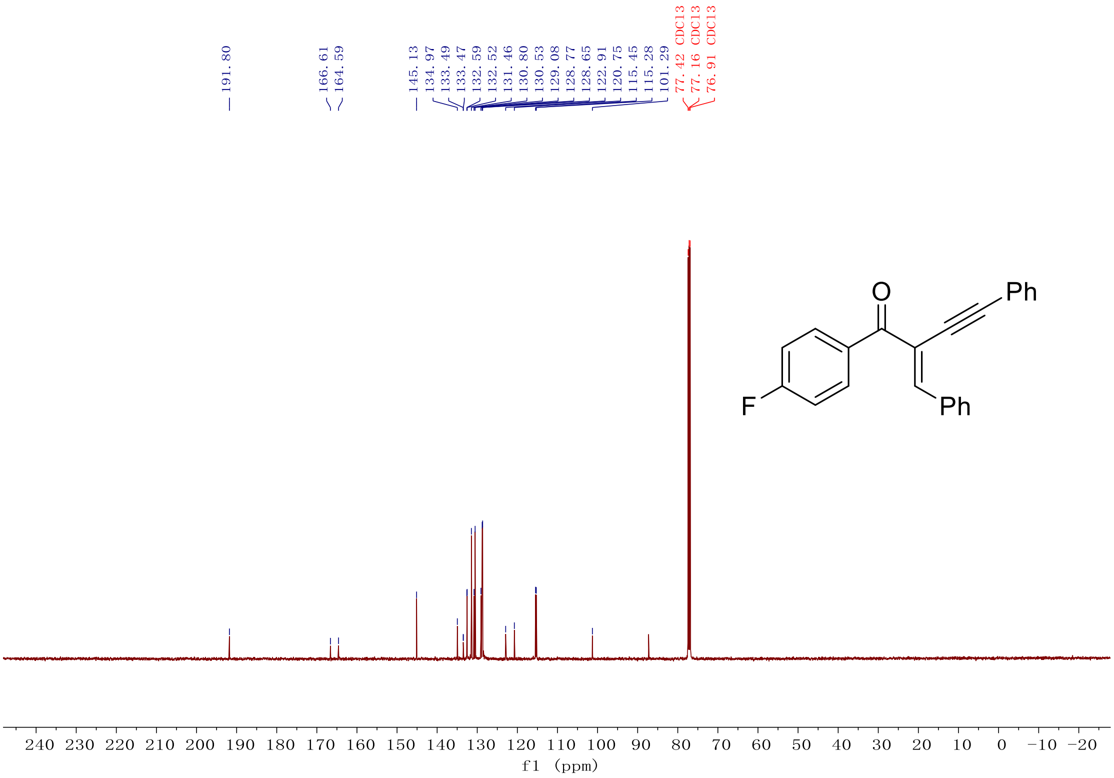

1-(4-Fluorophenyl)-2-(4-methoxybenzylidene)-4-phenylbut-3-yn-1-one (1d)

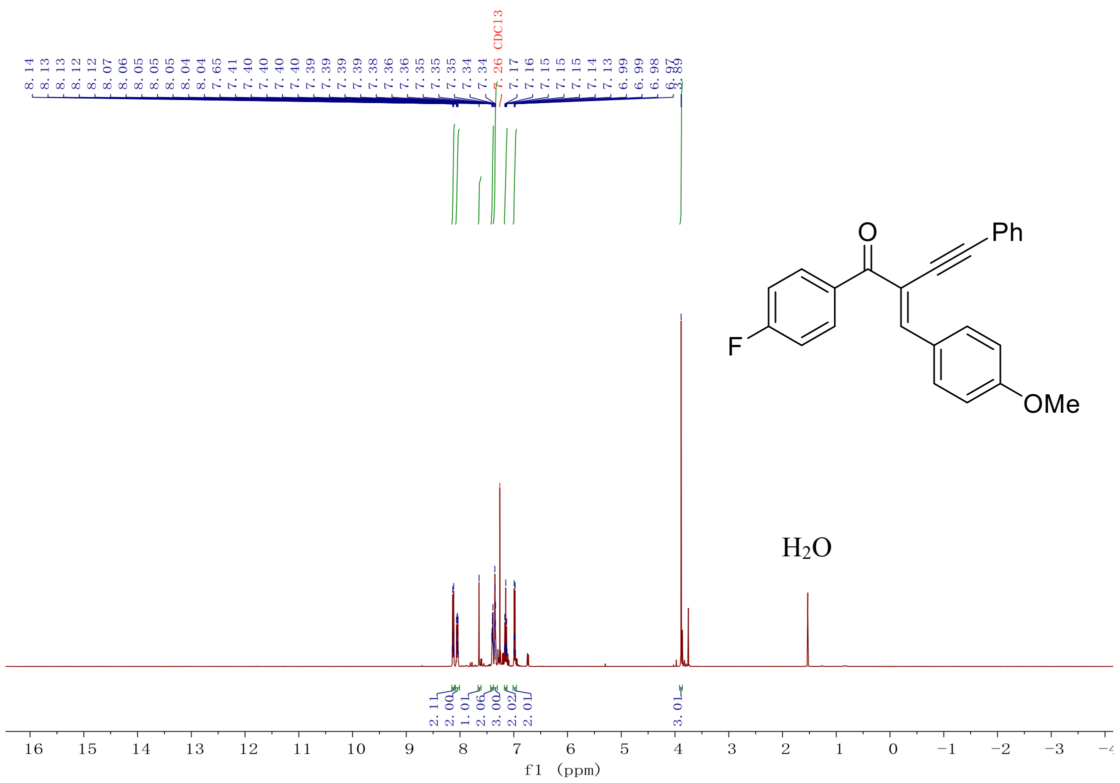

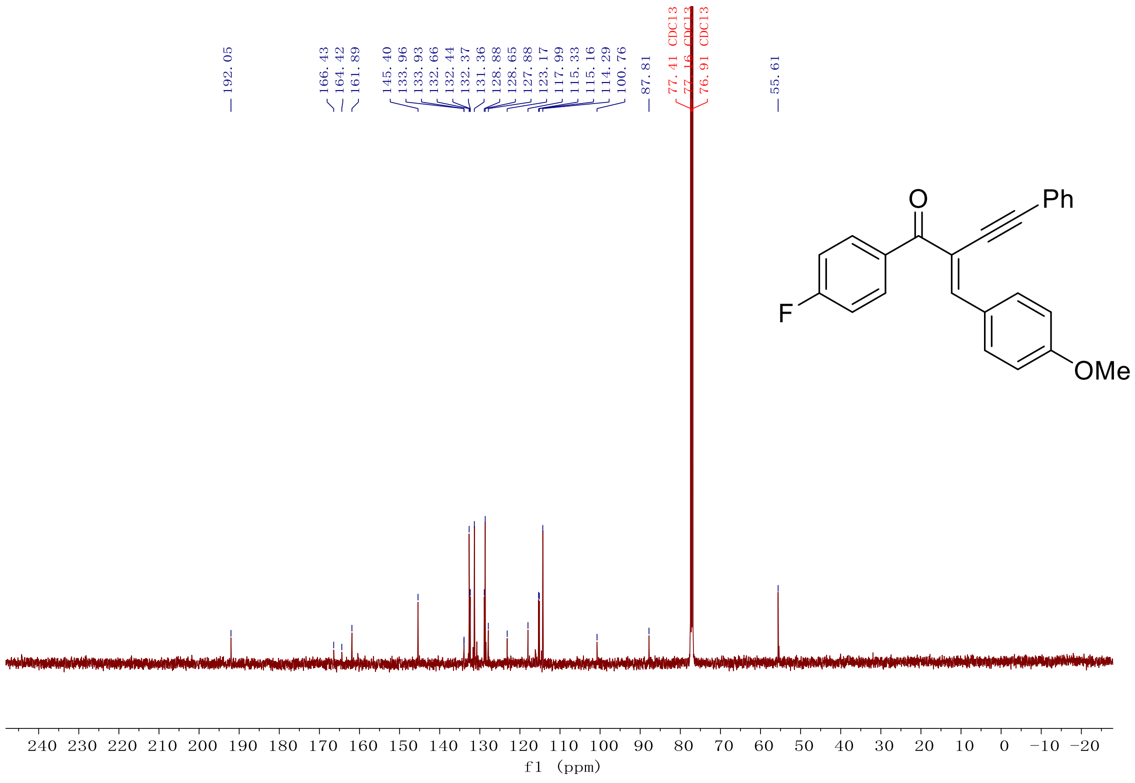

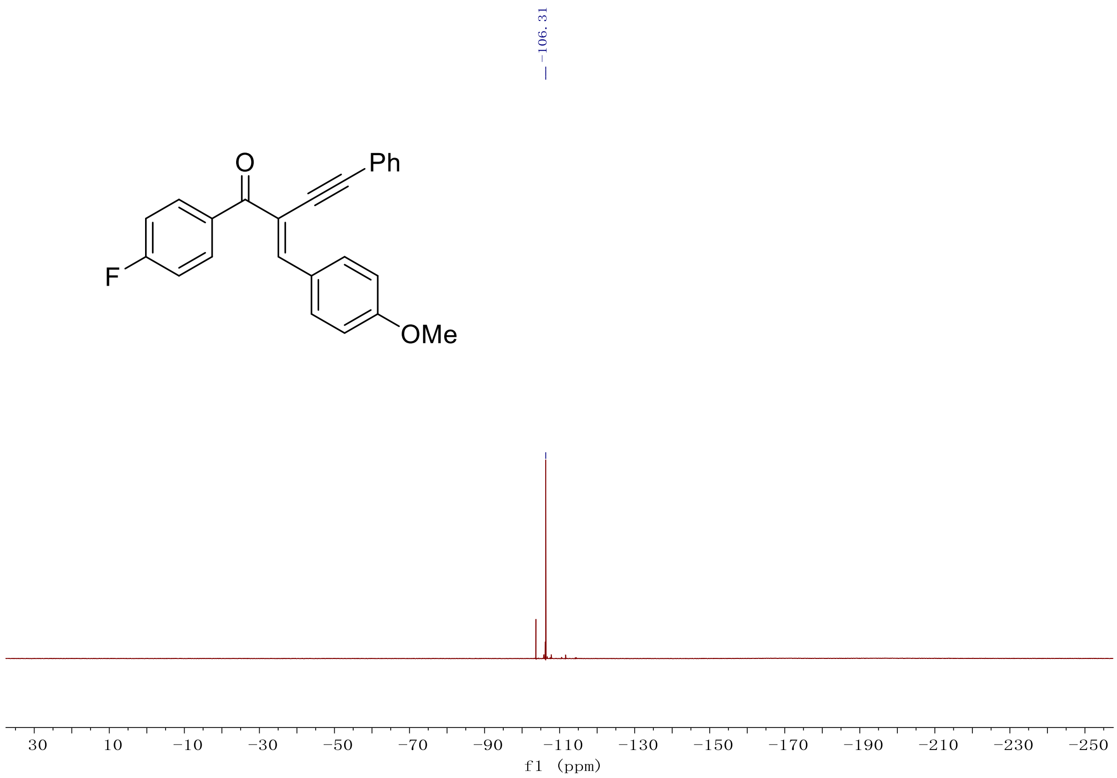

2-(4-Methoxybenzylidene)-1,4-diphenylbut-3-yn-1-one (1e)

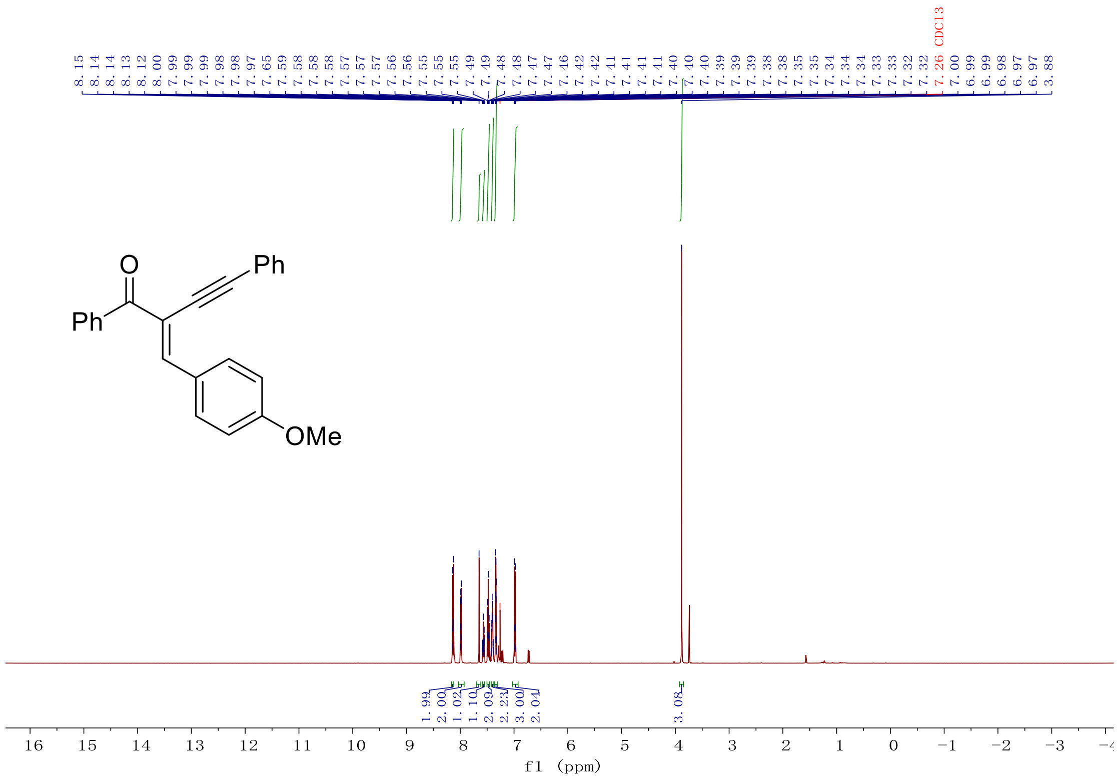

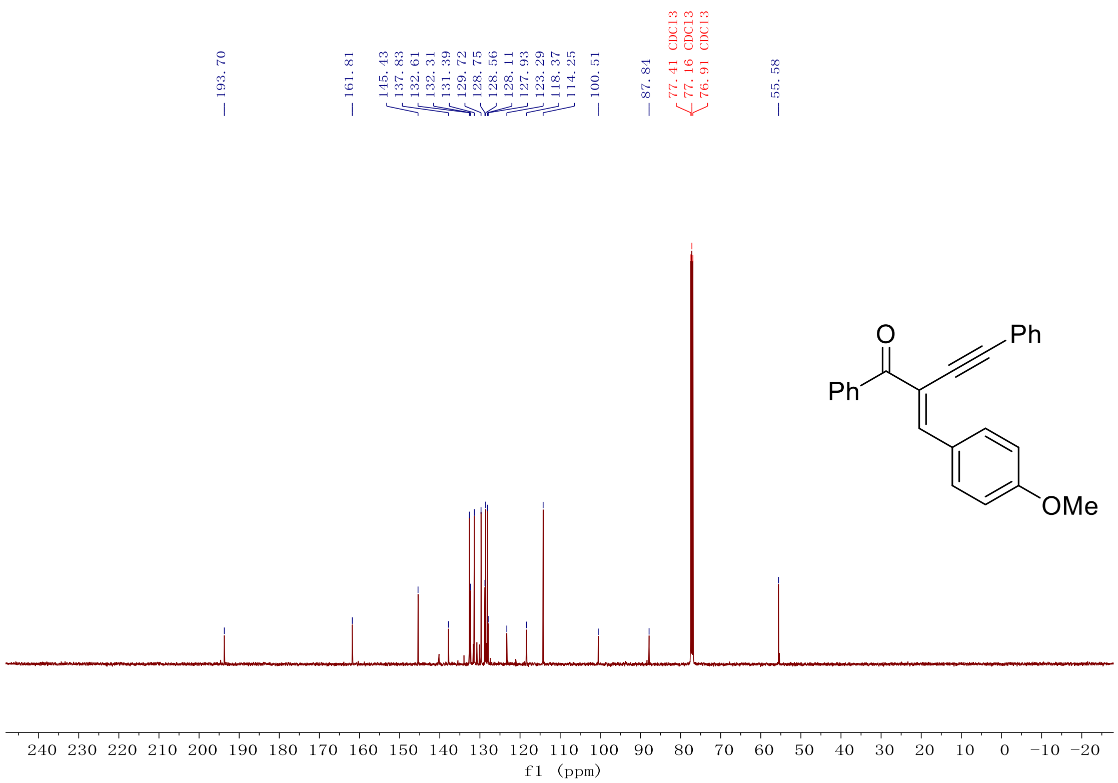

(*E, Z*)-2-(4-Methylbenzylidene)-1,4-diphenylbut-3-yn-1-one (1f)

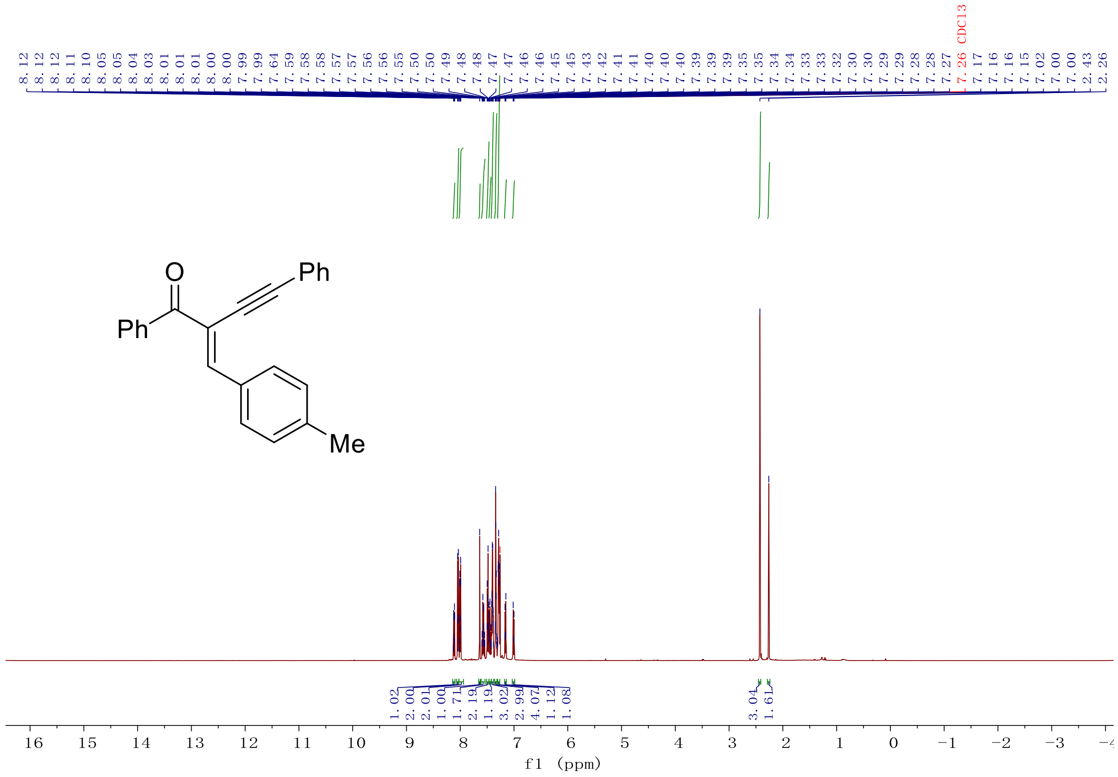

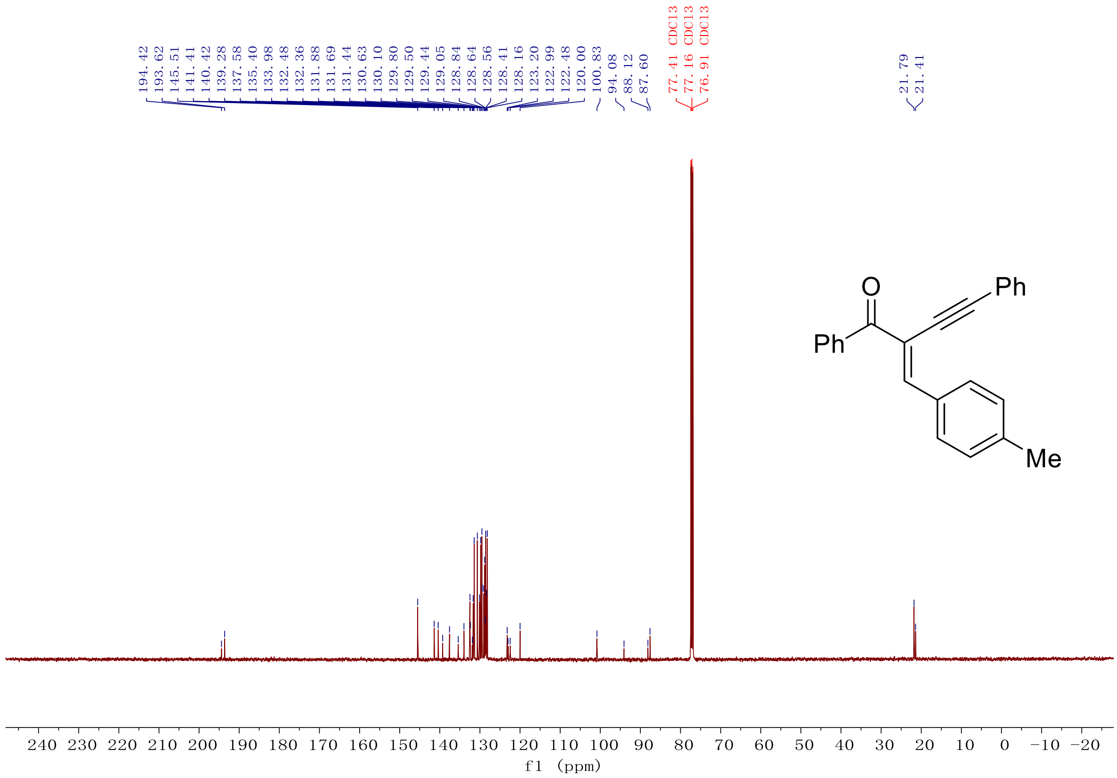

2-(4-Chlorobenzylidene)-1,4-diphenylbut-3-yn-1-one (1g)

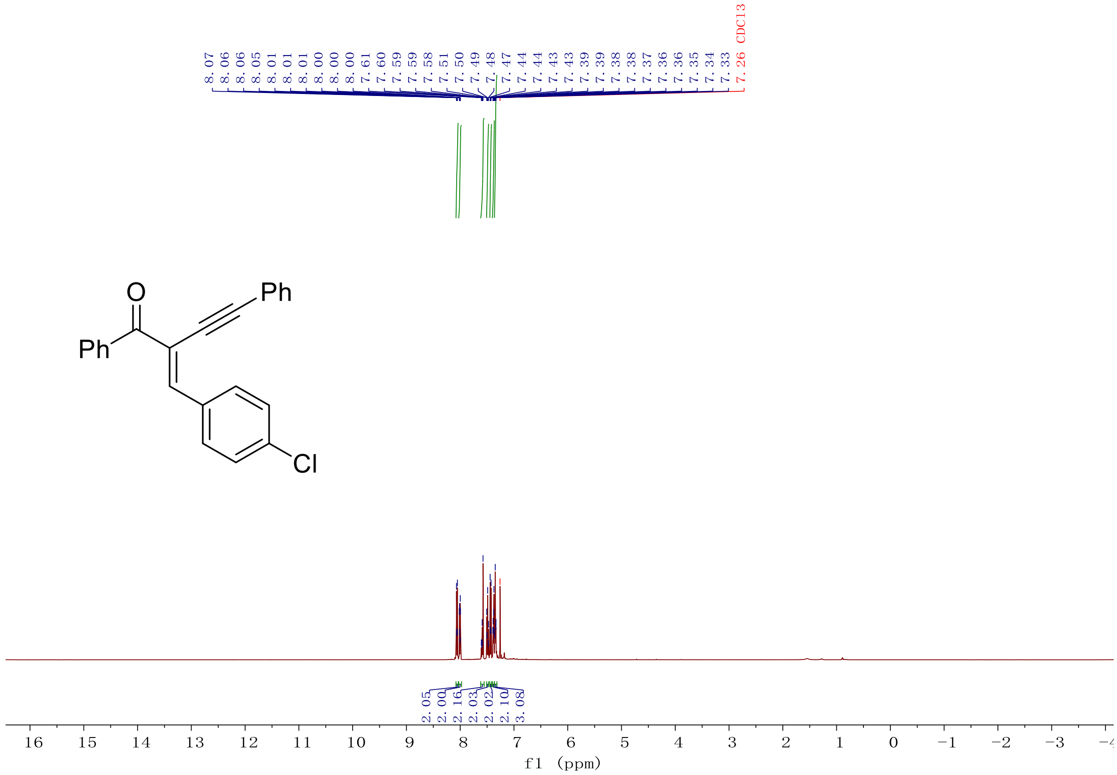

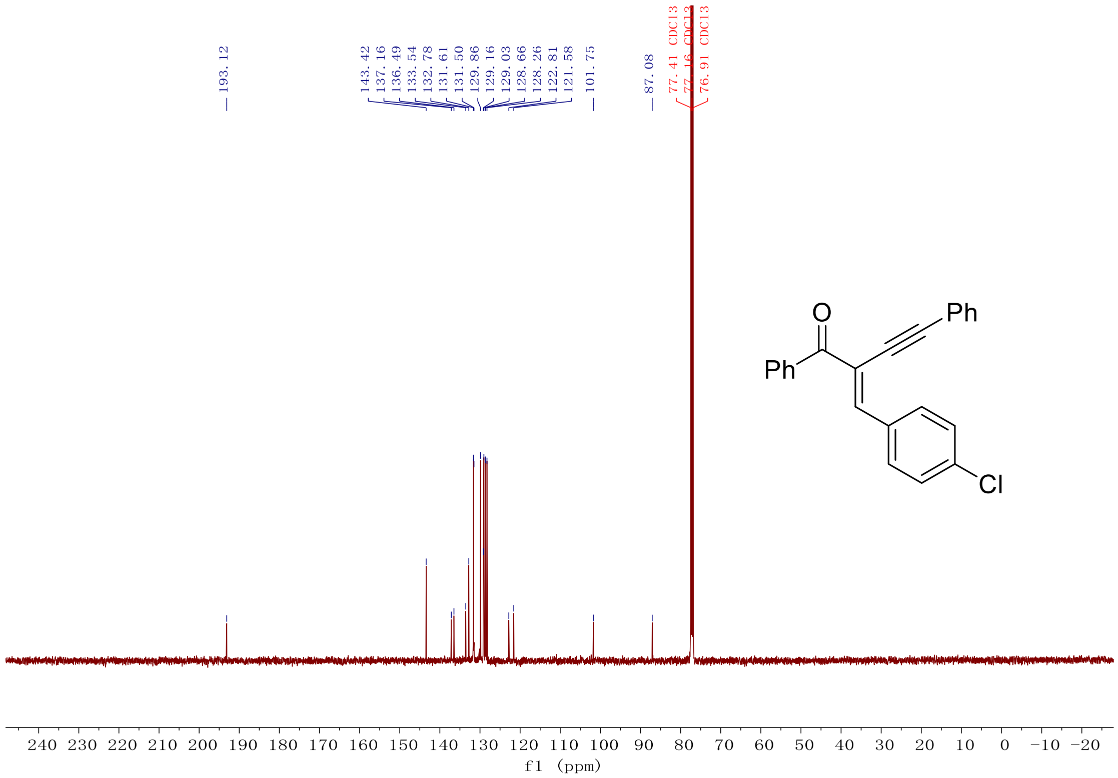

1,4-Diphenyl-2-(thiophen-2-ylmethylene)but-3-yn-1-one (1h)

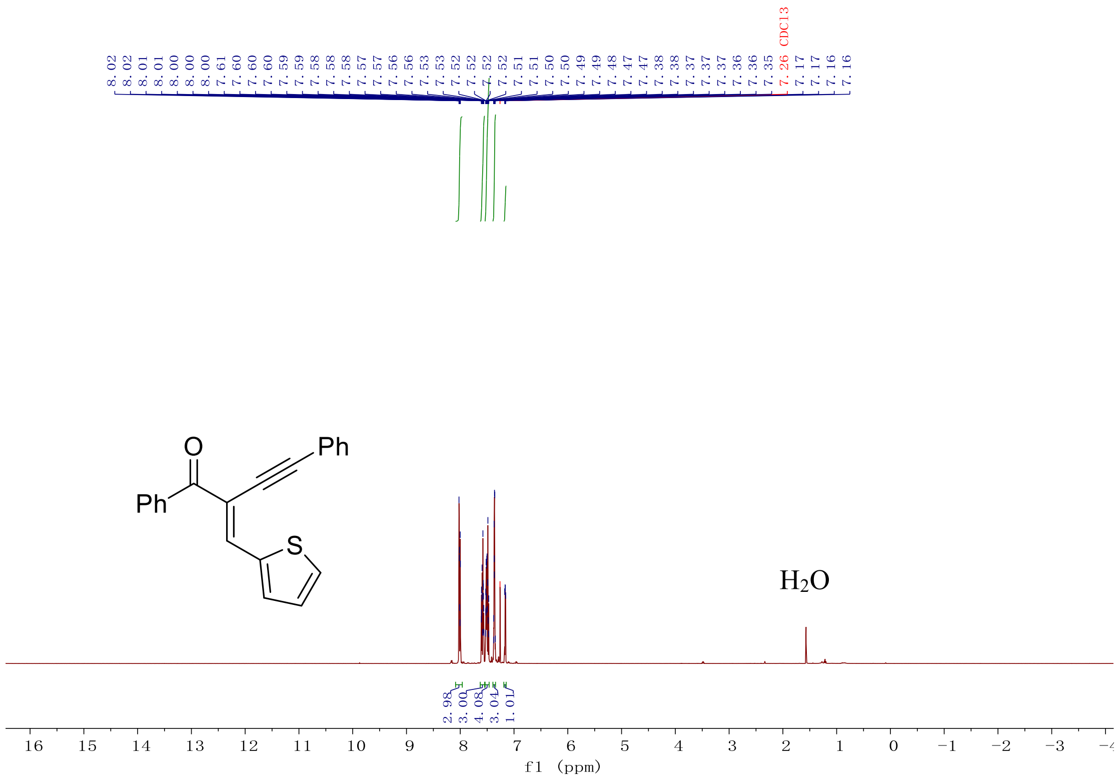

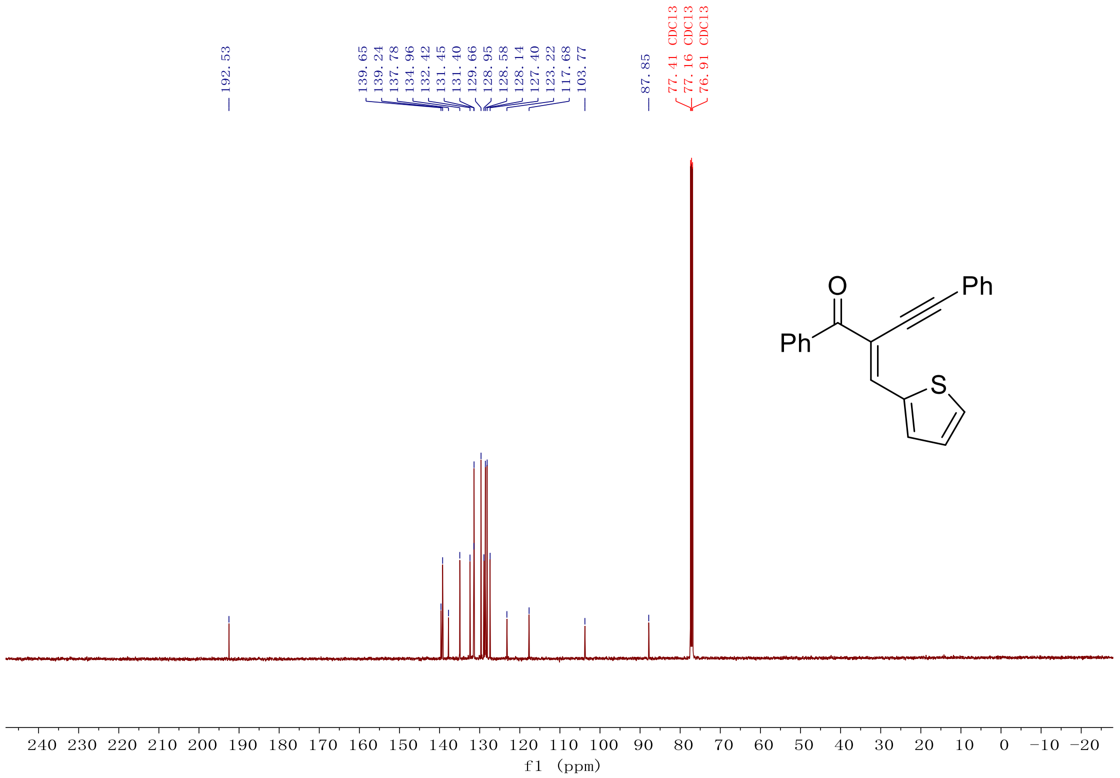

2-Benzylidene-1-phenyl-4-(4-(trifluoromethyl)phenyl)but-3-yn-1-one (1i)

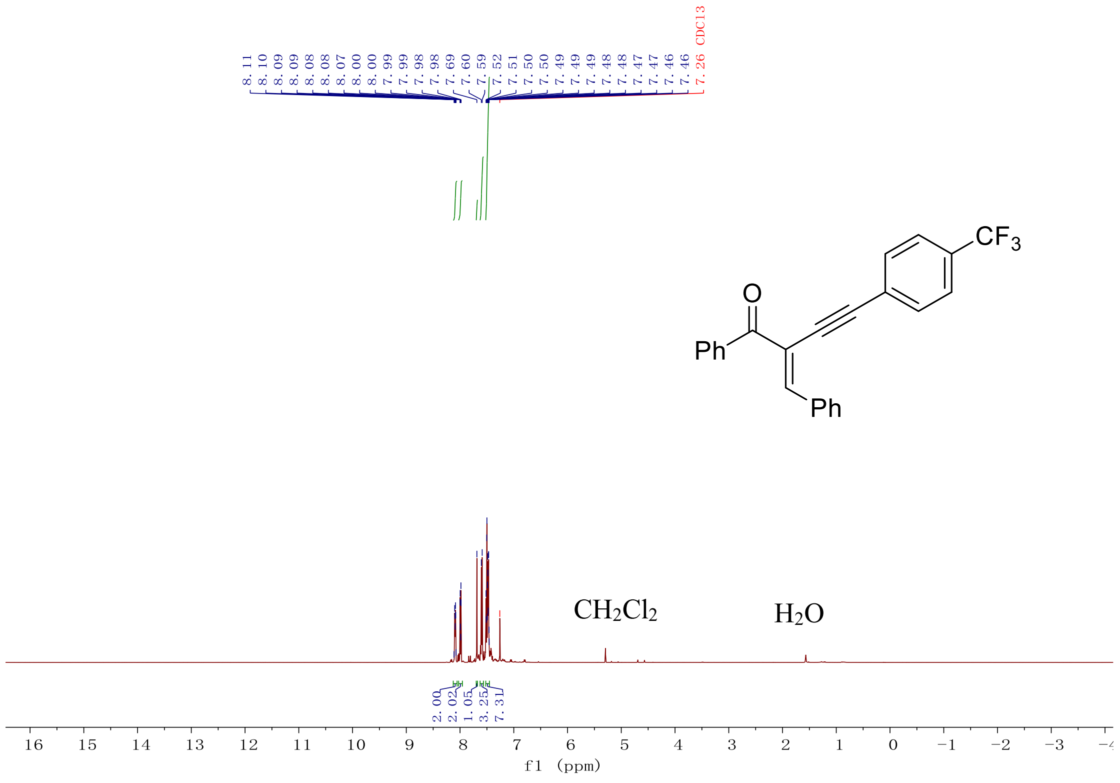

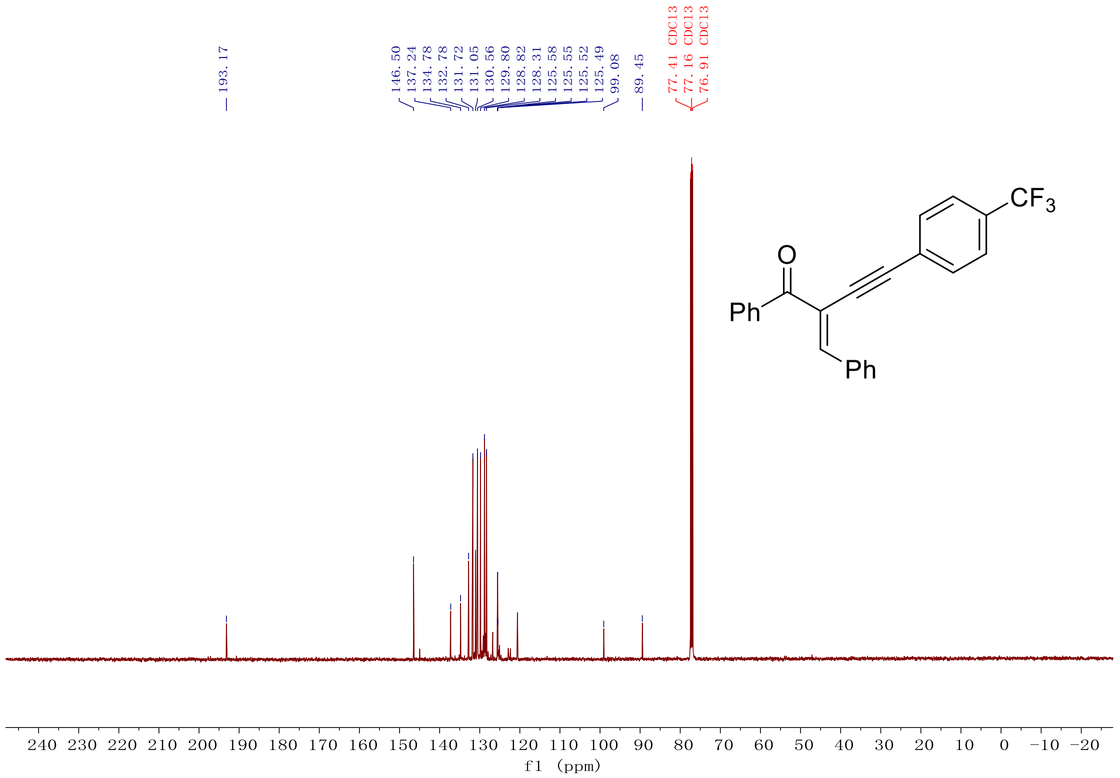

2-Benzylidene-4-(4-bromophenyl)-1-phenylbut-3-yn-1-one (1j)

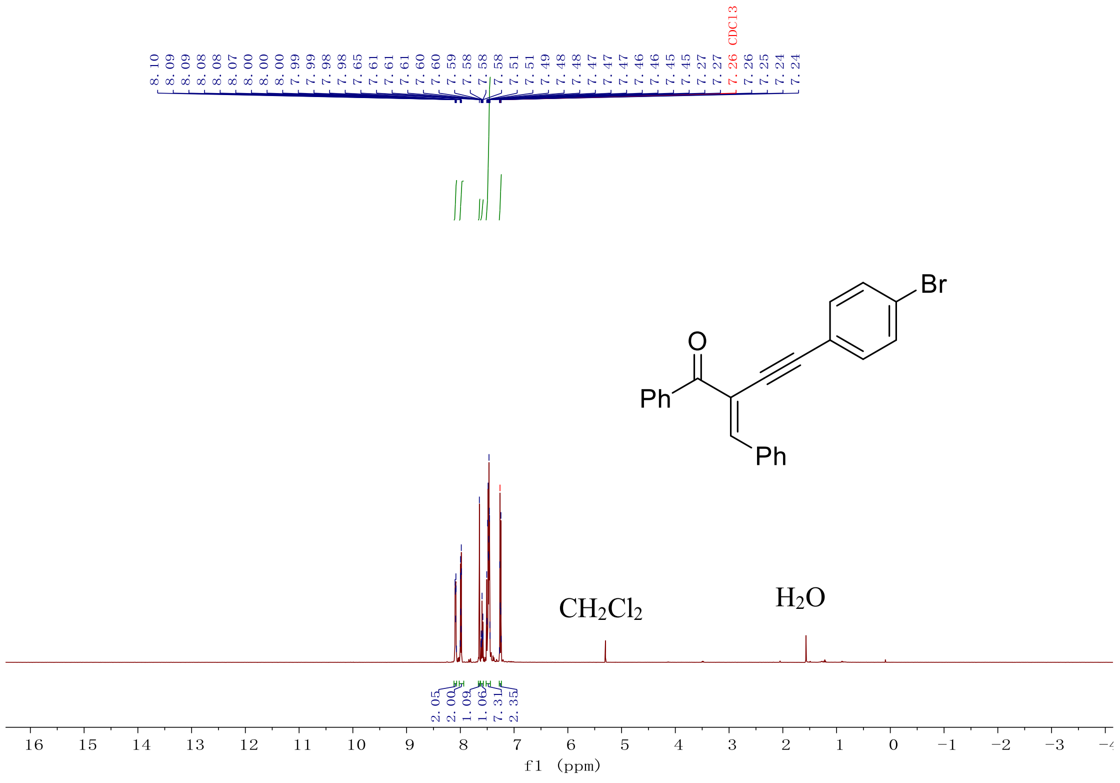

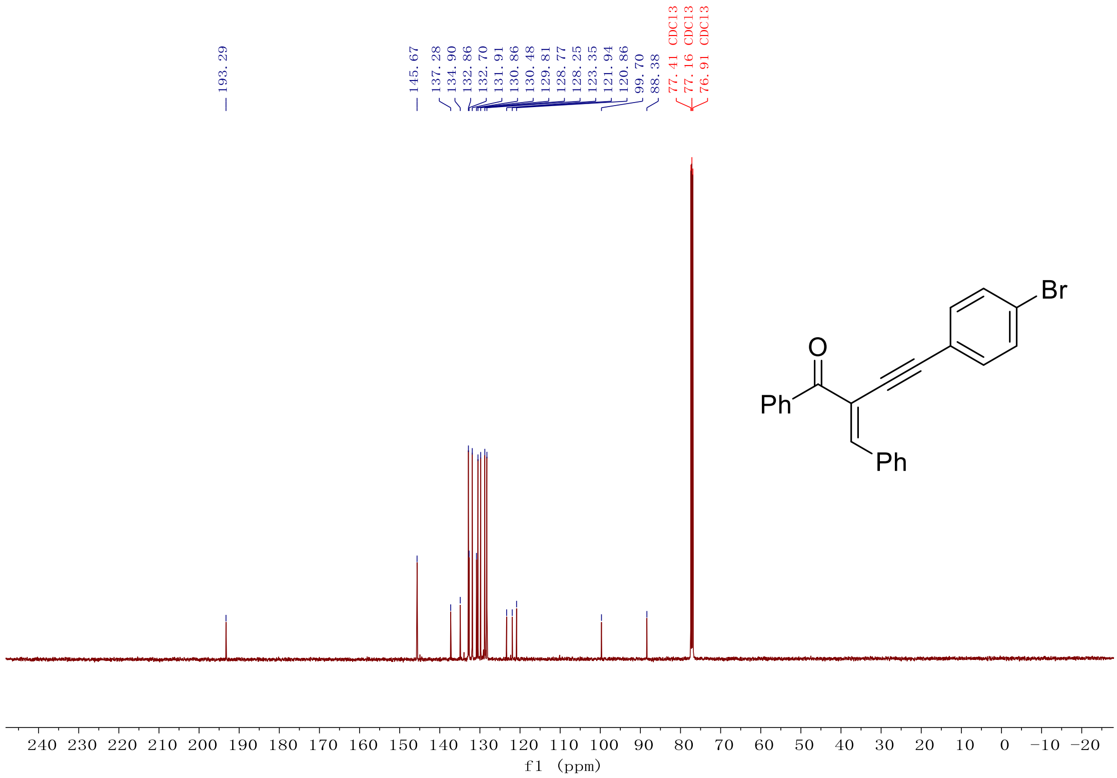

2-Benzylidene-1-phenyl-4-(*m*-tolyl)but-3-yn-1-one (1l)

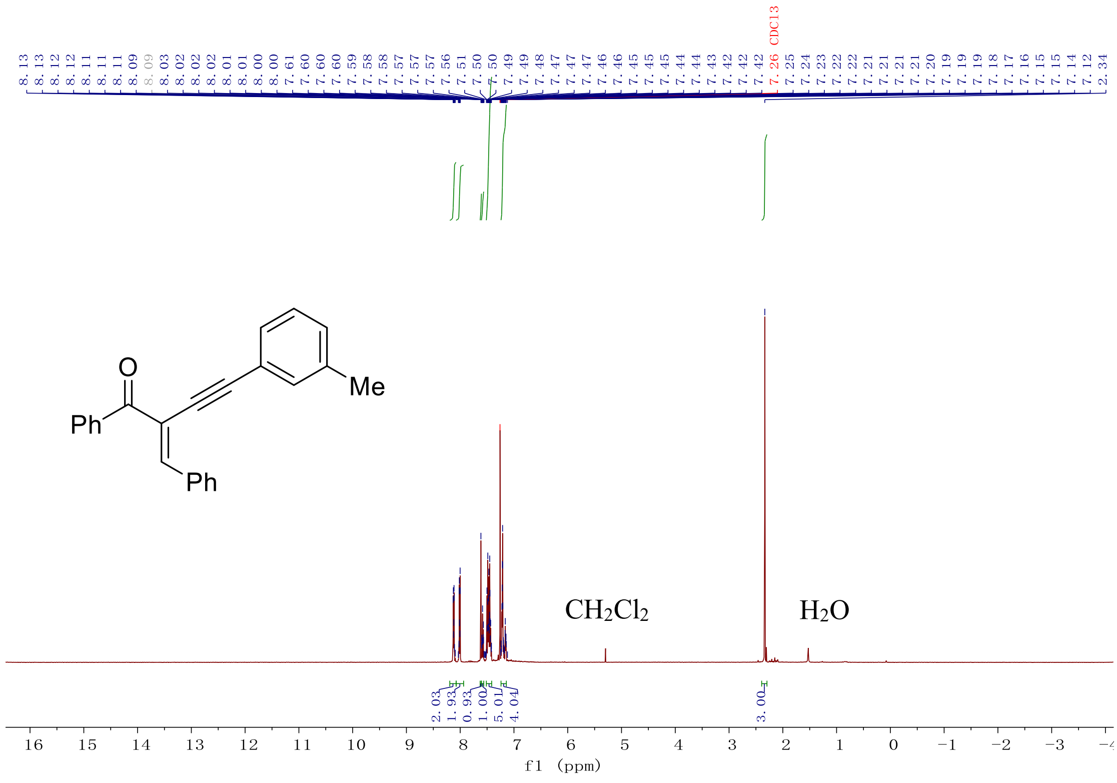

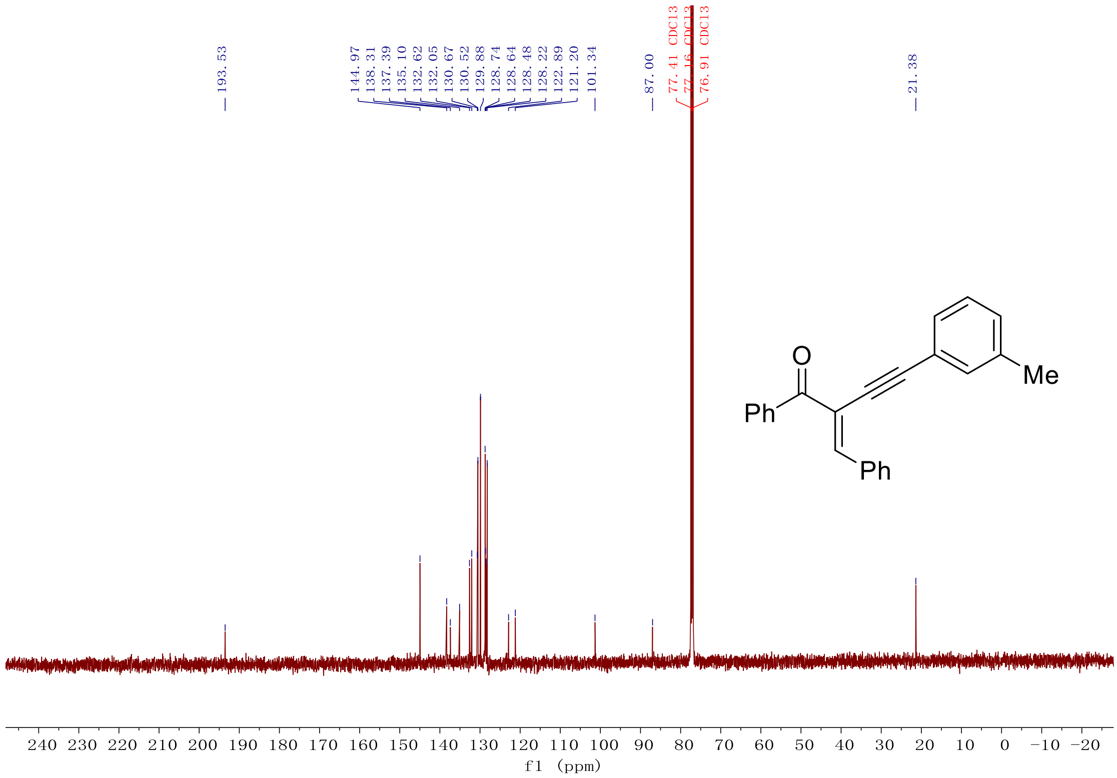

2-Benzylidene-4-(4-methoxyphenyl)-1-phenylbut-3-yn-1-one (1m)

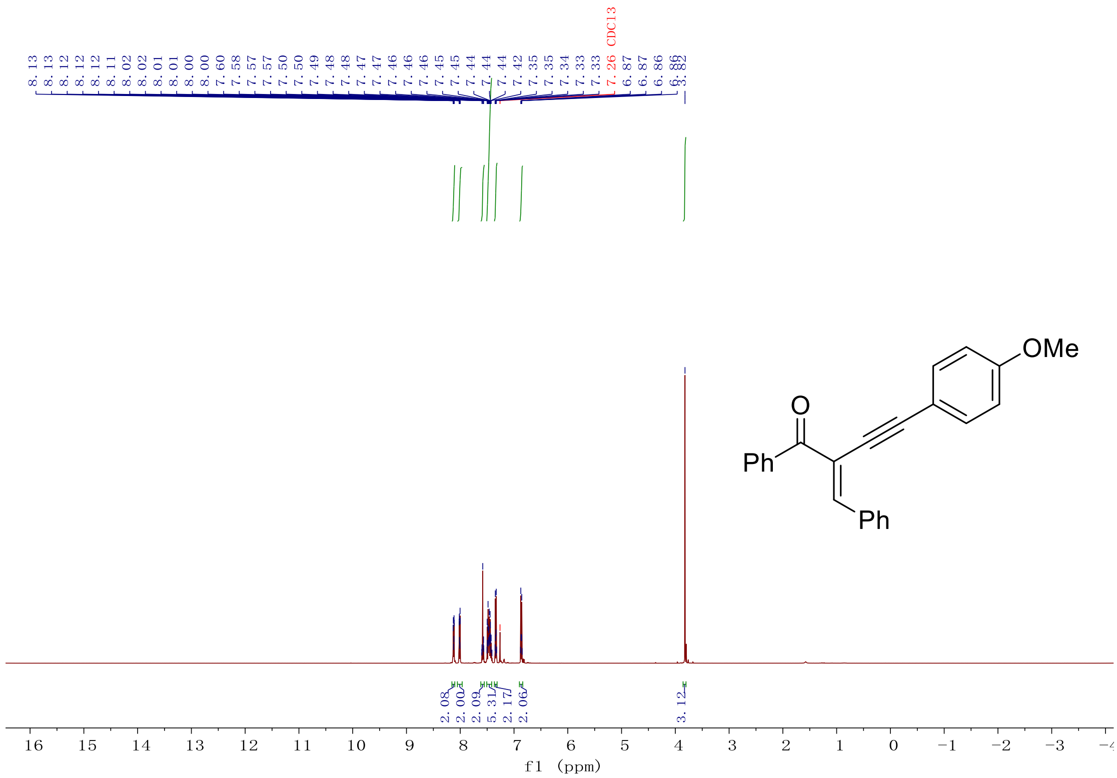

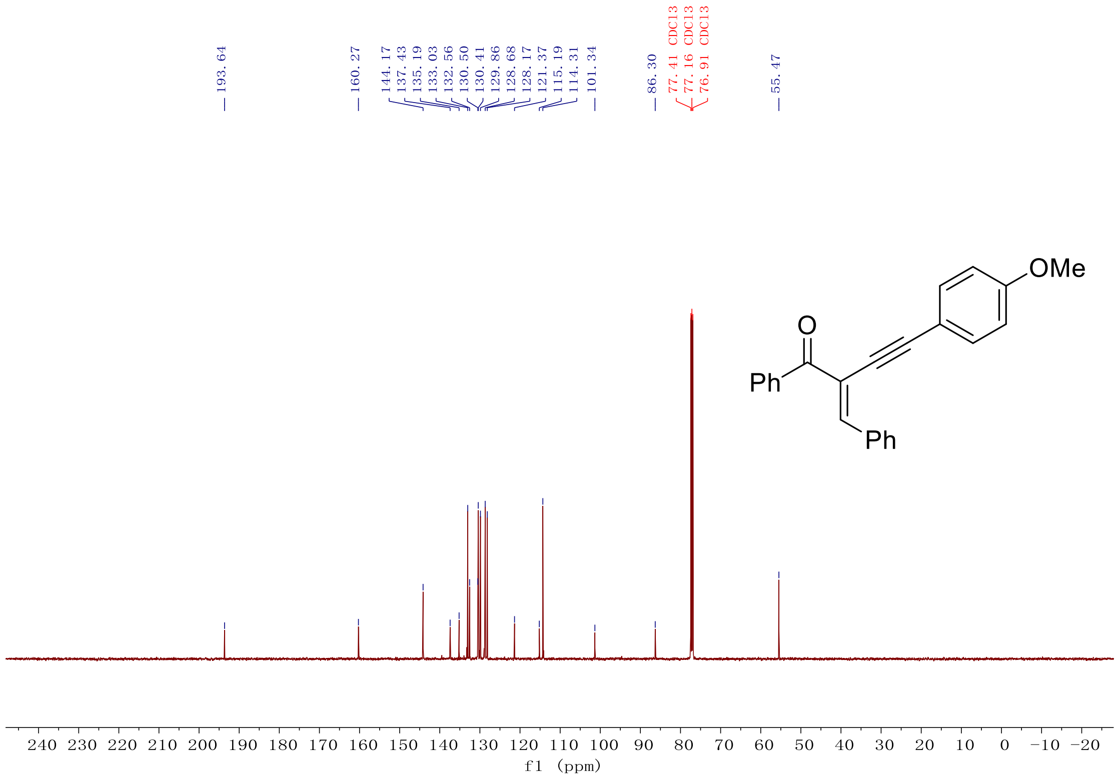

3-(2-Chlorobenzylidene)-5-phenylpent-4-yn-2-one (1p)

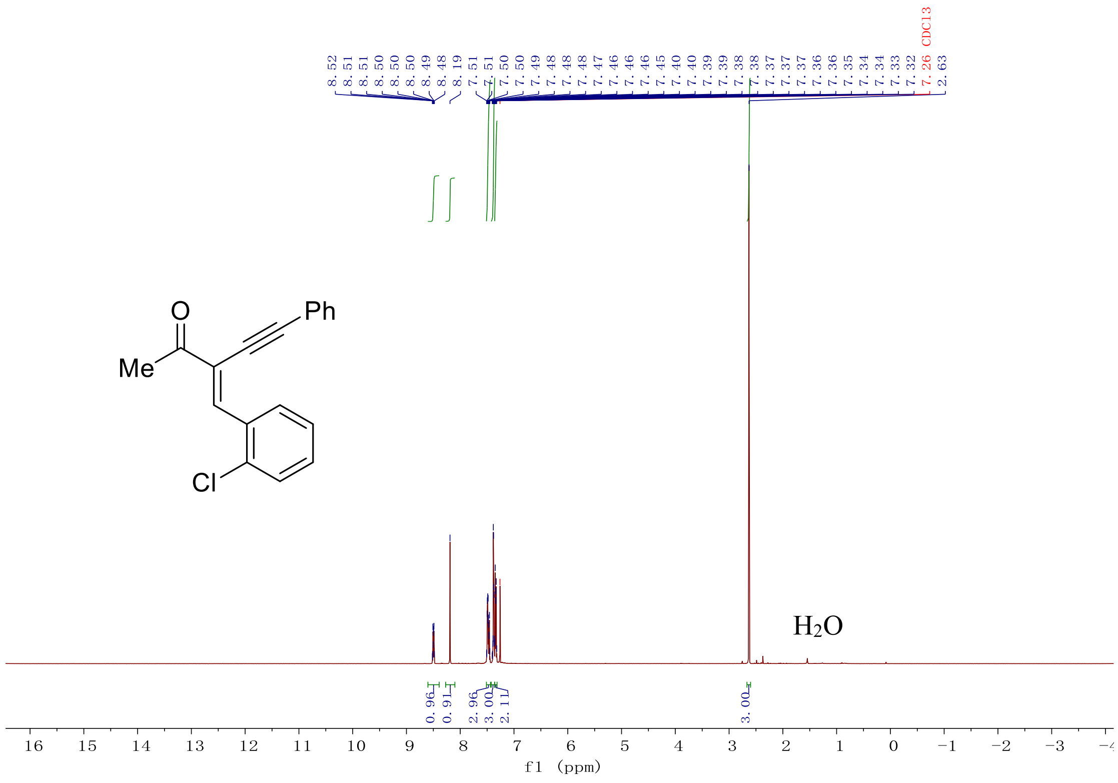

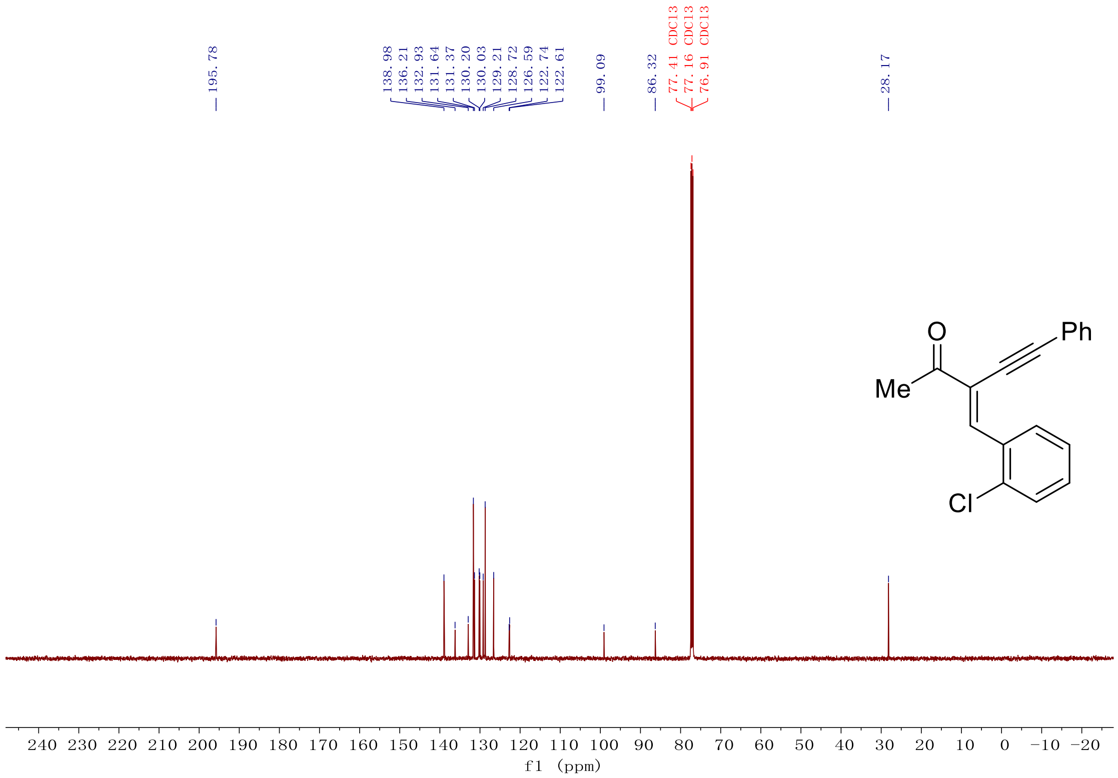

3-(Benzo[*d*][1,3]dioxol-5-ylmethylene)-5-phenylpent-4-yn-2-one (1r)

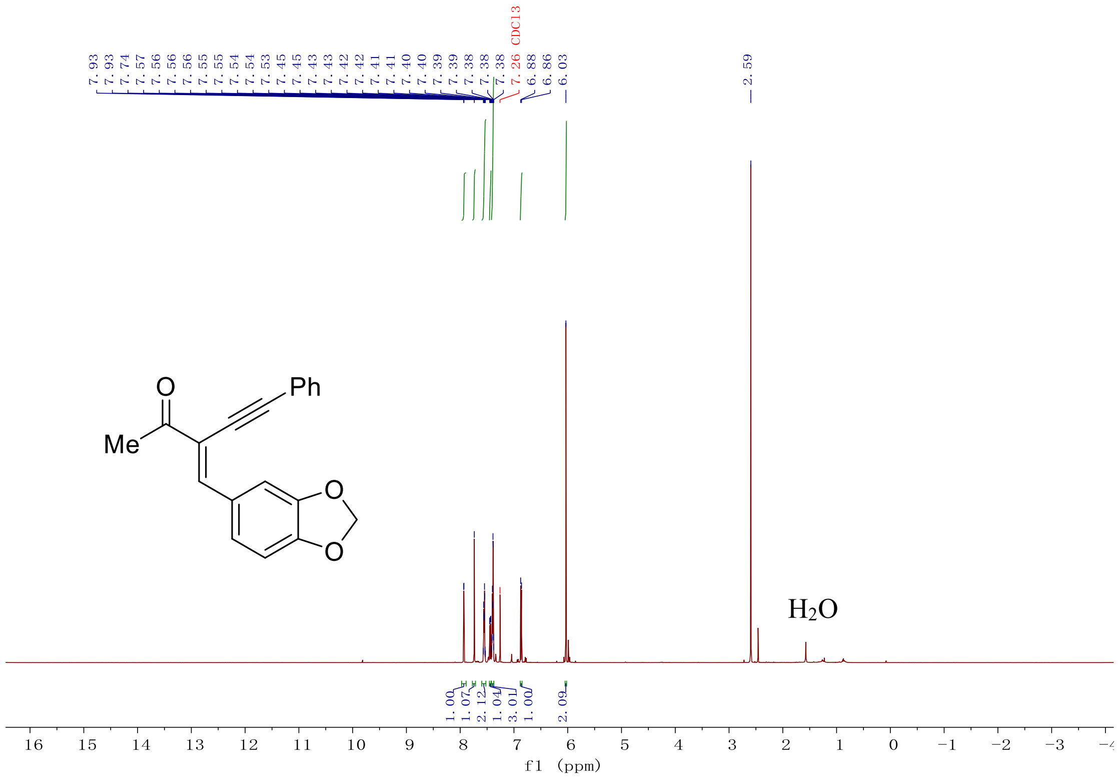

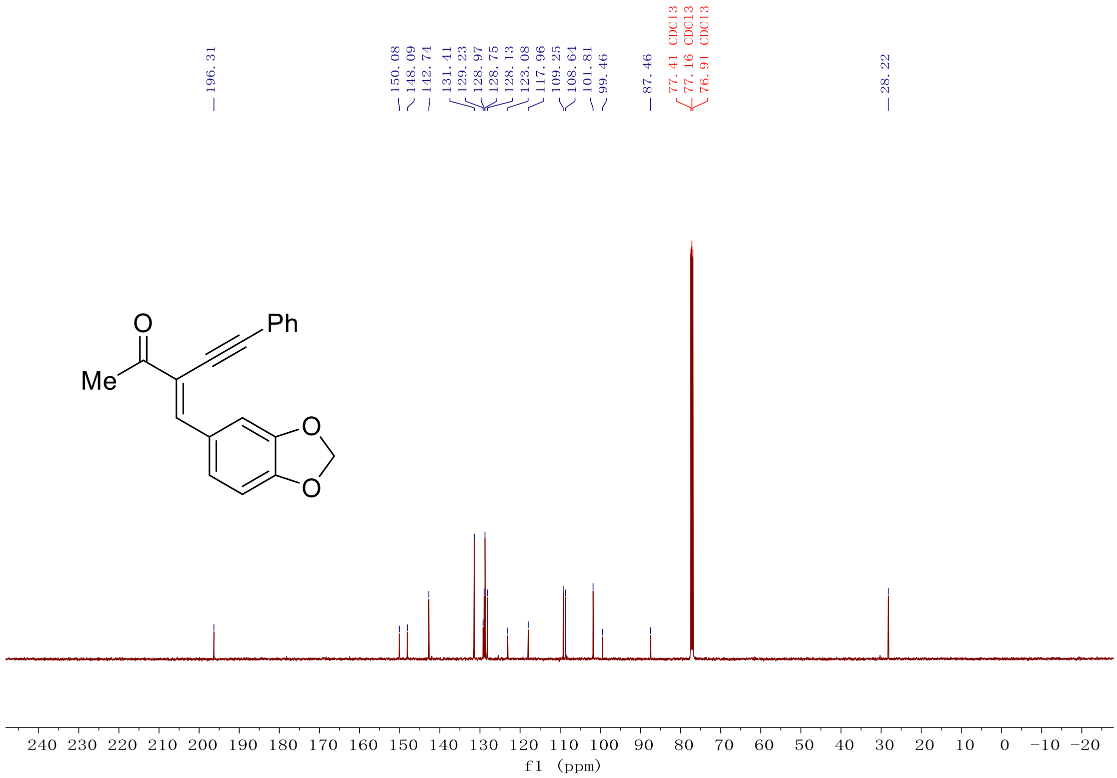

2-Benzylidene-5-methyl-1-phenylhex-3-yn-1-one (1t)

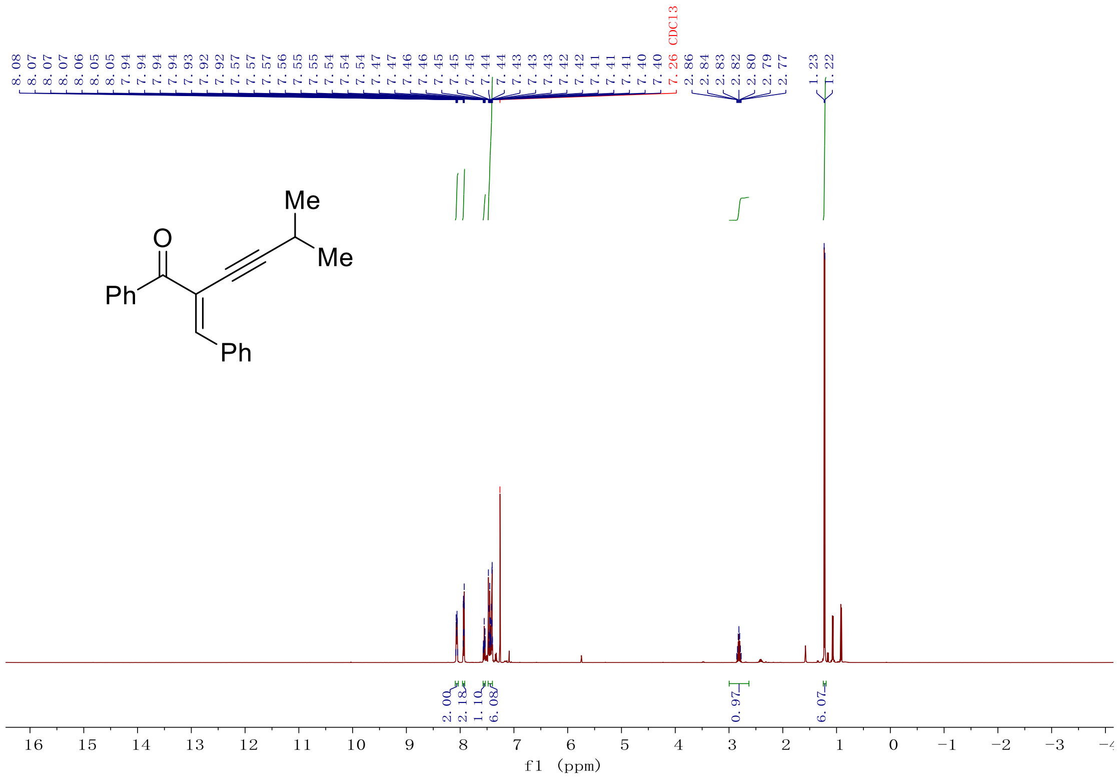

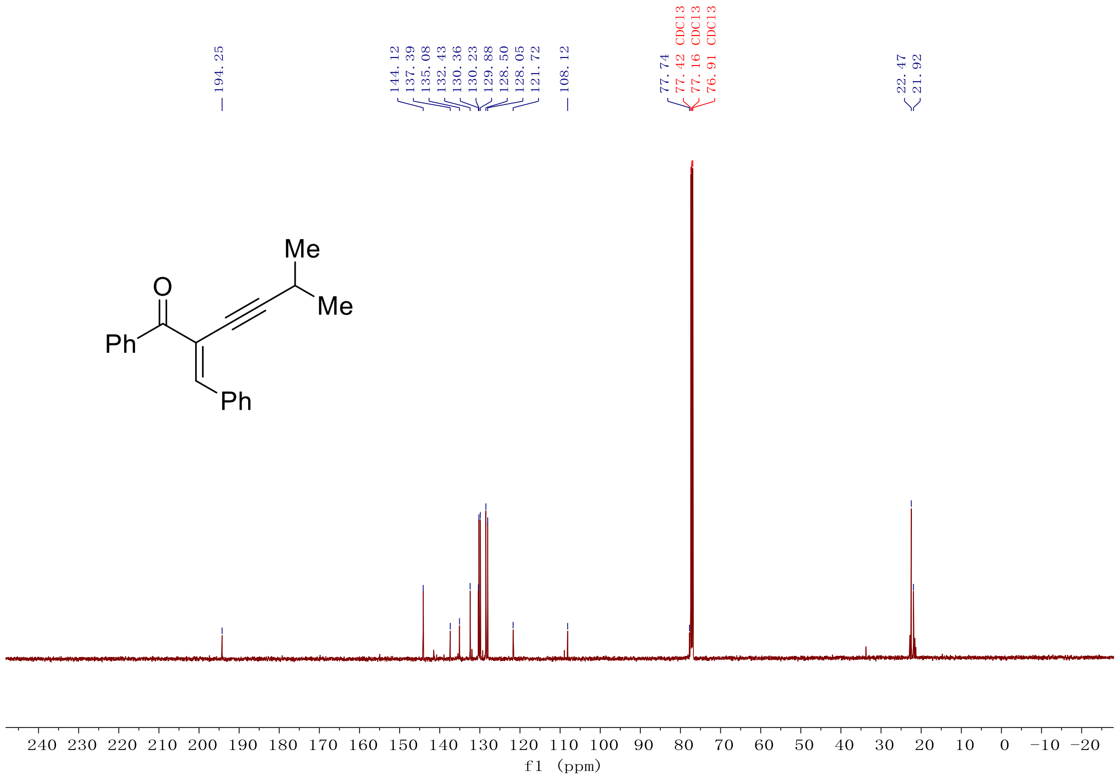

4-Cyclopropyl-1-(4-fluorophenyl)-2-(4-methoxybenzylidene)but-3-yn-1-one (1u)

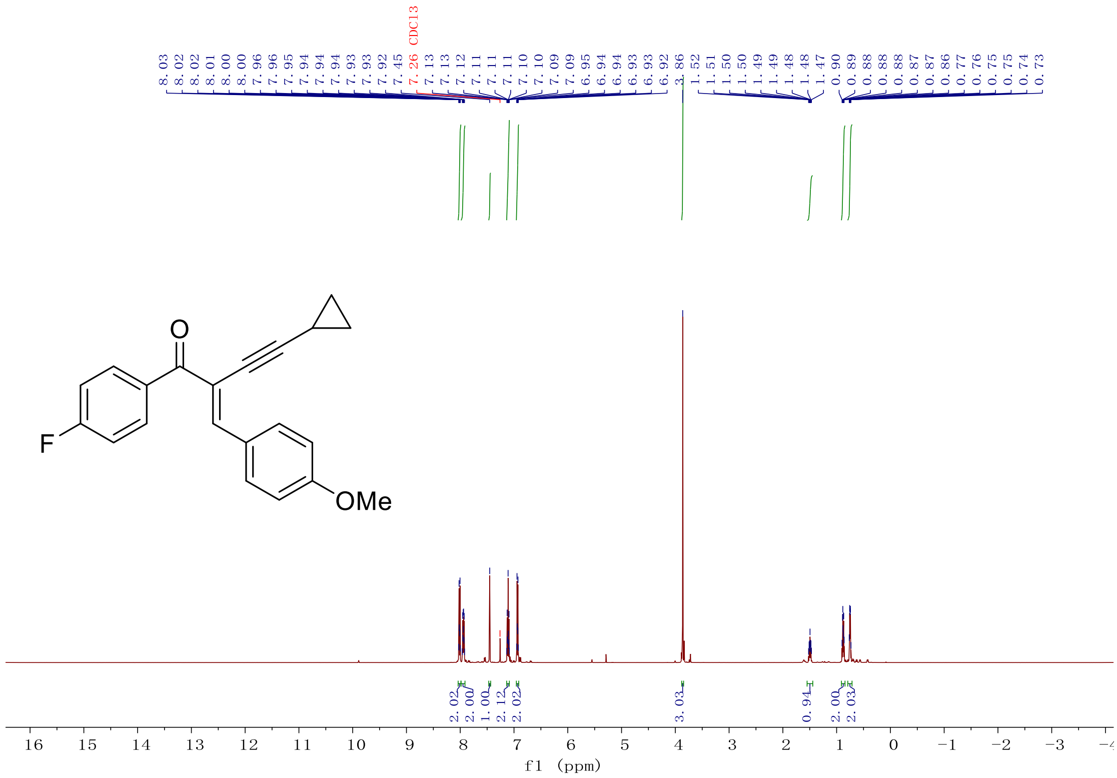

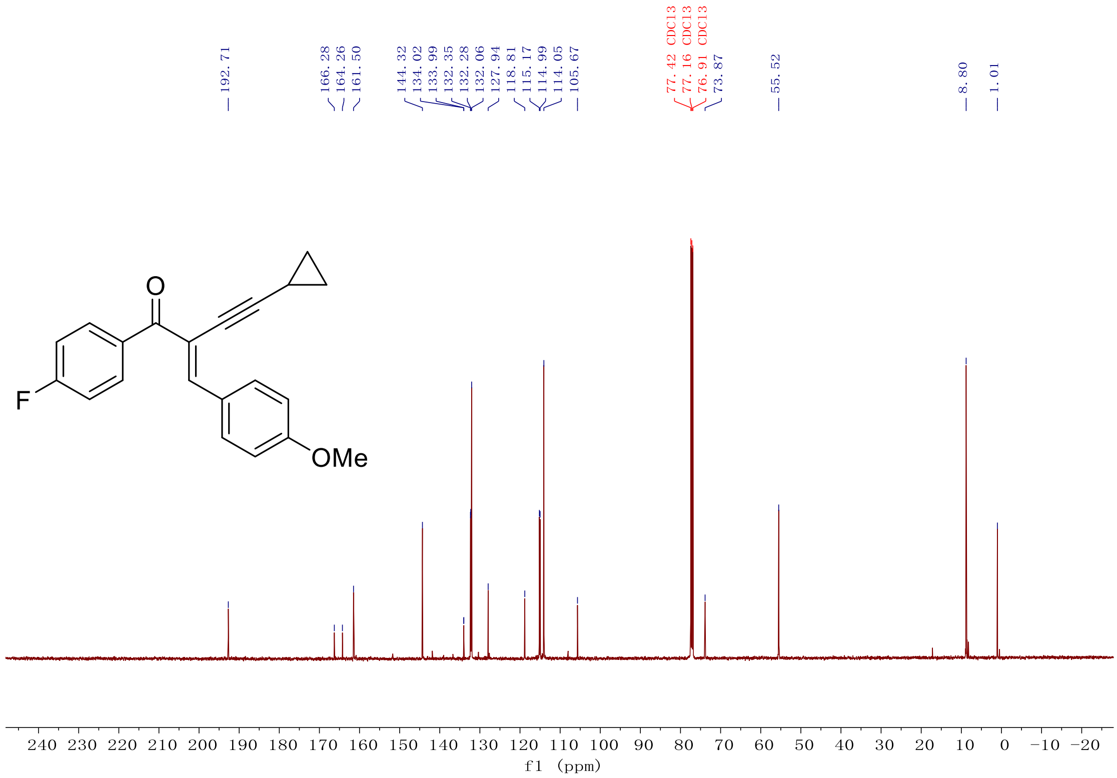

7-Chloro-2-(4-methylbenzylidene)-1-phenylhept-3-yn-1-one (1v)

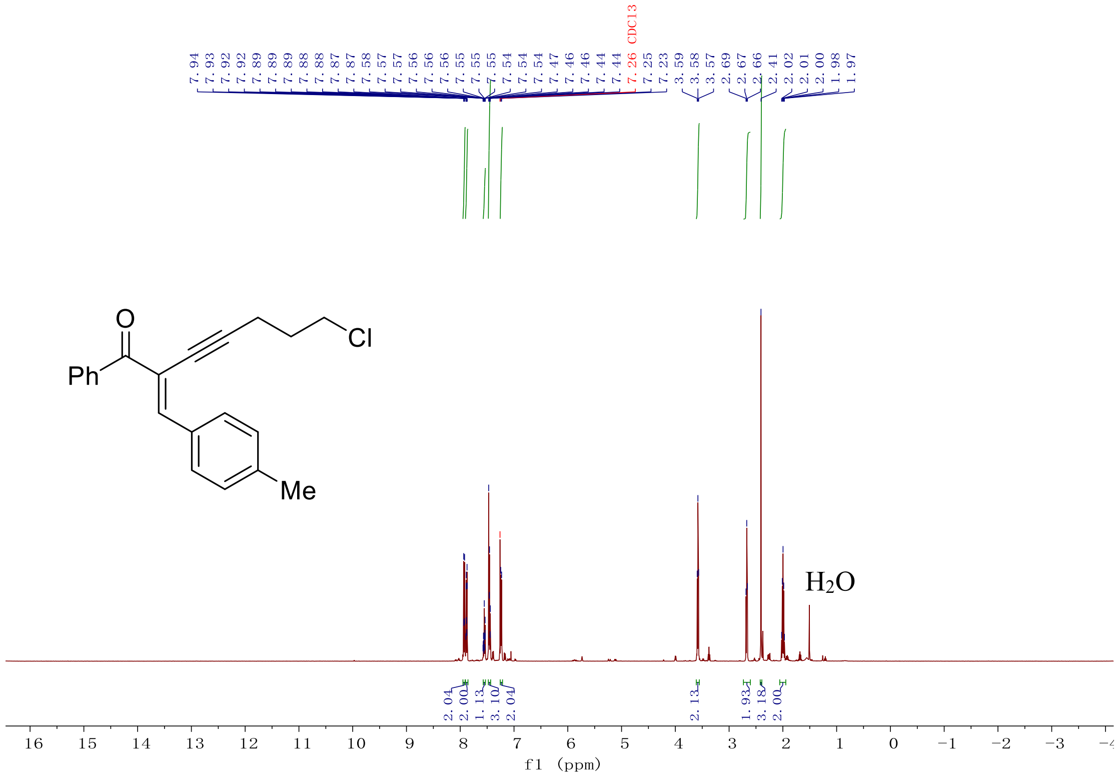

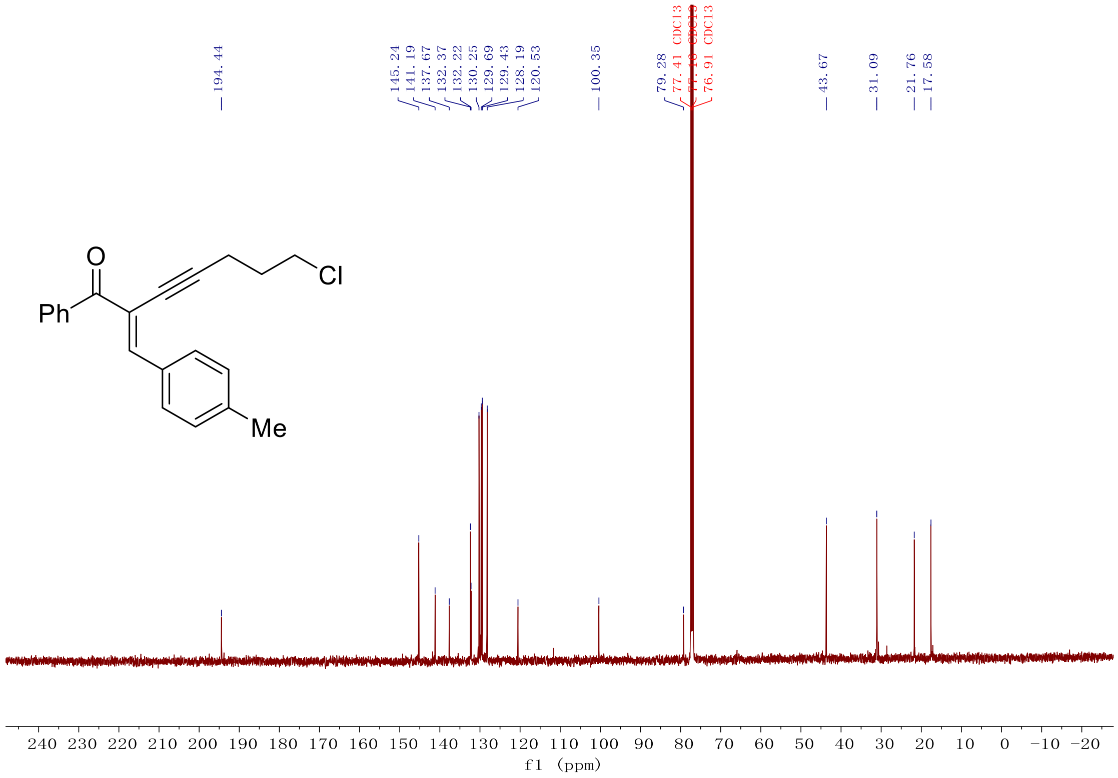

2-(4-Methylbenzylidene)-1-phenyloct-3-yn-1-one (1w)

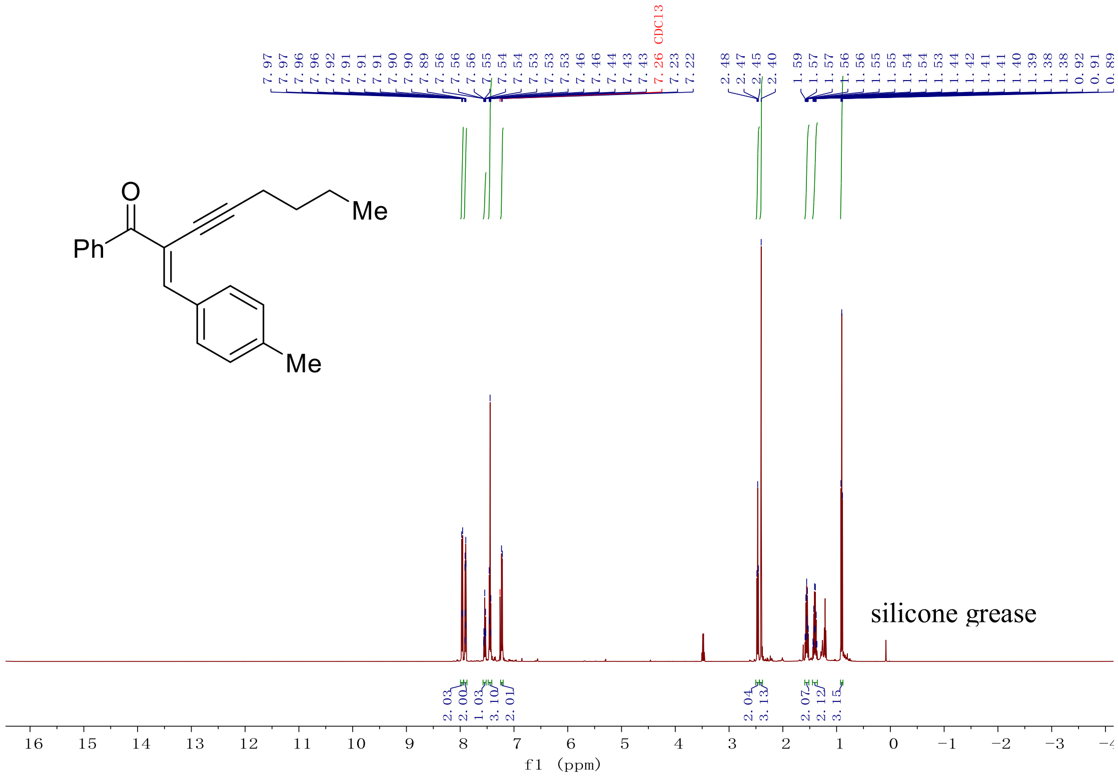

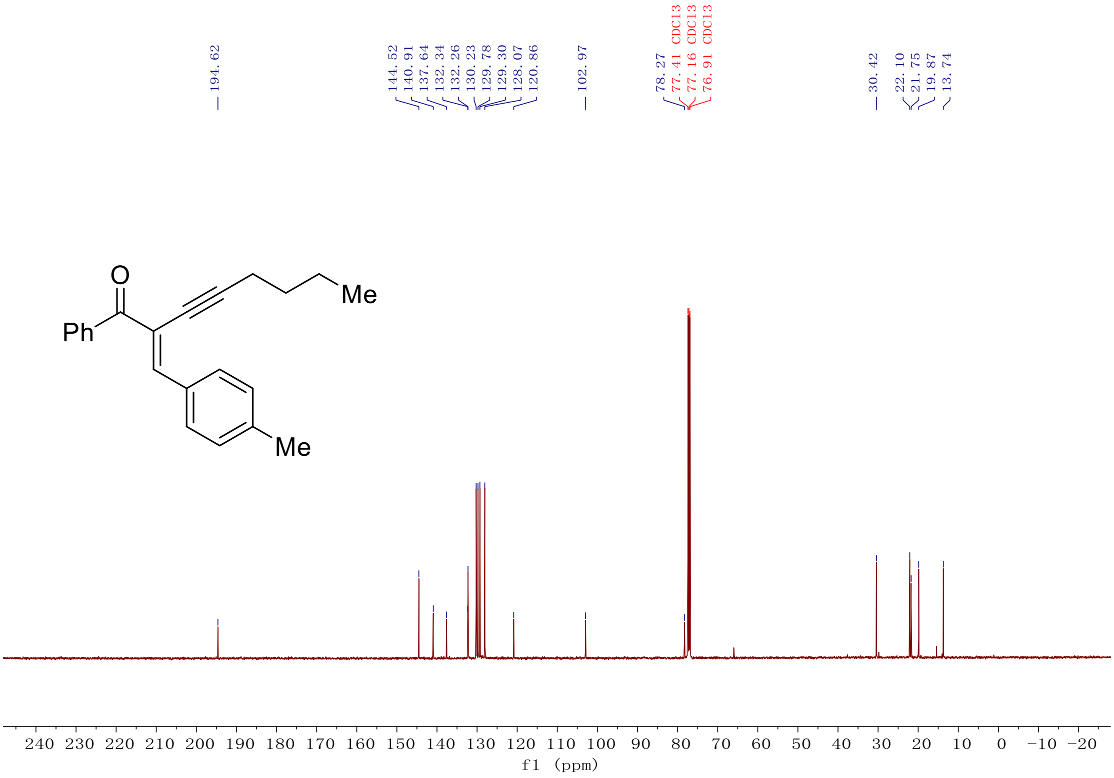

3-Benzyl-2,5-diphenylfuran (3a)

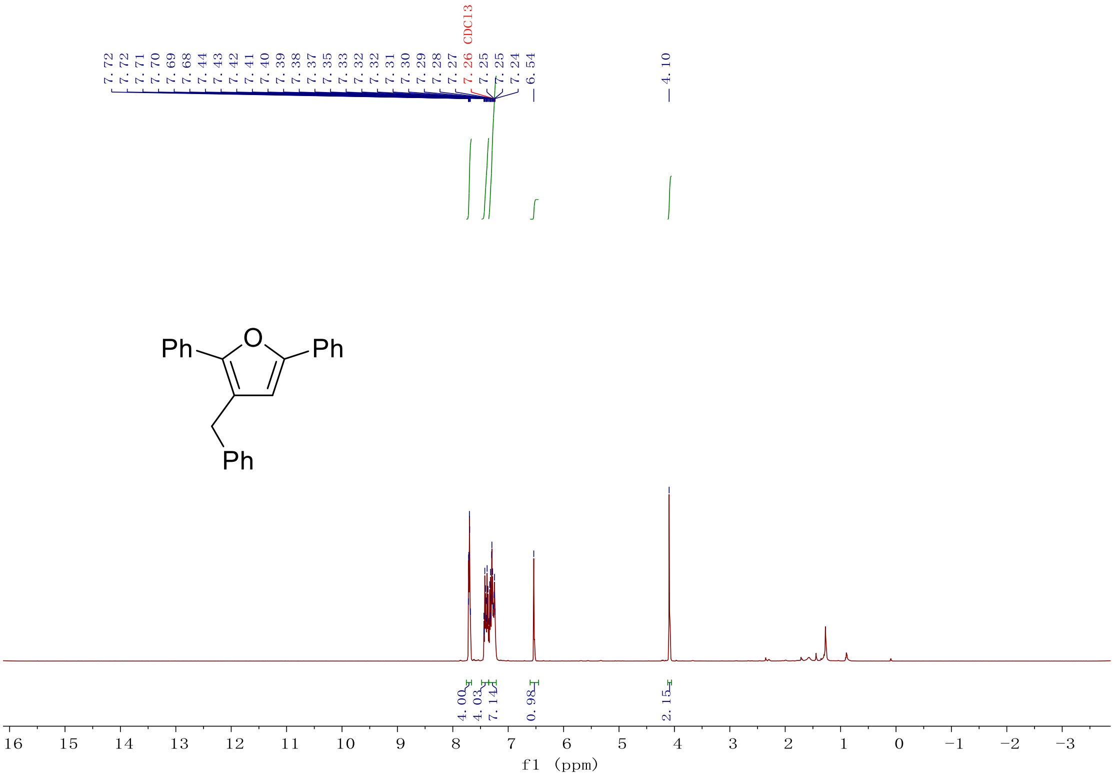

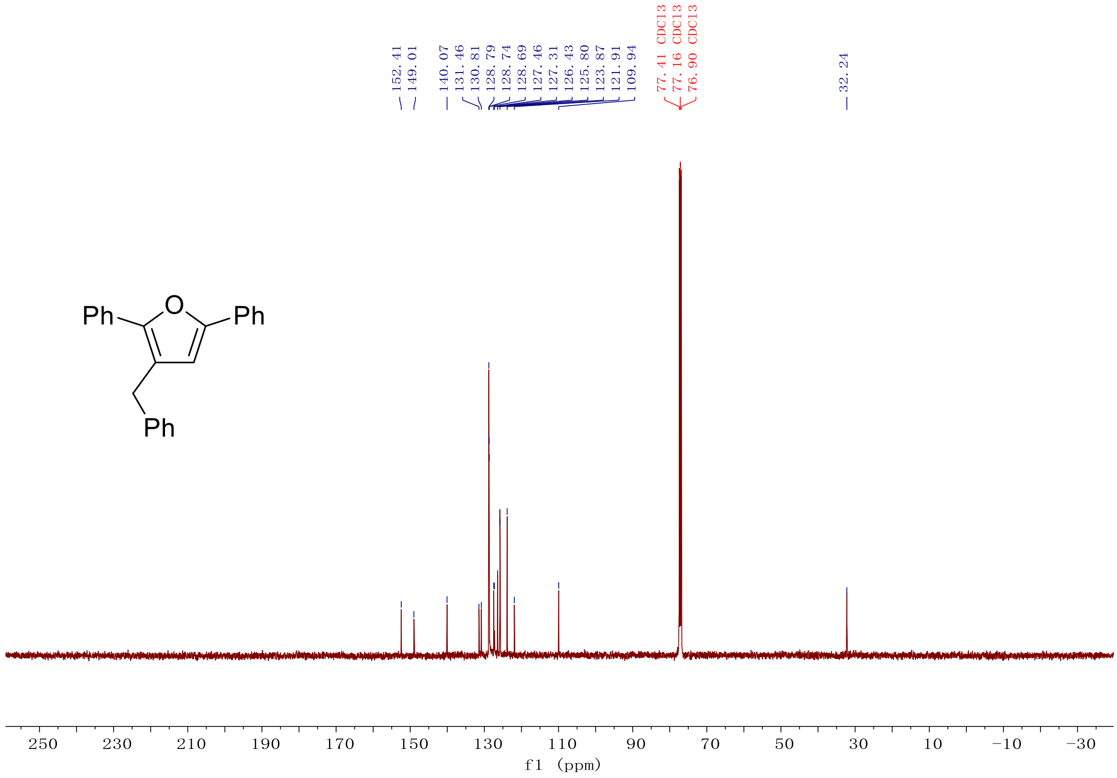

3-Benzyl-2-(4-methoxyphenyl)-5-phenylfuran (3b)

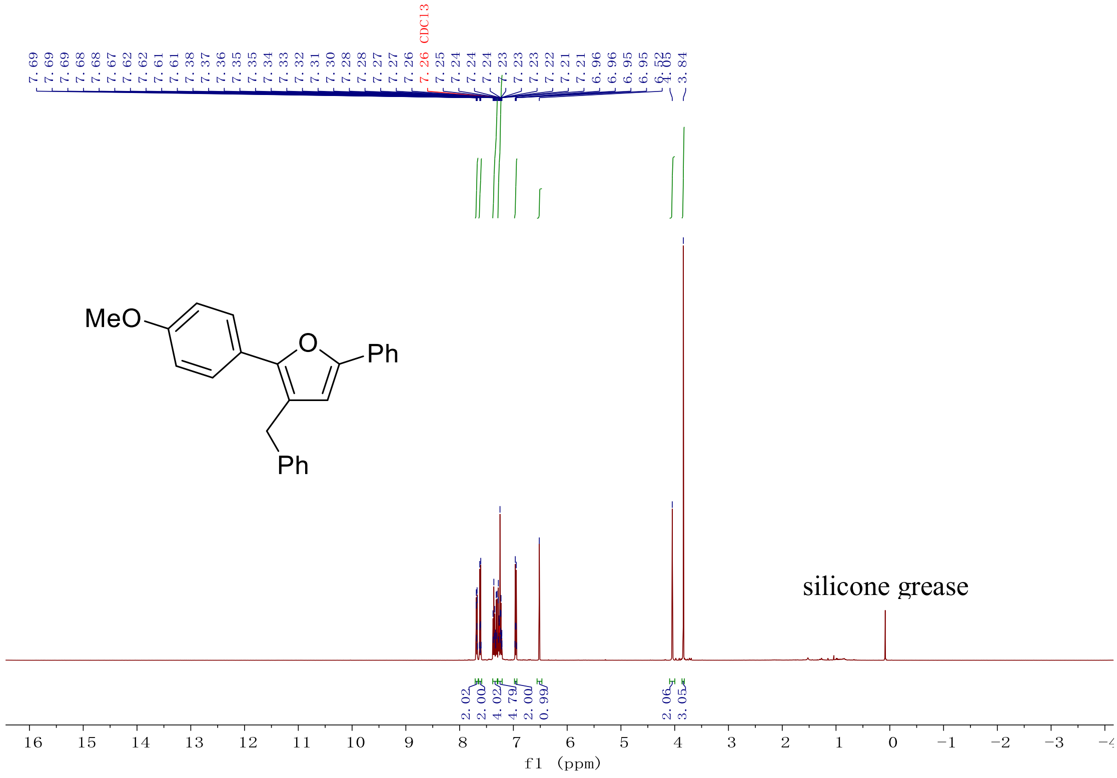

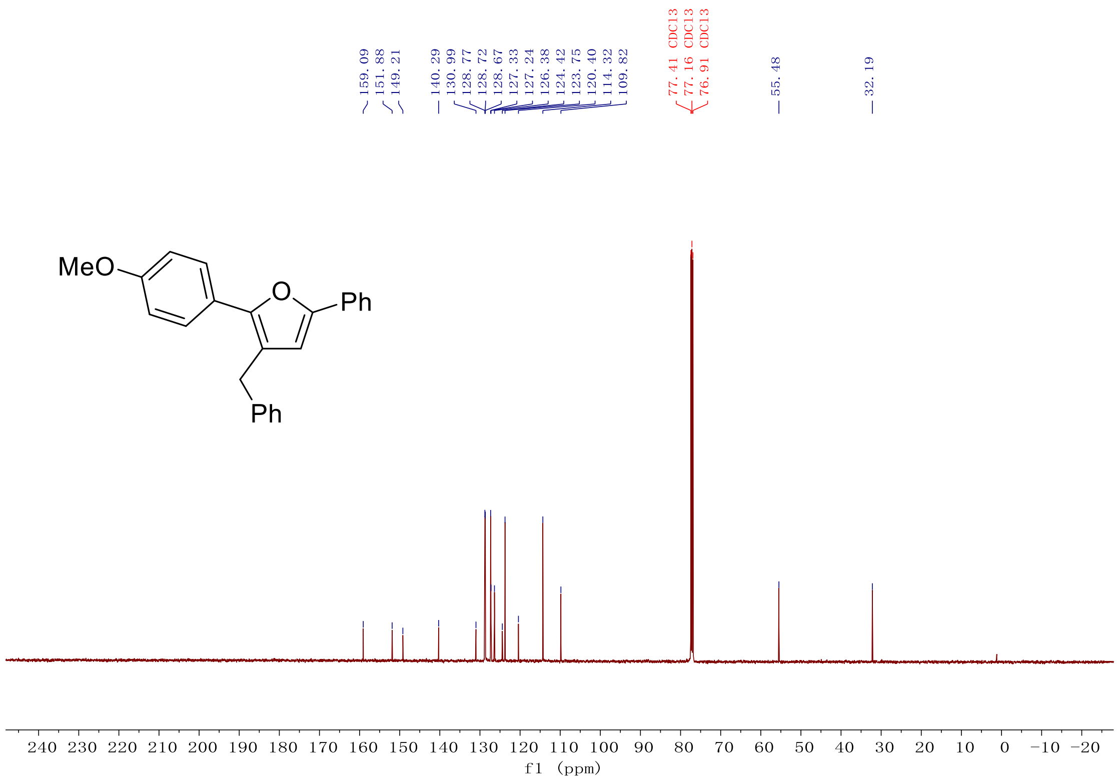

3-Benzyl-2-(4-fluorophenyl)-5-phenylfuran (3c)

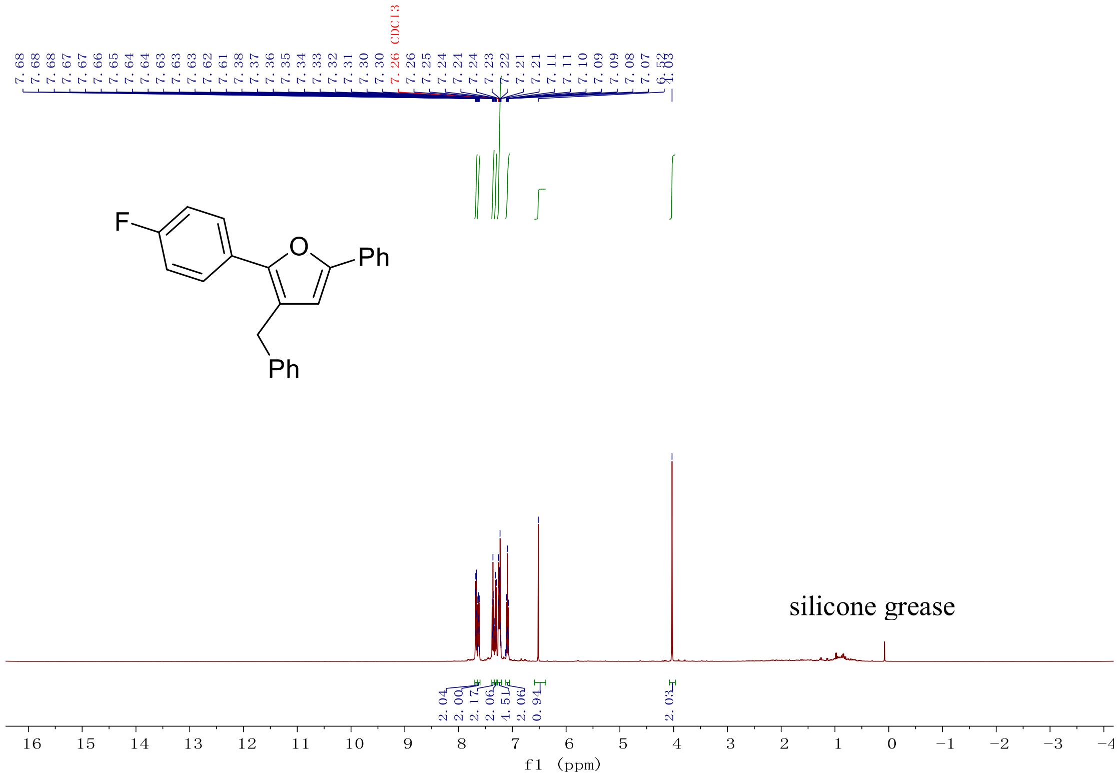

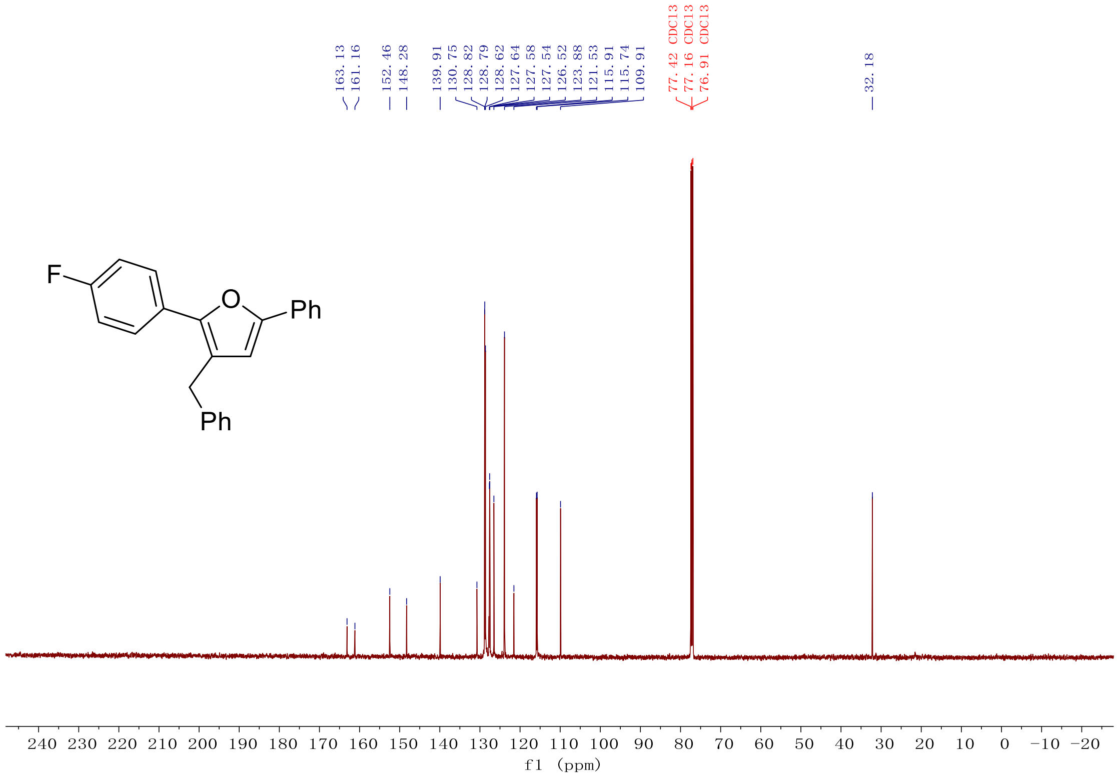
163.13
161.16
152.46
148.28
139.91
130.75
128.82
128.79
128.62
127.64
127.58
127.54
126.52
123.88
121.53
115.91
115.74
109.91
77.42 CDCl3
77.16 CDCl3
76.91 CDCl3
32.18
F
O
Ph
Ph
240 230 220 210 200 190 180 170 160 150 140 130 120 110 100 90 80 70 60 50 40 30 20 10 0 −10 −20
f1 (ppm)

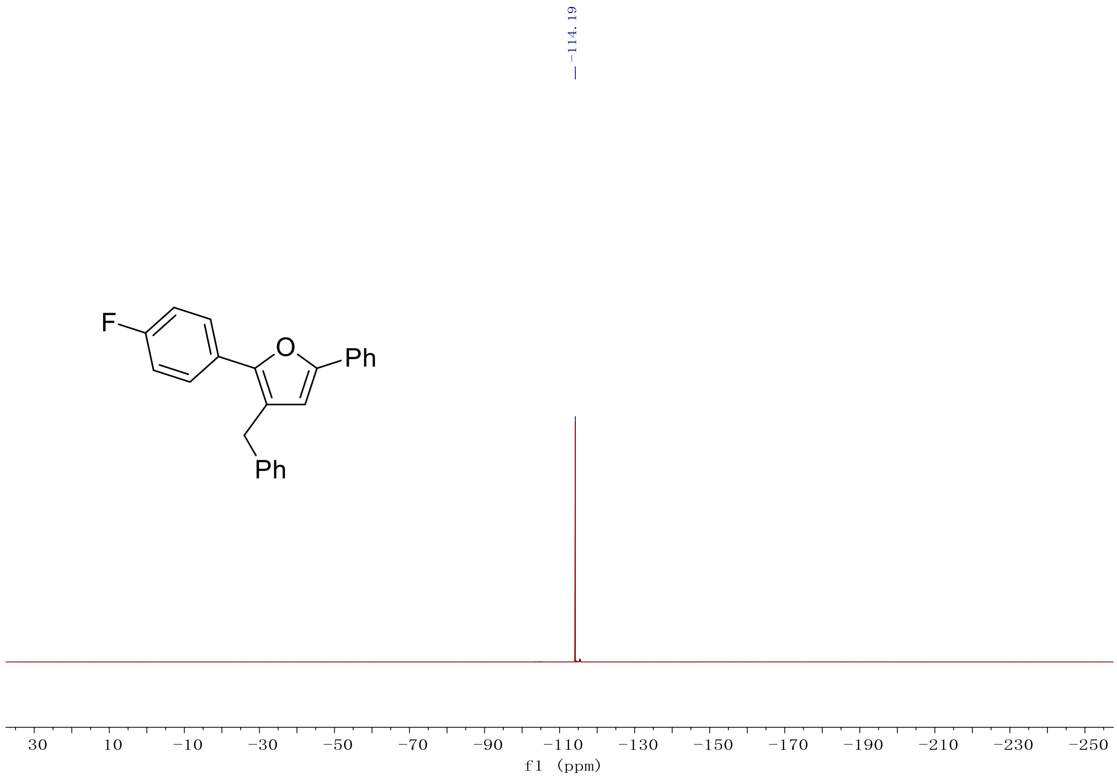
−114.19
F
O
Ph
Ph
30 10 −10 −30 −50 −70 −90 −110 −130 −150 −170 −190 −210 −230 −250
f1 (ppm)

2-(4-Fluorophenyl)-3-(4-methoxybenzyl)-5-phenylfuran (3d)

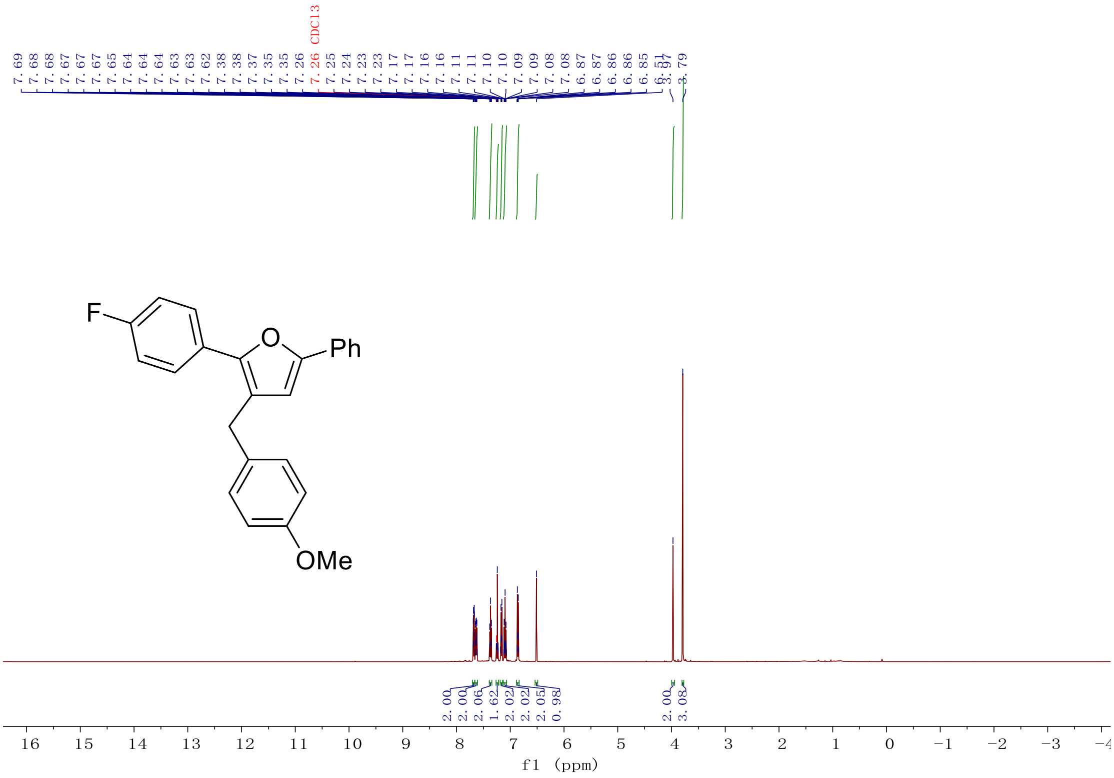

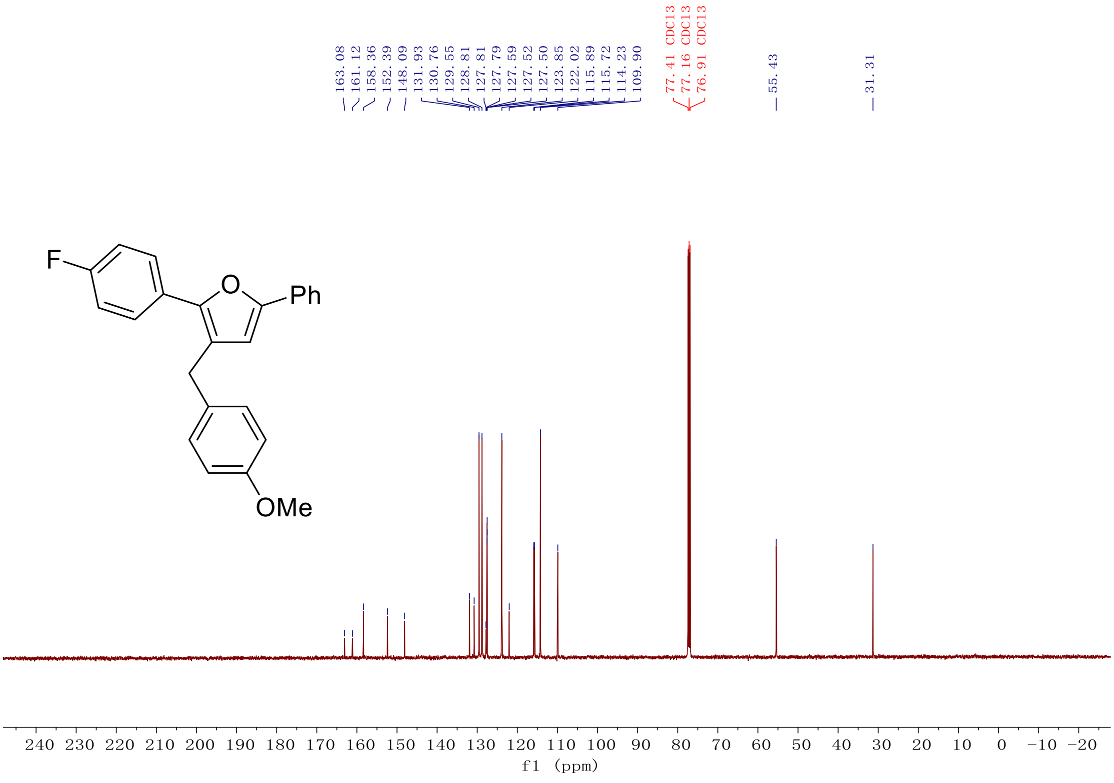

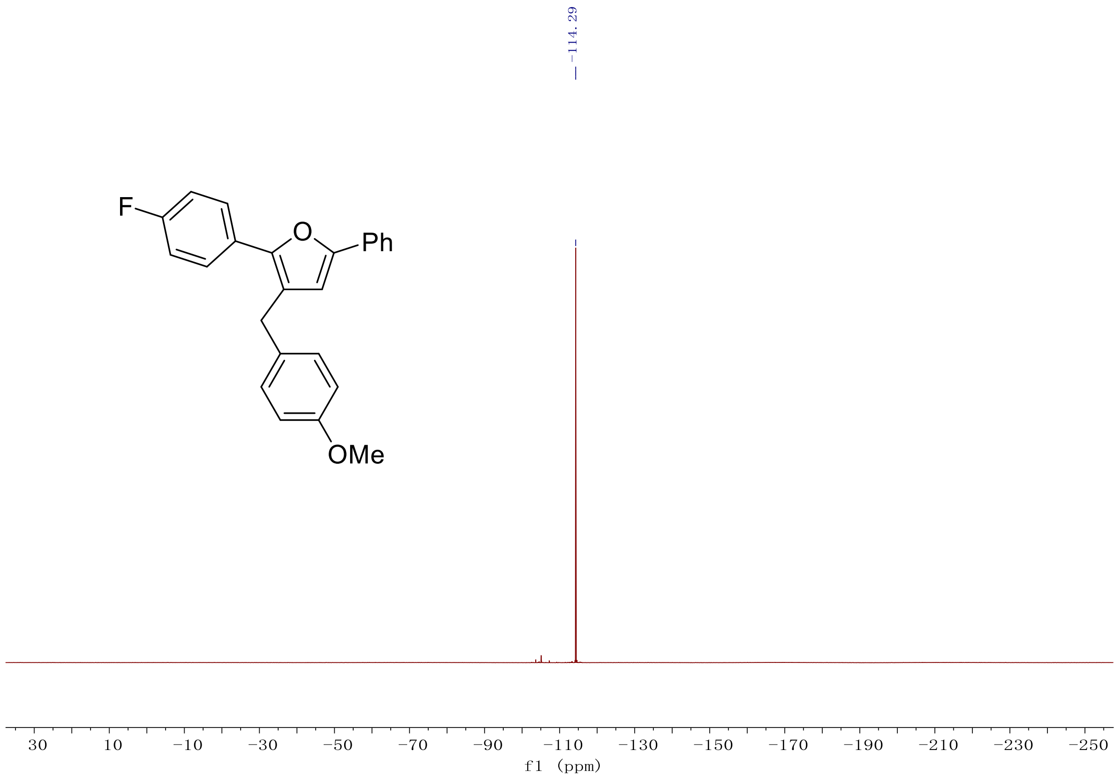

3-(4-Methoxybenzyl)-2,5-diphenylfuran (3e)

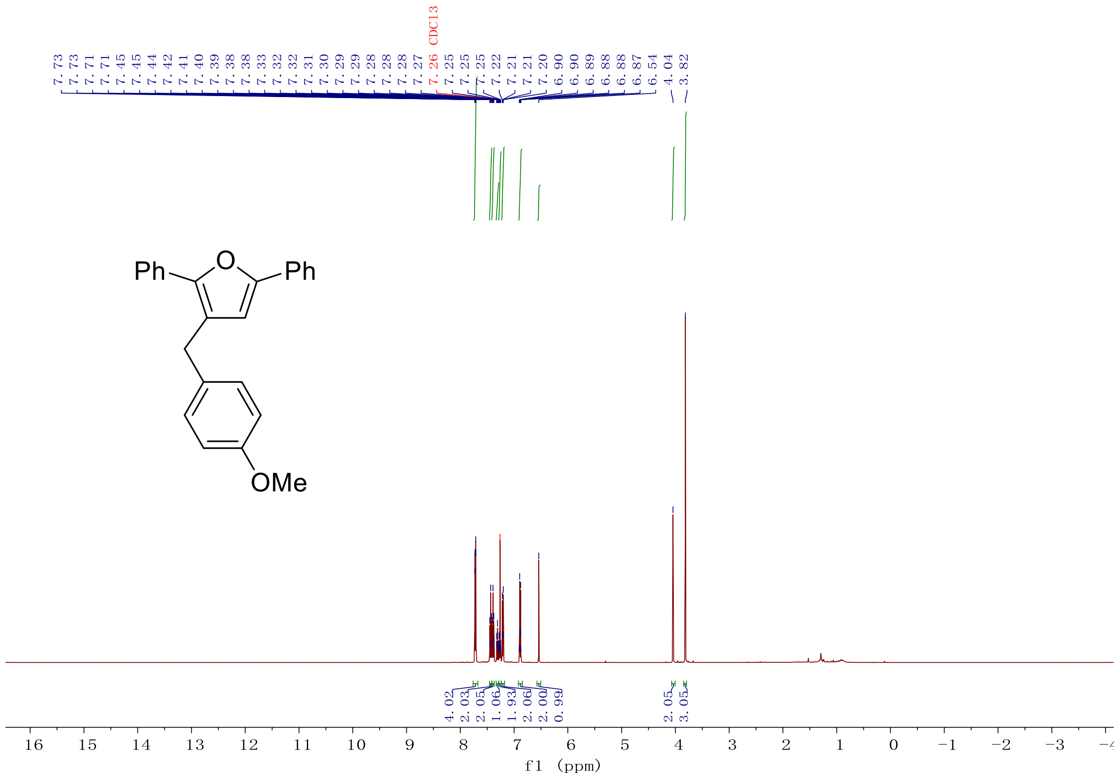

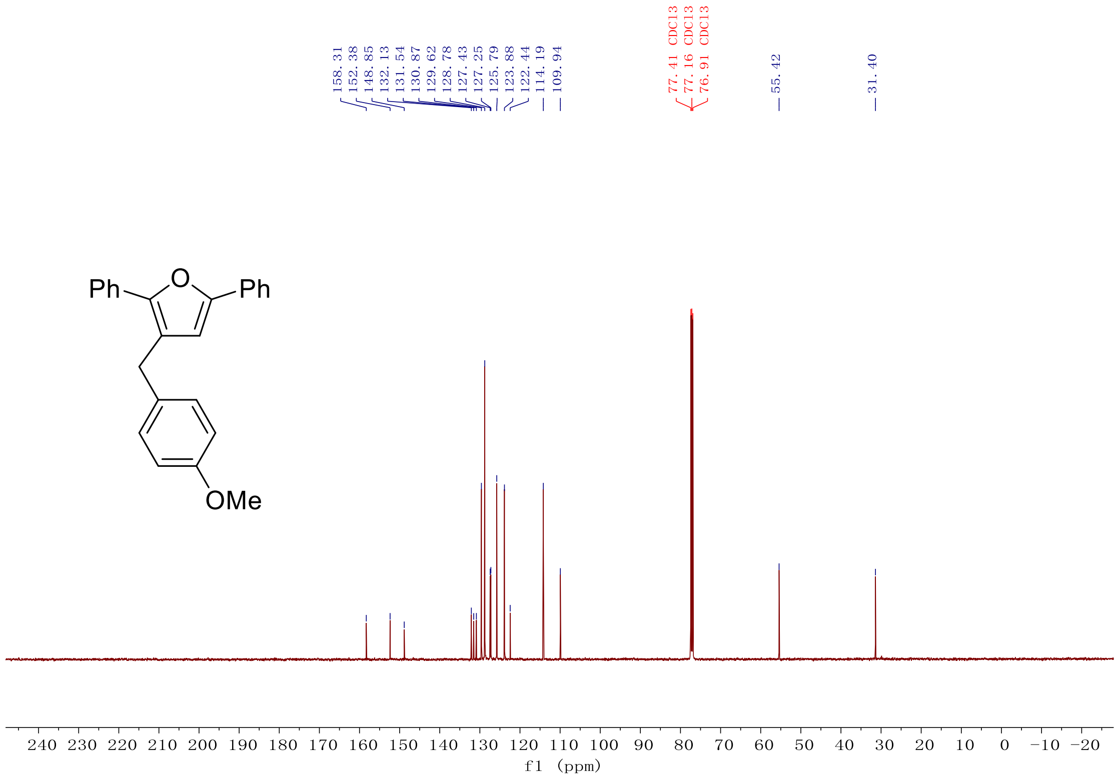

3-(4-Methylbenzyl)-2,5-diphenylfuran (3f)

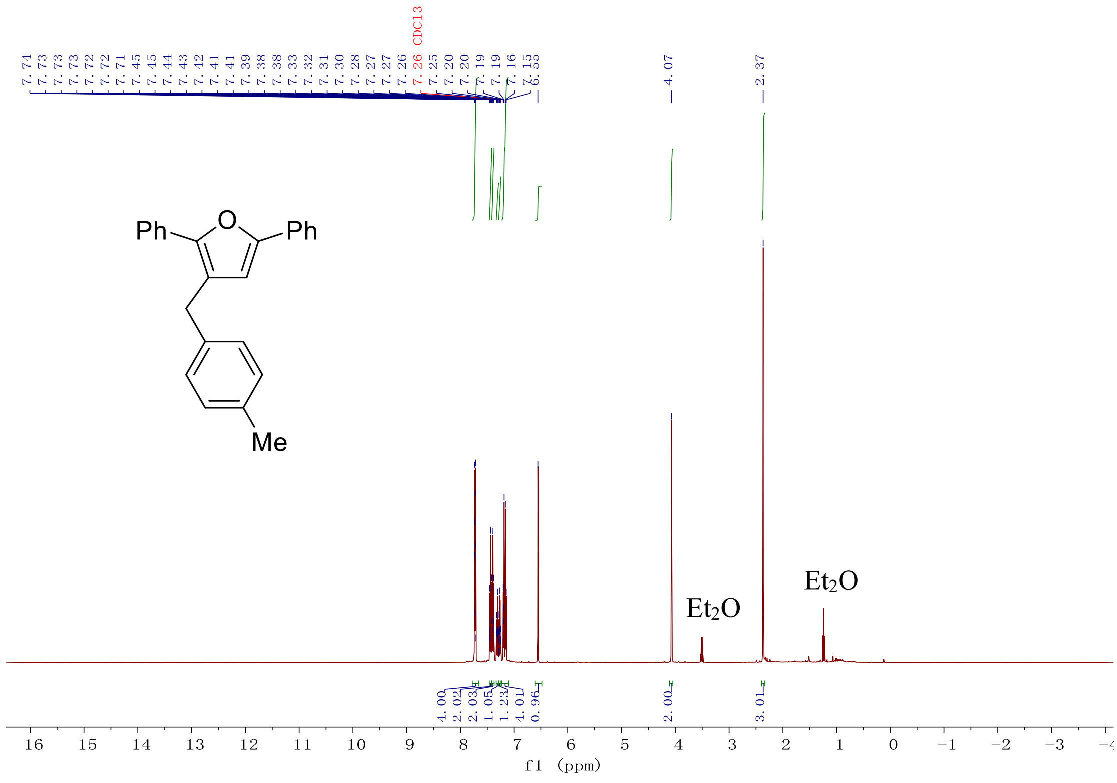

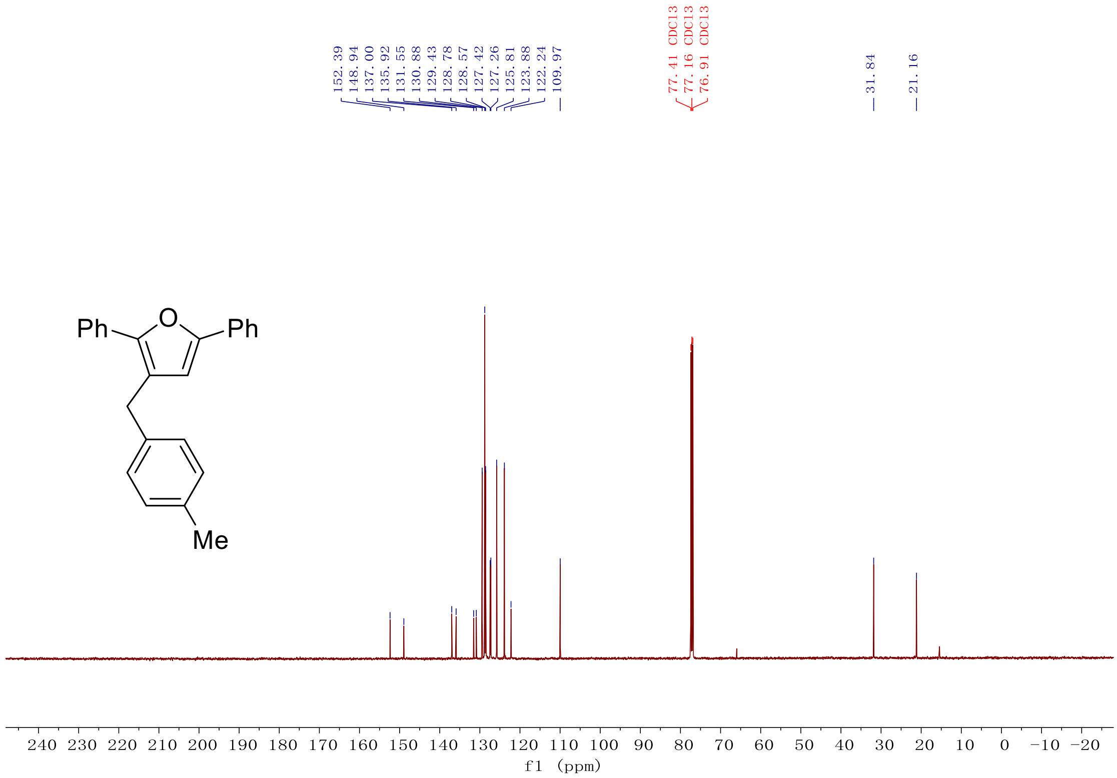

3-(4-Chlorobenzyl)-2,5-diphenylfuran (3g)

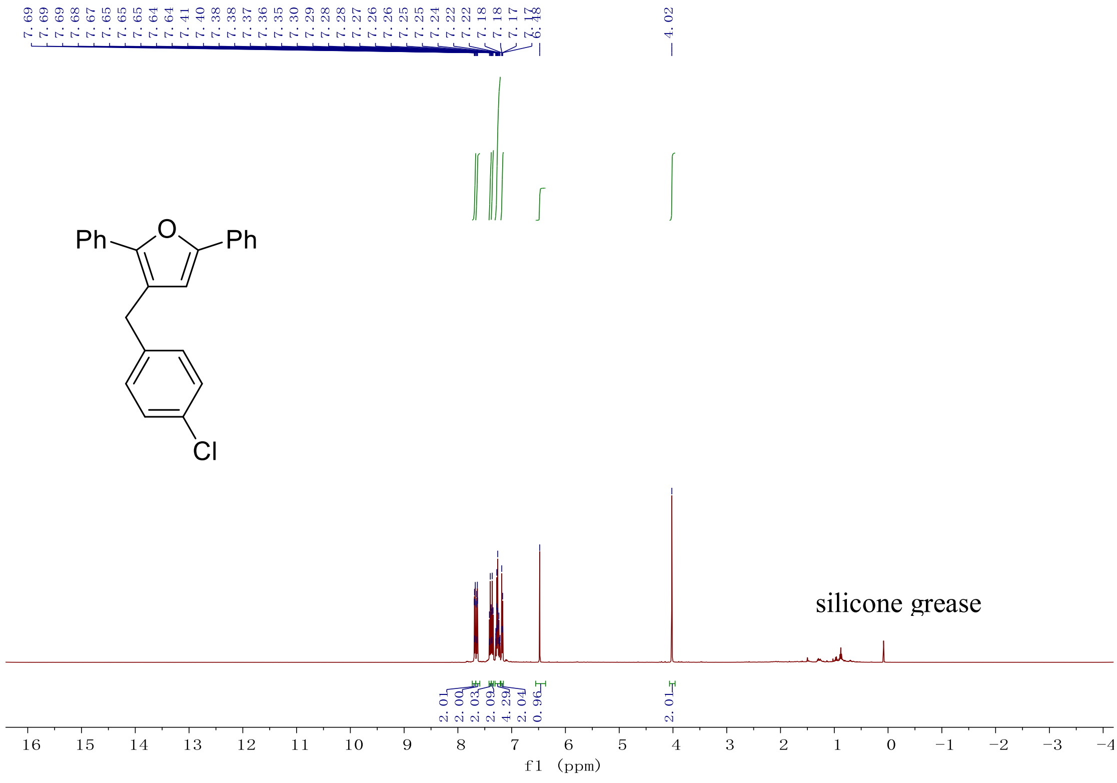

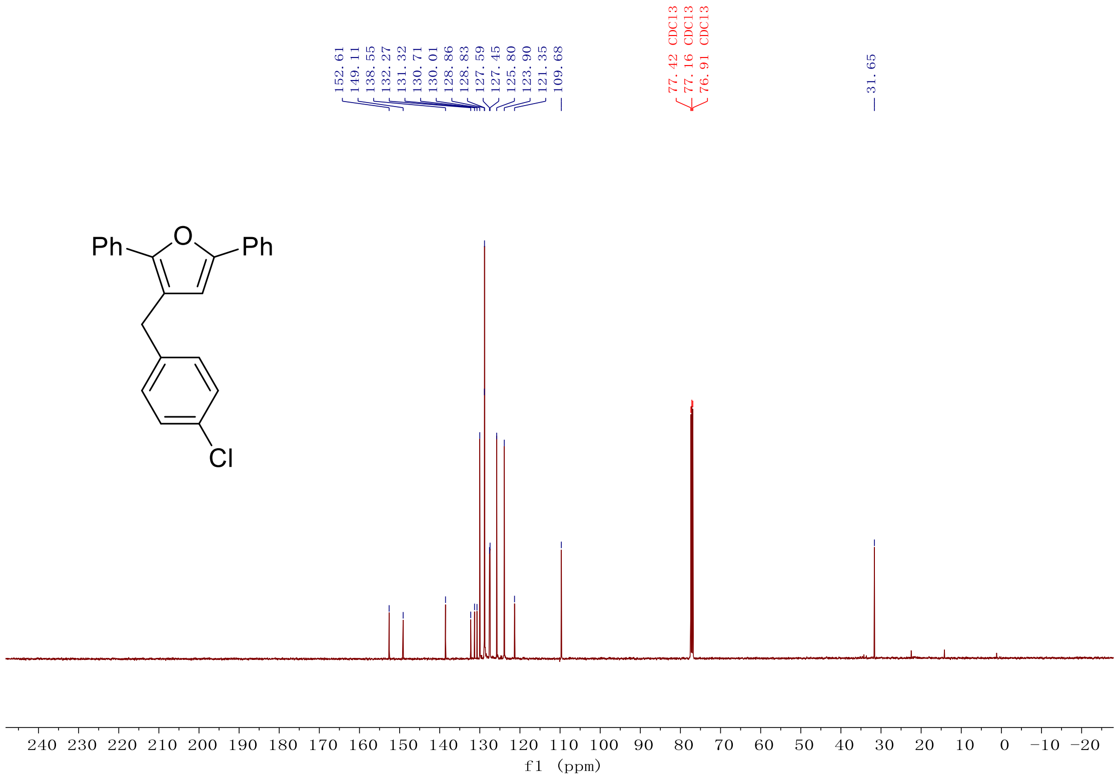

2,5-Diphenyl-3-(thiophen-2-ylmethyl)furan (3h)

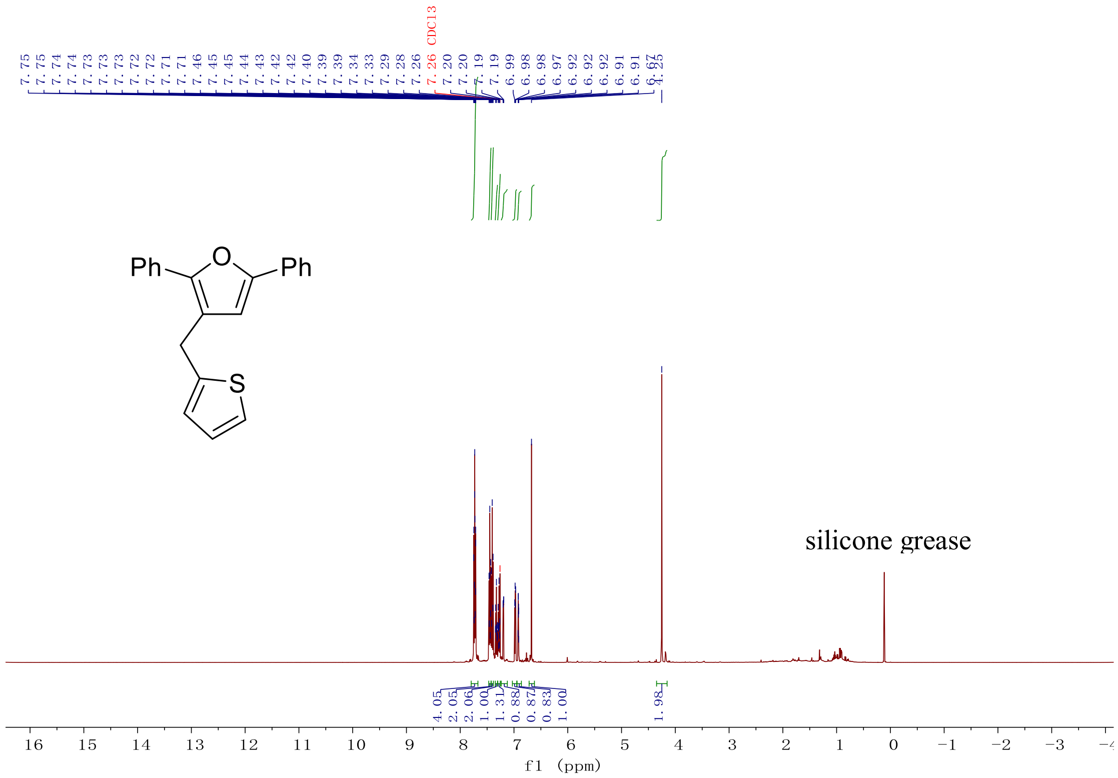

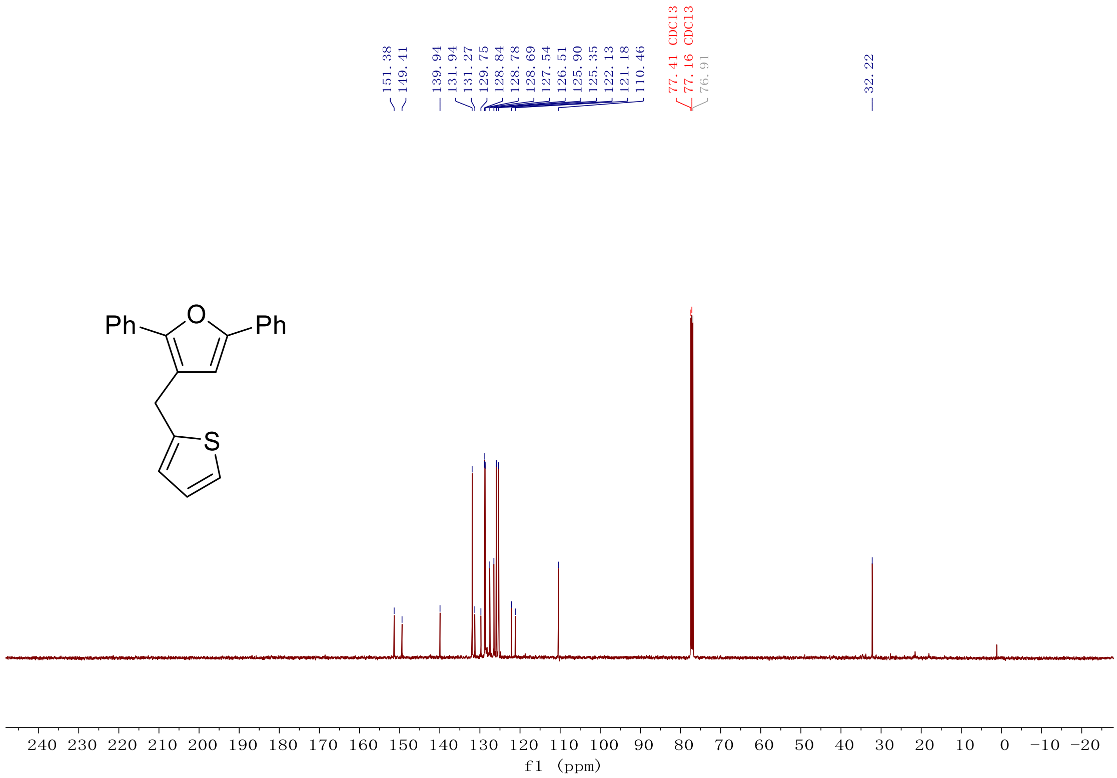

3-Benzyl-2-phenyl-5-(4-(trifluoromethyl)phenyl)furan (3i)

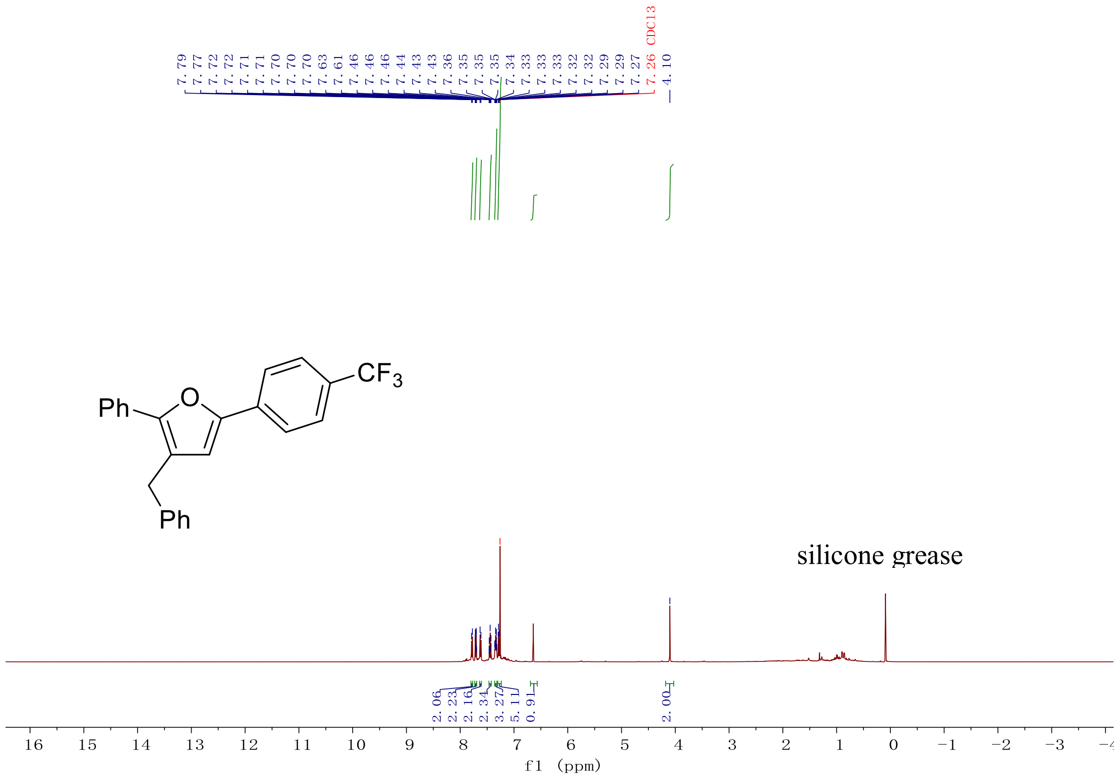

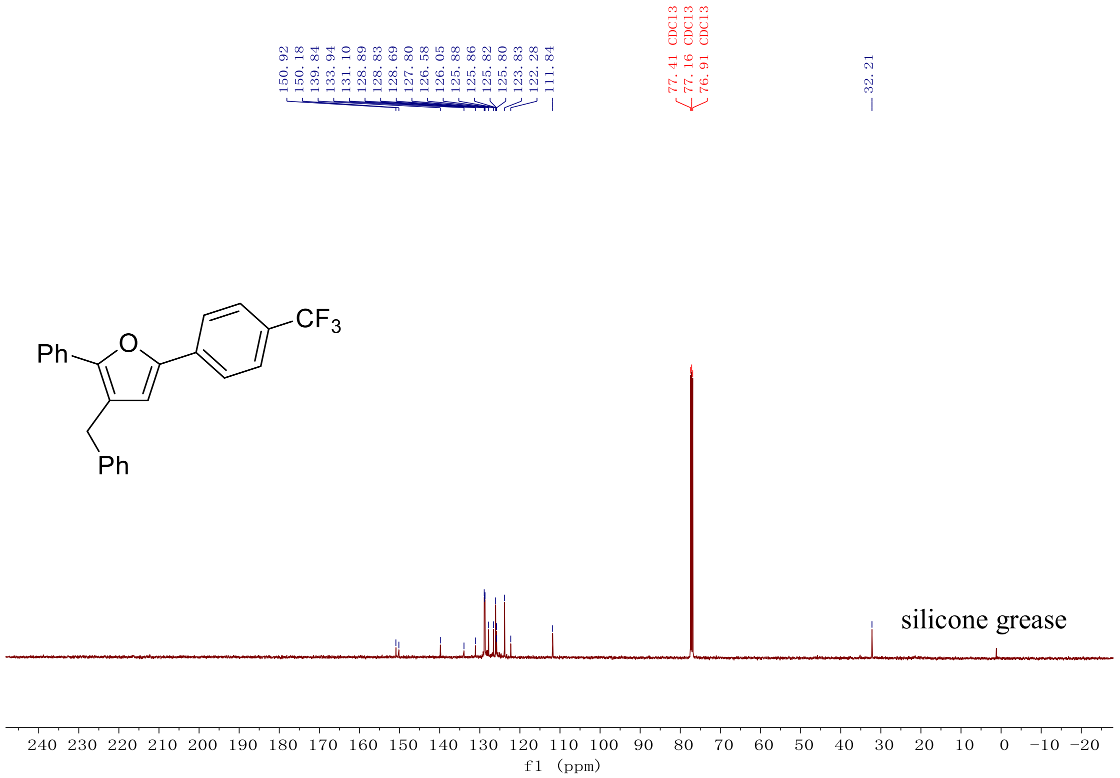
150.92
150.18
139.84
133.94
131.10
128.89
128.83
128.69
127.80
126.58
126.05
125.88
125.86
125.82
125.80
123.83
122.28
111.84
77.41 CDCl3
77.16 CDCl3
76.91 CDCl3
32.21
CF3
Ph
O
Ph
silicone grease
240 230 220 210 200 190 180 170 160 150 140 130 120 110 100 90 80 70 60 50 40 30 20 10 0 −10 −20
f1 (ppm)

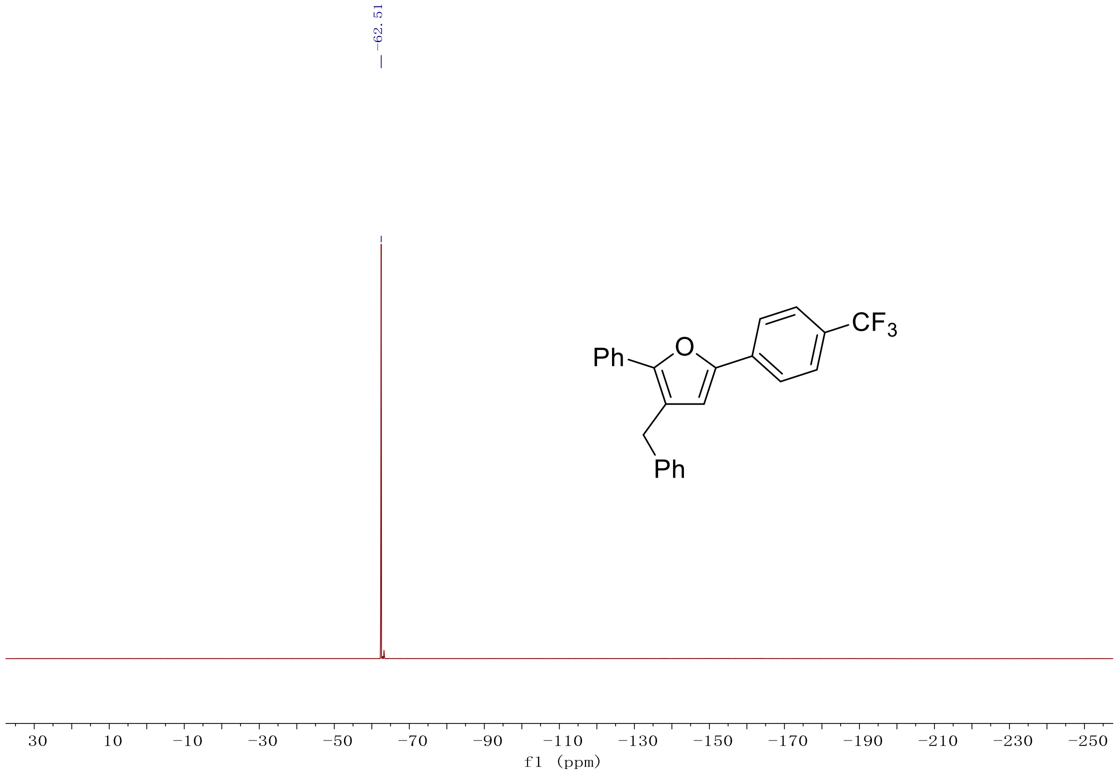
−62.51
CF3
Ph
O
Ph
30 10 −10 −30 −50 −70 −90 −110 −130 −150 −170 −190 −210 −230 −250
f1 (ppm)

3-Benzyl-5-(4-bromophenyl)-2-phenylfuran (3j)

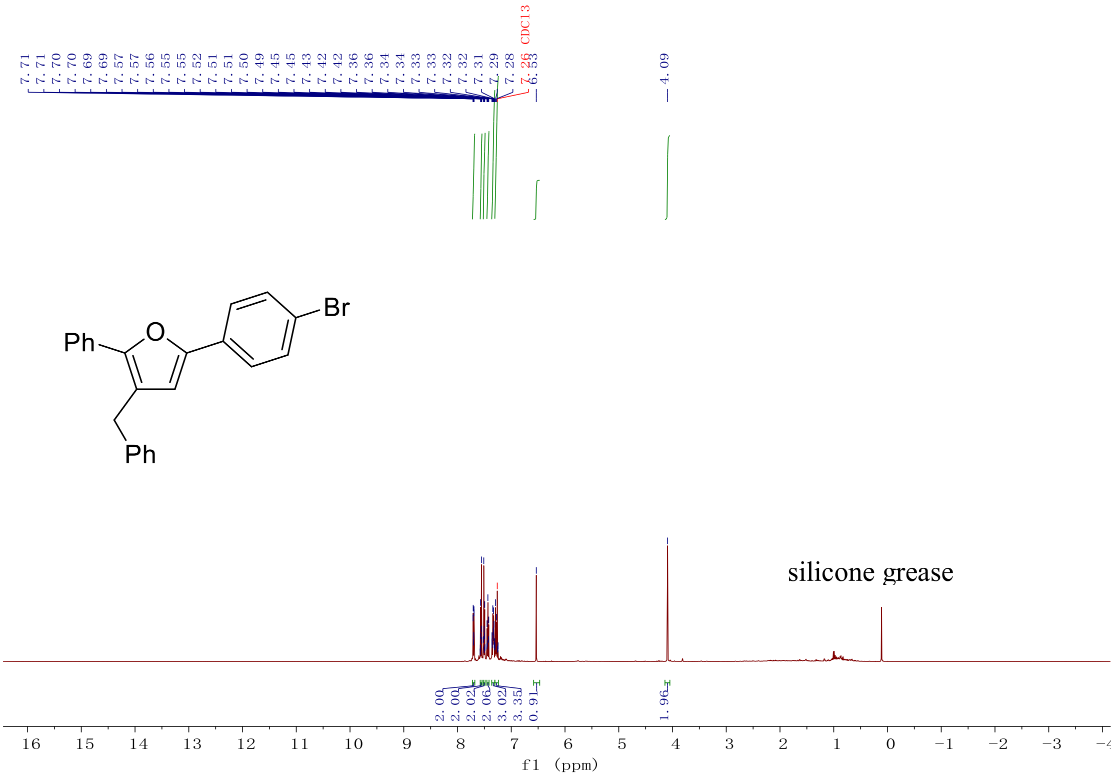

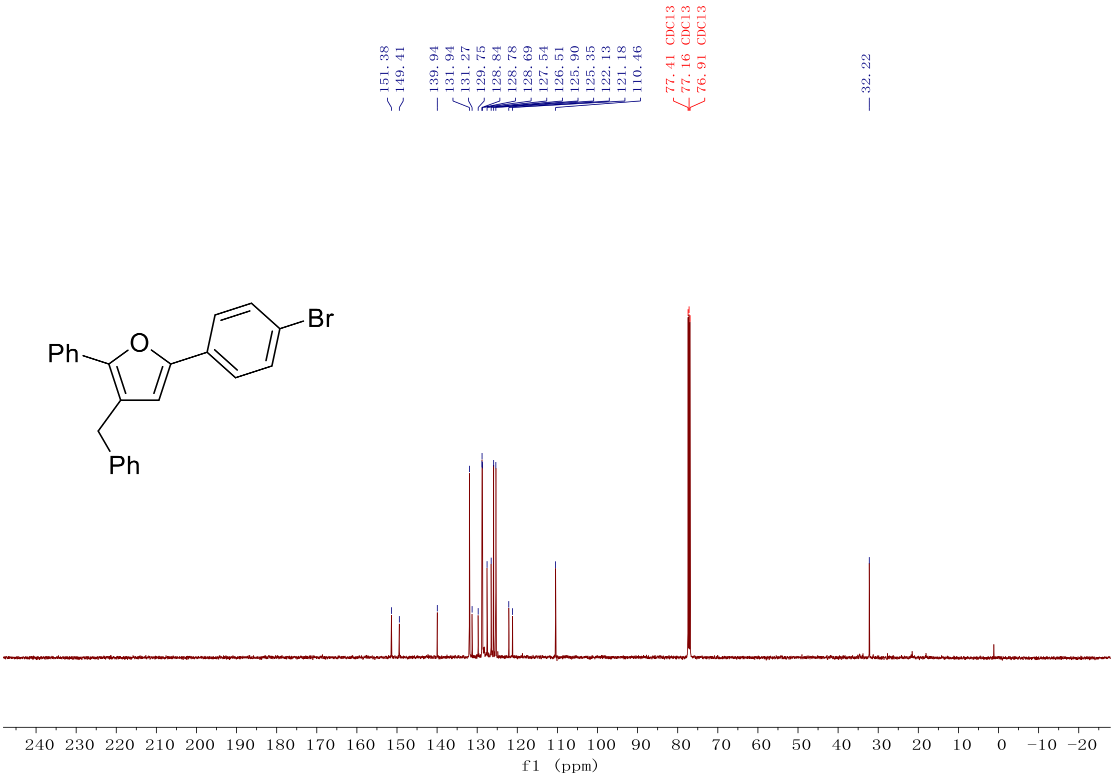

3-Benzyl-2-phenyl-5-(*p*-tolyl)furan (3k)

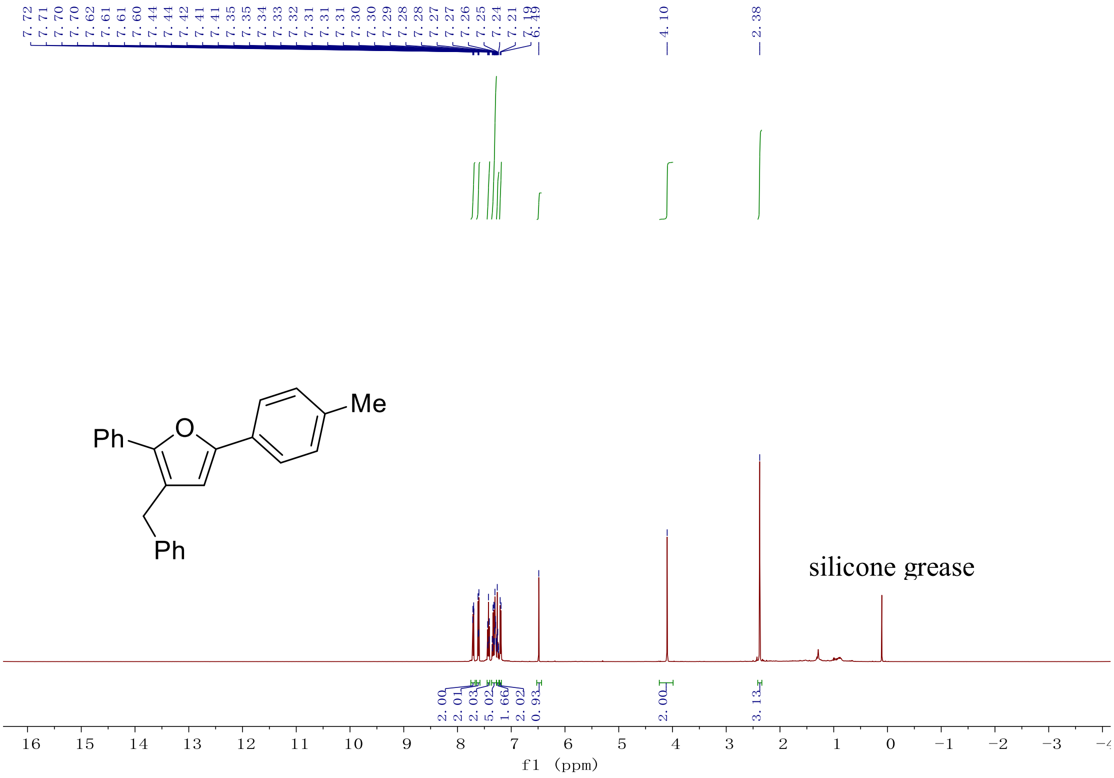

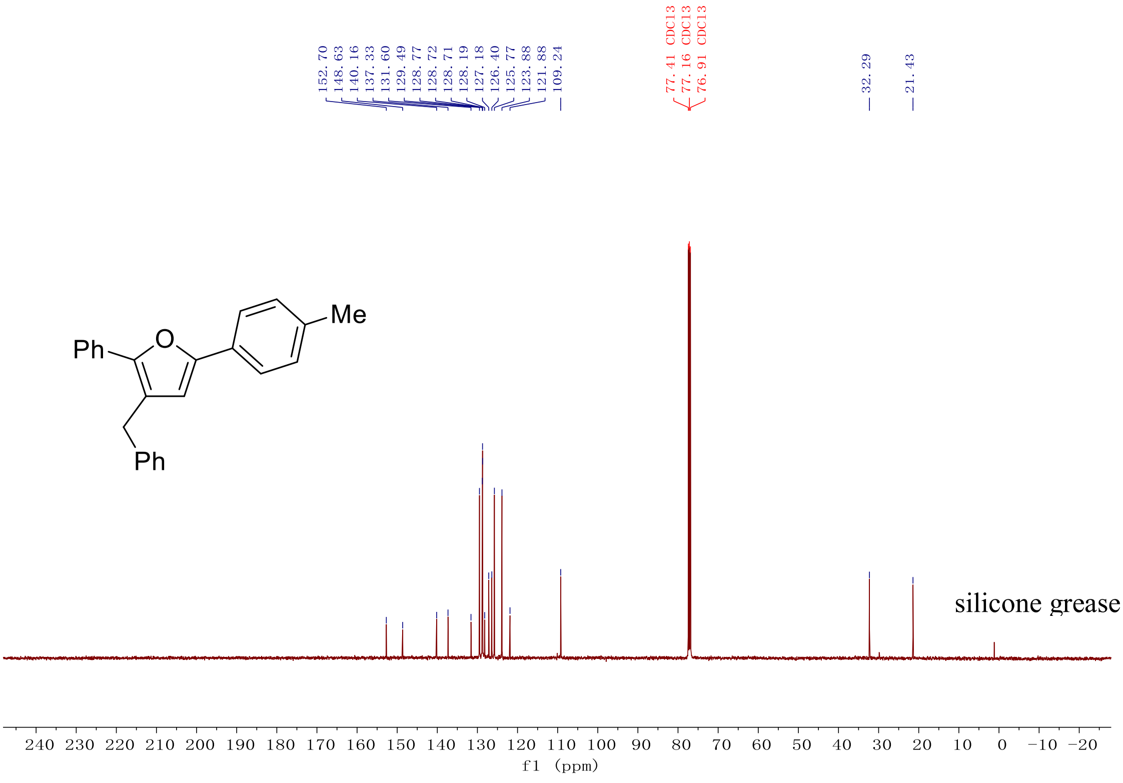

3-Benzyl-2-phenyl-5-(*m*-tolyl)furan (3l)

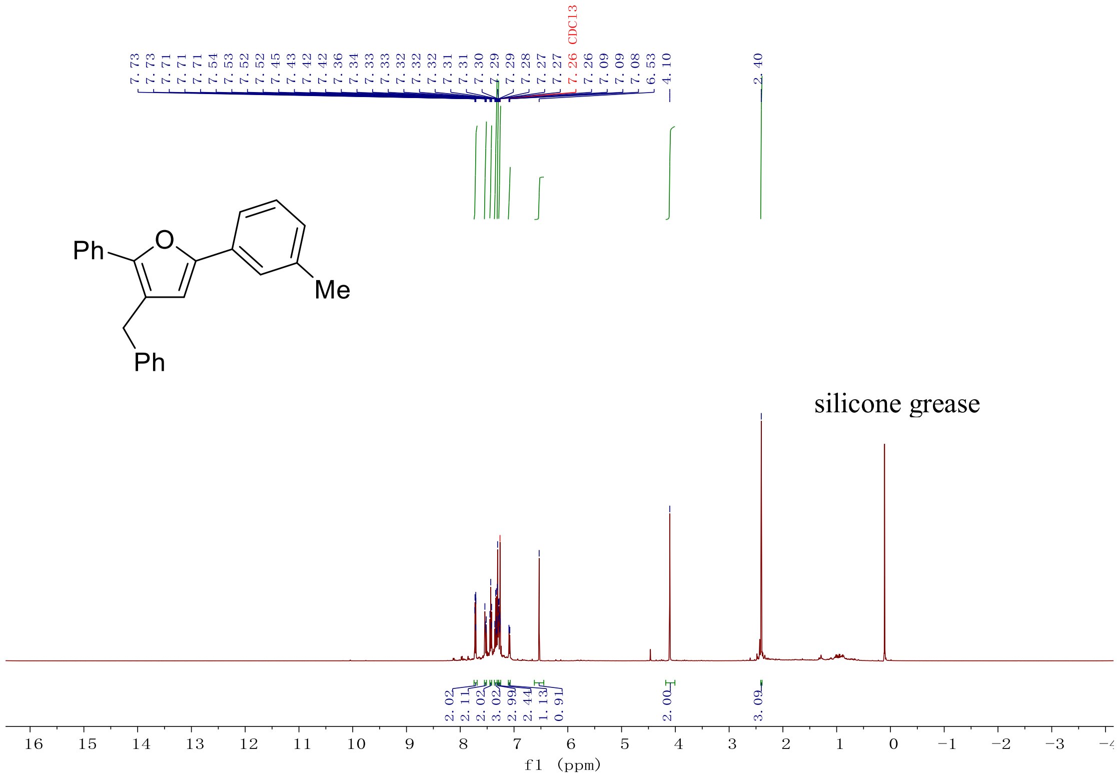

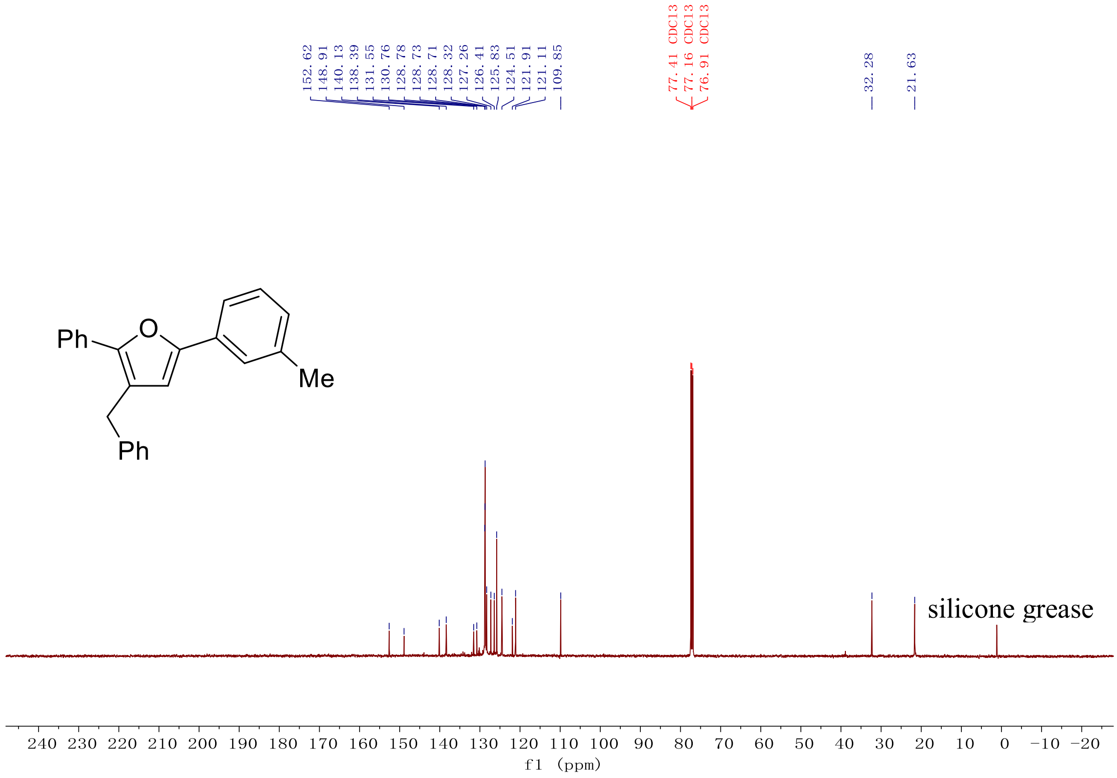

3-Benzyl-5-(4-methoxyphenyl)-2-phenylfuran (3m)

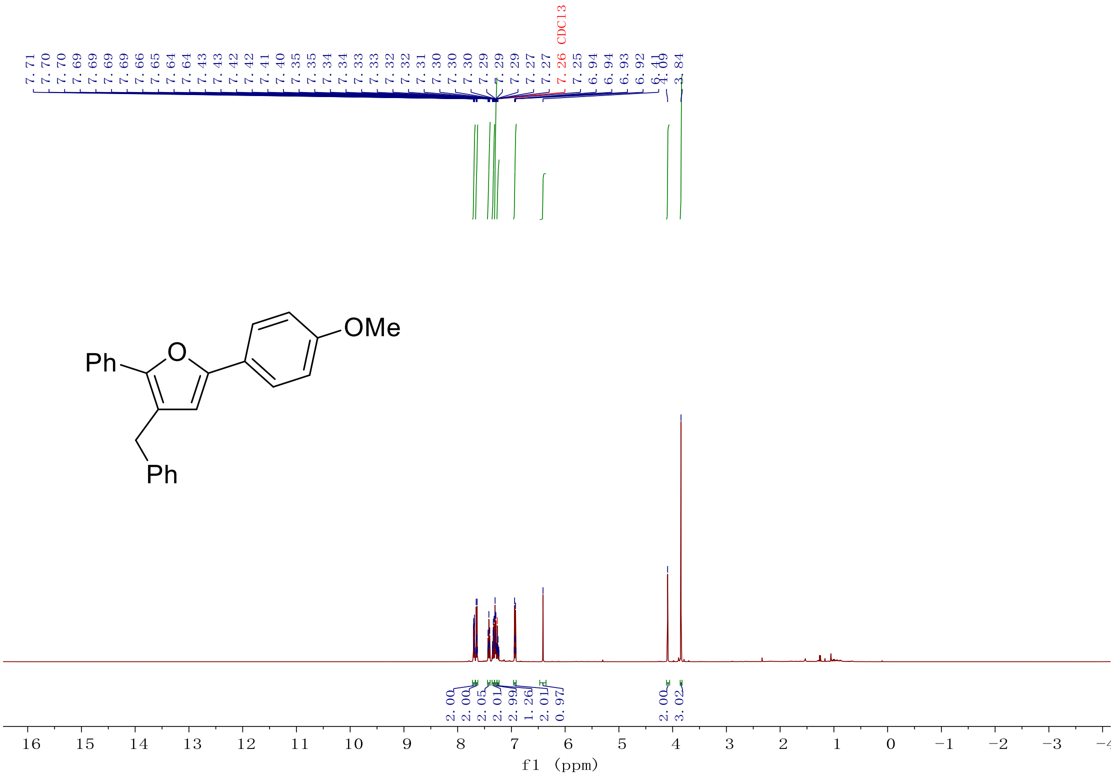

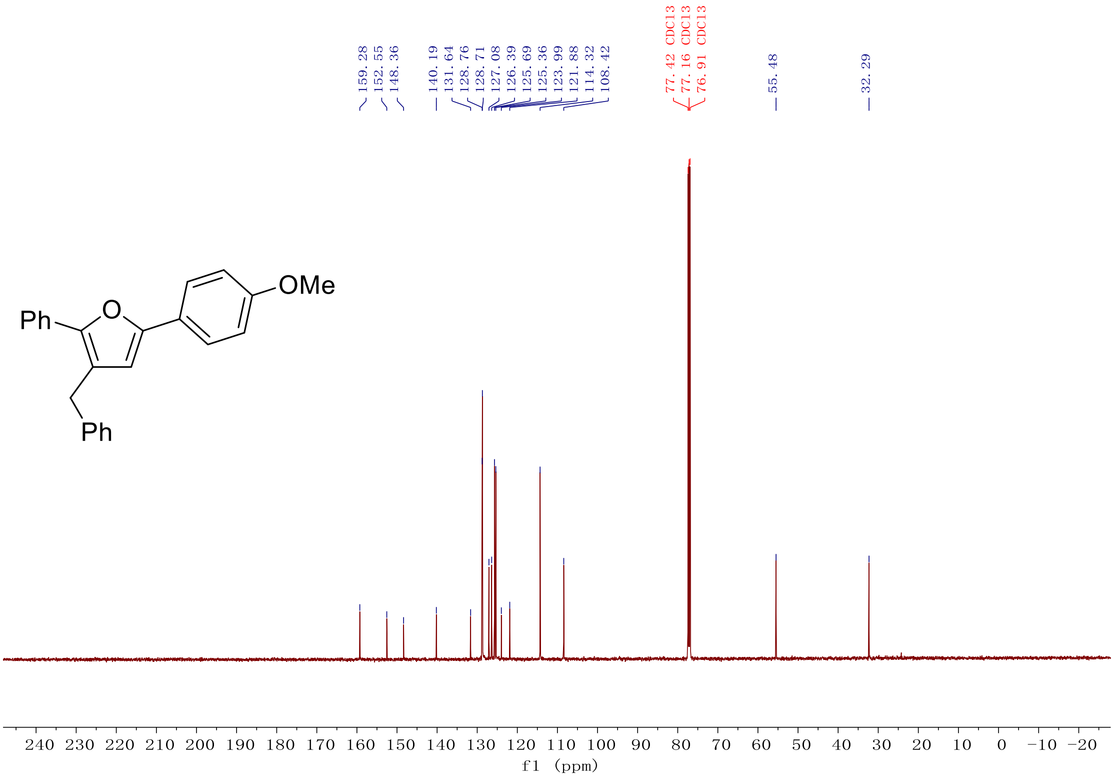

3-Benzyl-2-methyl-5-phenylfuran (3n)

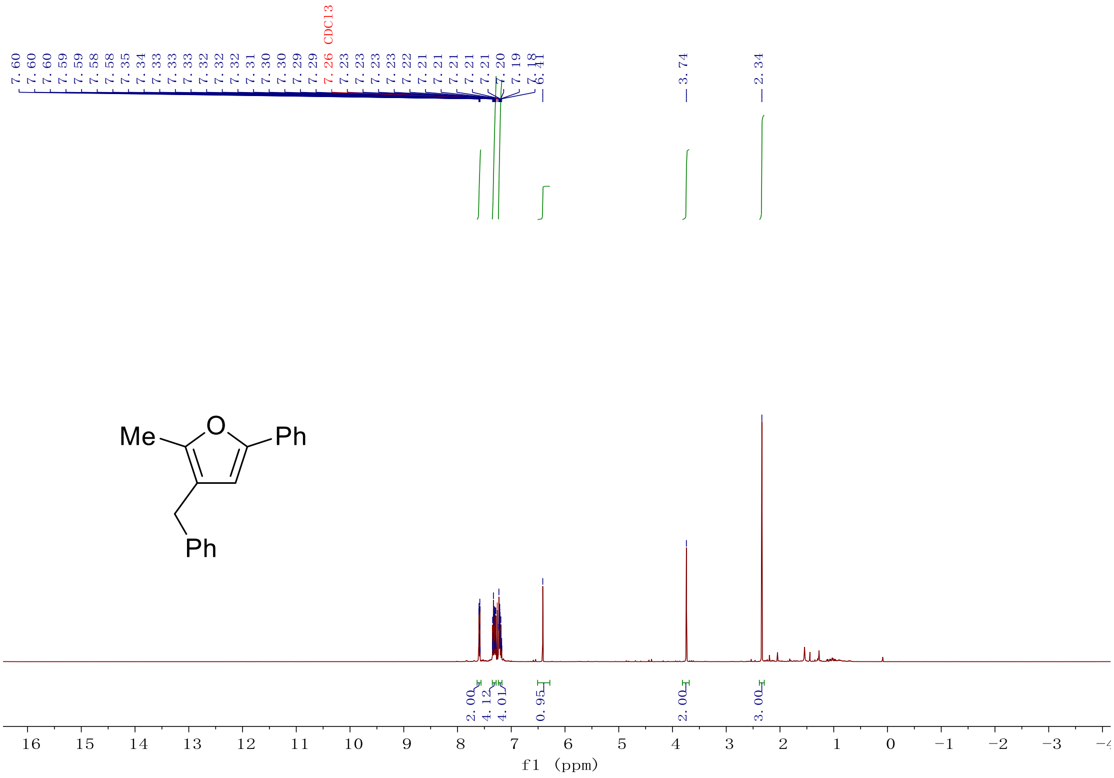

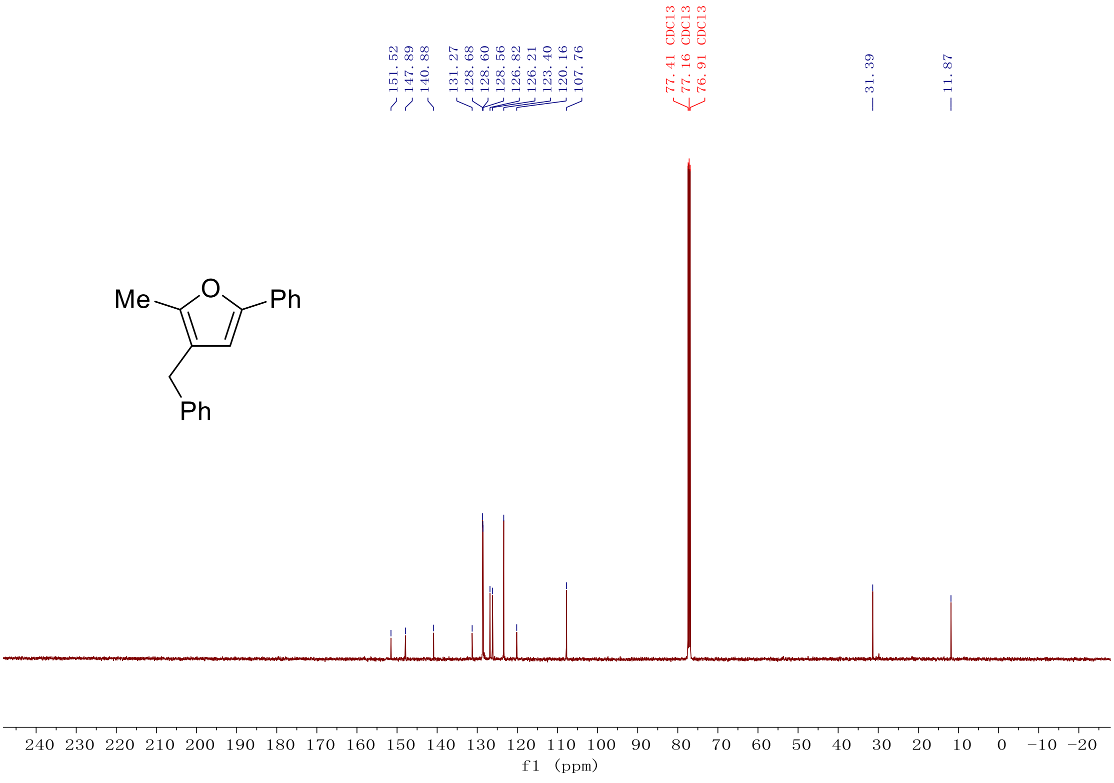

3-Benzyl-5-(4-methoxyphenyl)-2-methylfuran (3o)

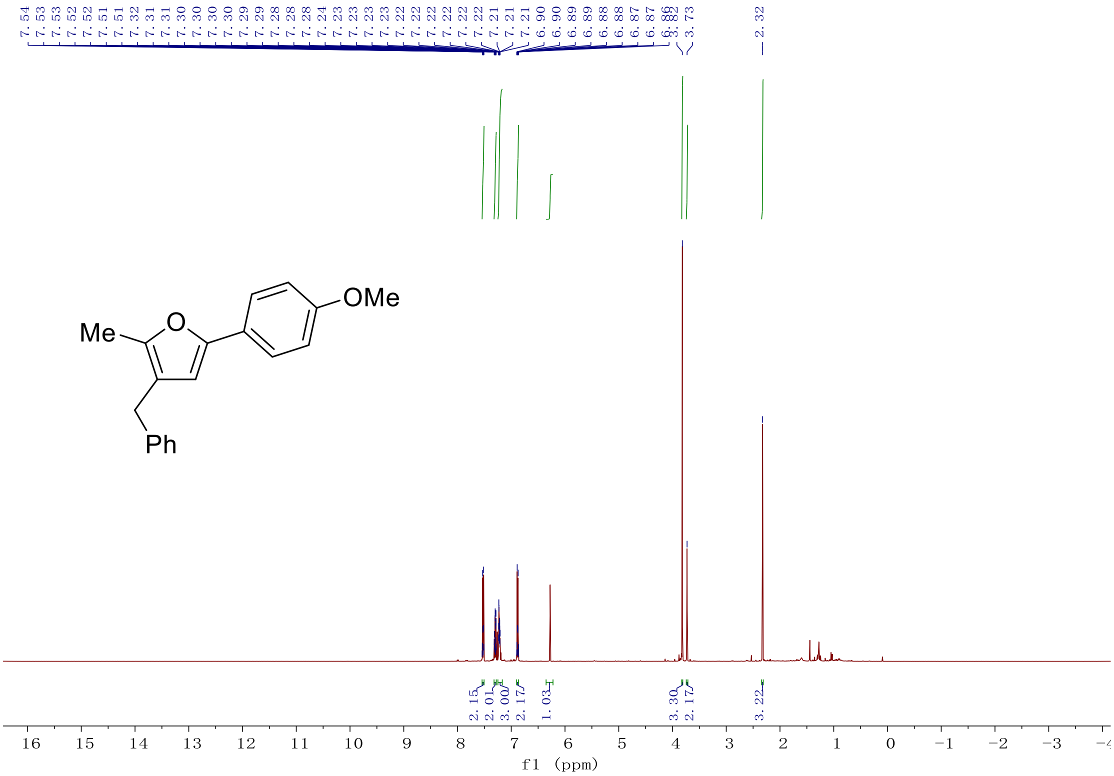

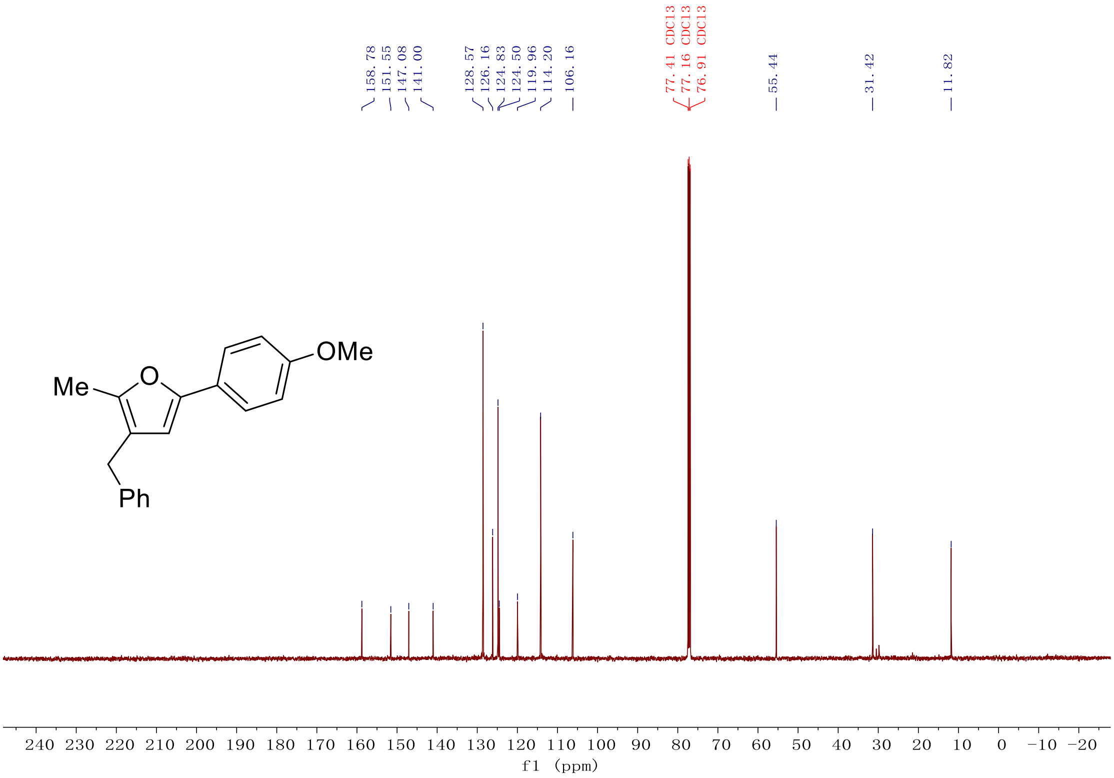

3-(2-Chlorobenzyl)-2-methyl-5-phenylfuran (3p)

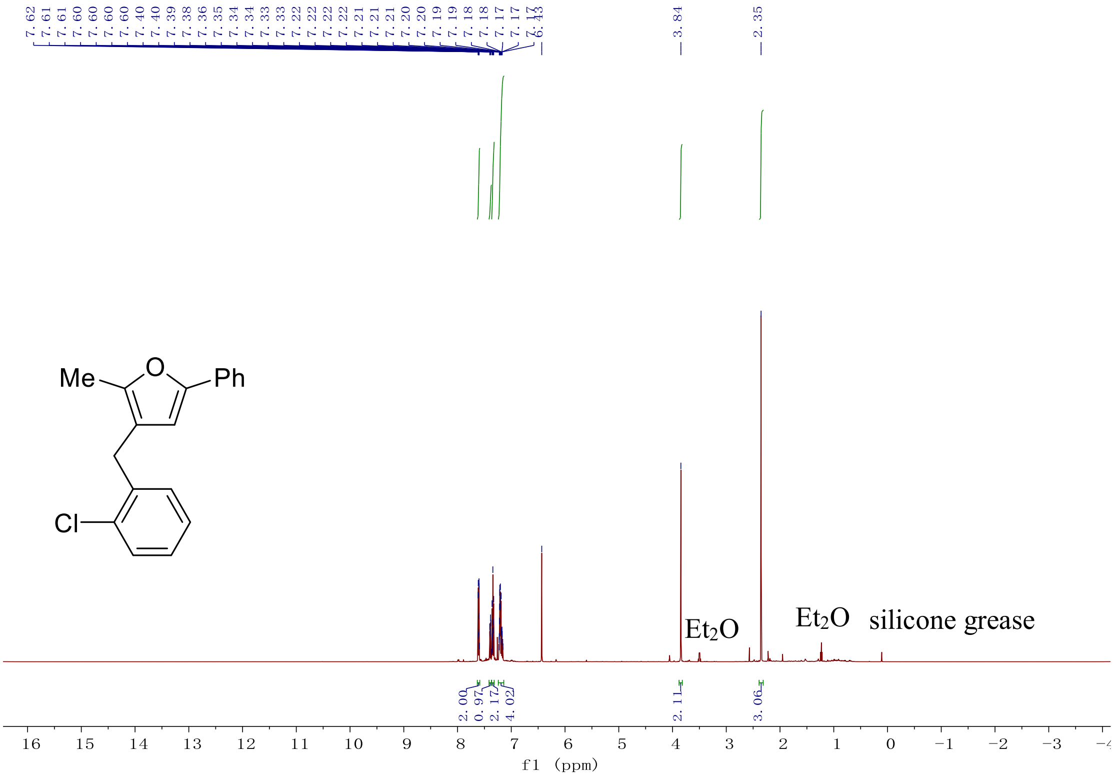

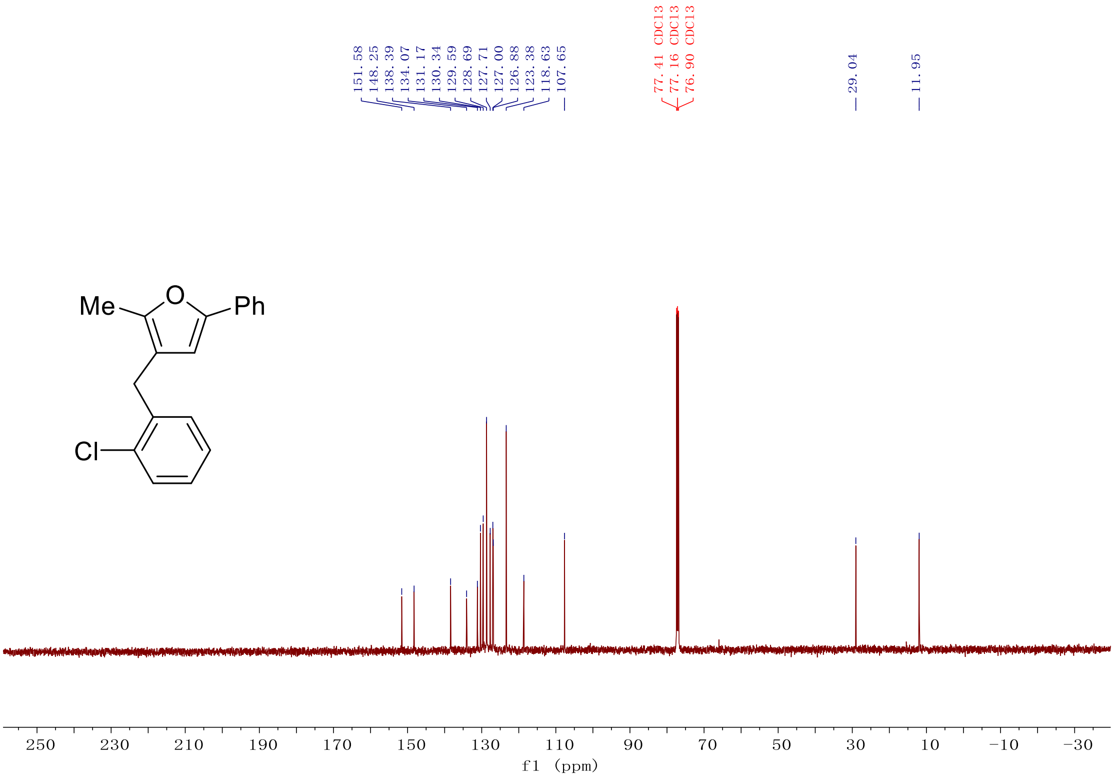

3-(4-Chlorobenzyl)-2-methyl-5-phenylfuran (3q)

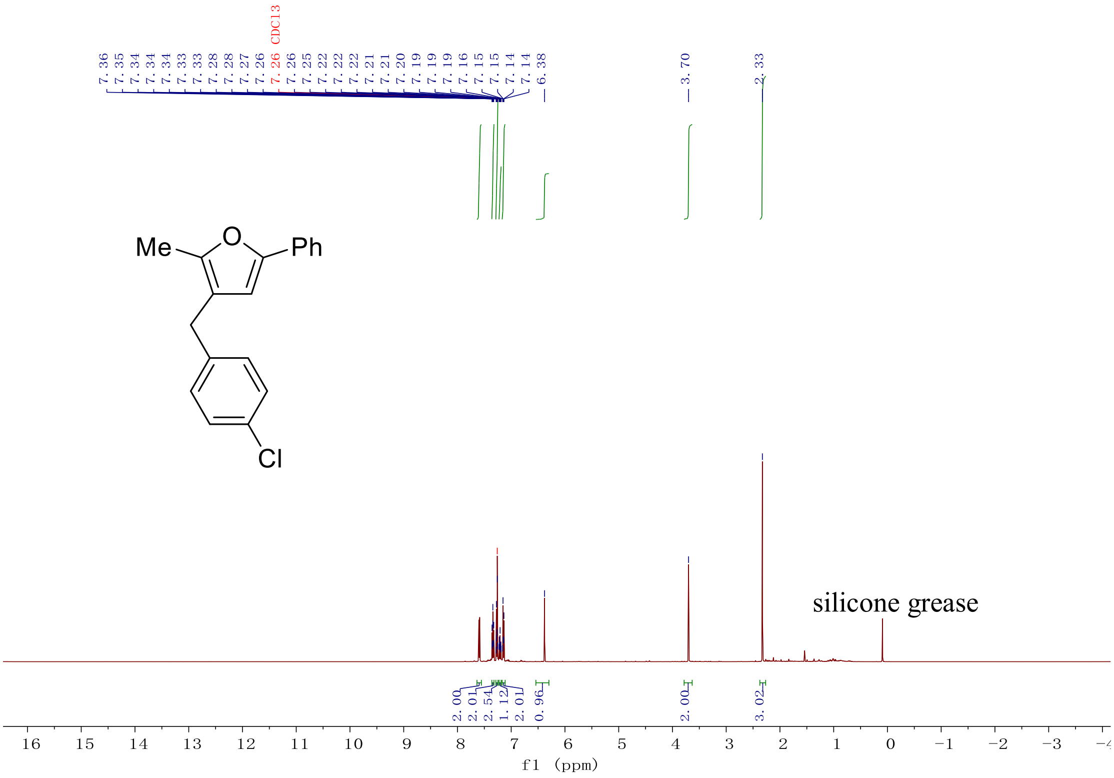

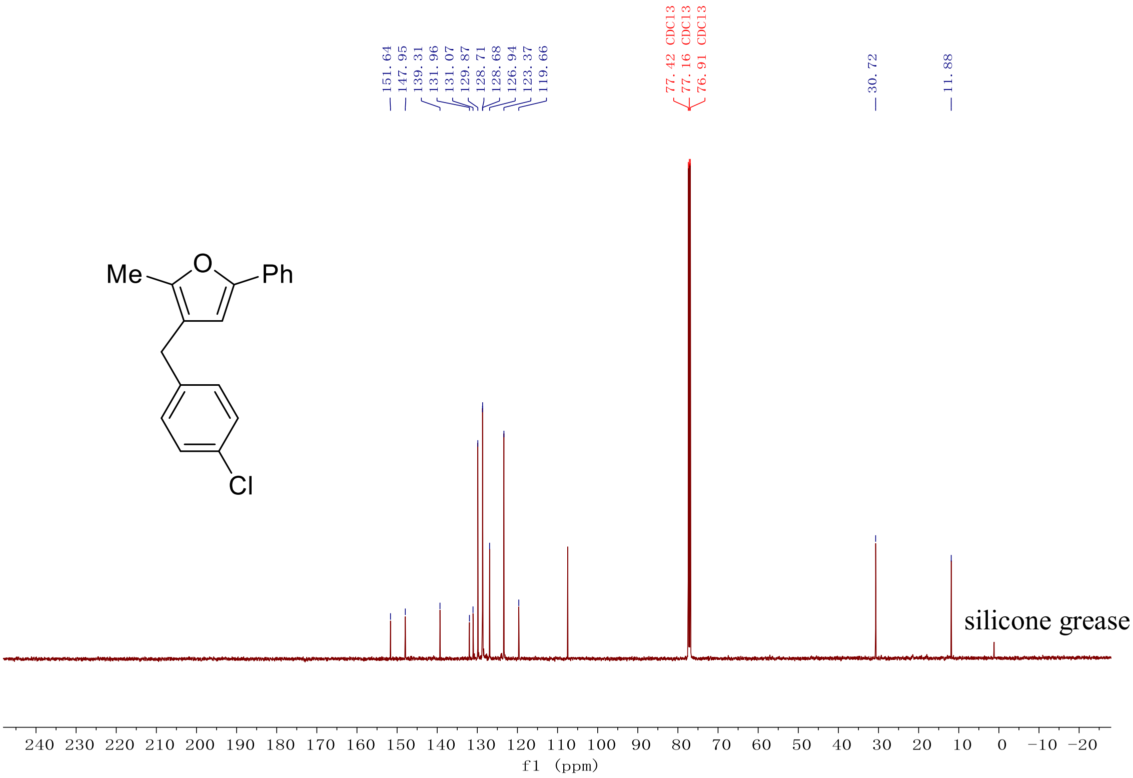

5-((2-Methyl-5-phenylfuran-3-yl)methyl)benzo[*d*][1,3]dioxole (3r)

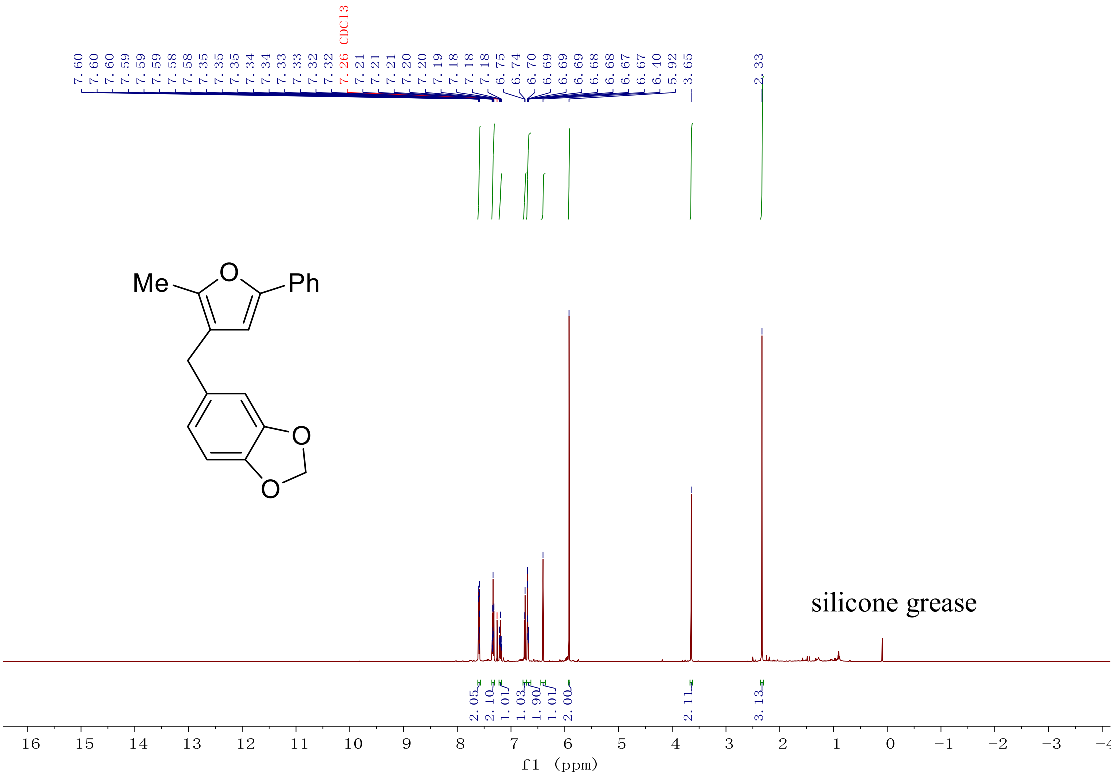

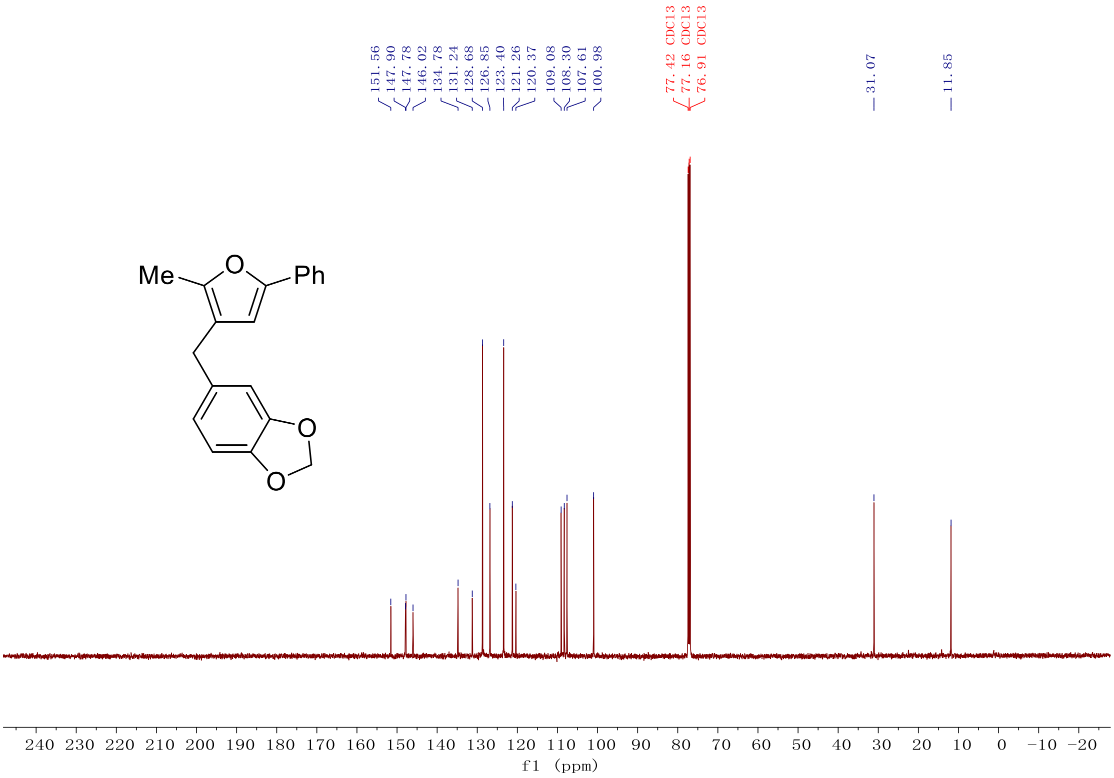

3-Benzyl-5-(4-fluorophenyl)-2-methylfuran (3s)

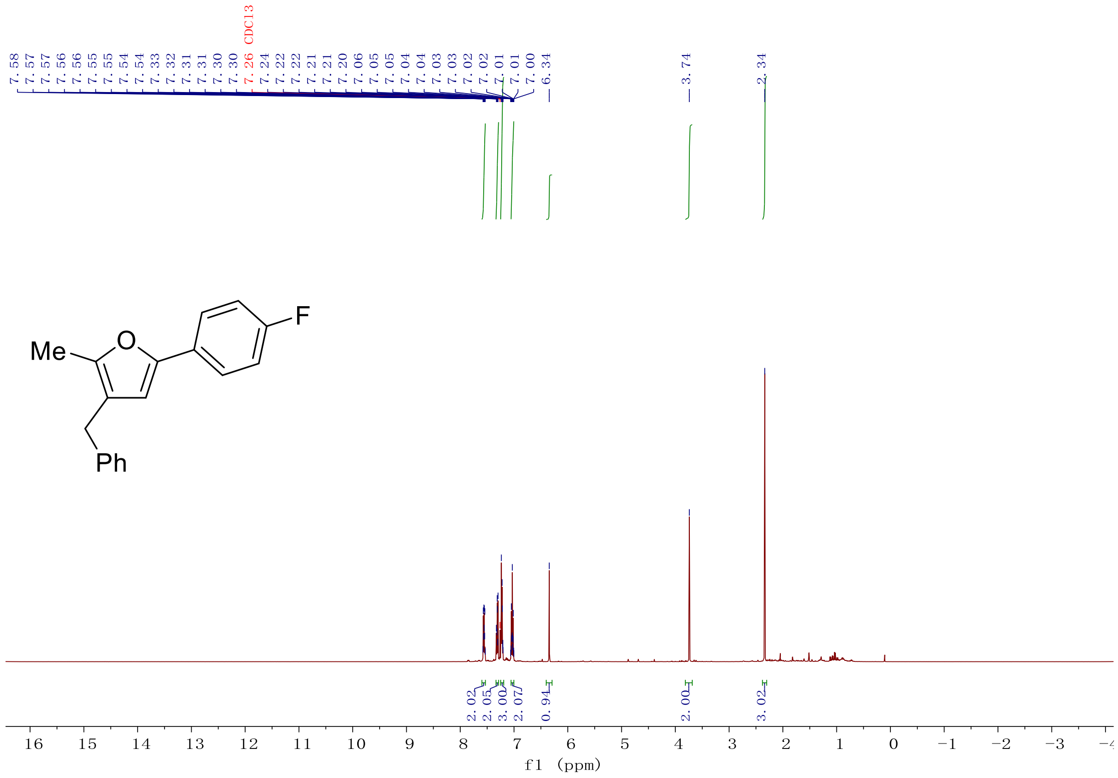

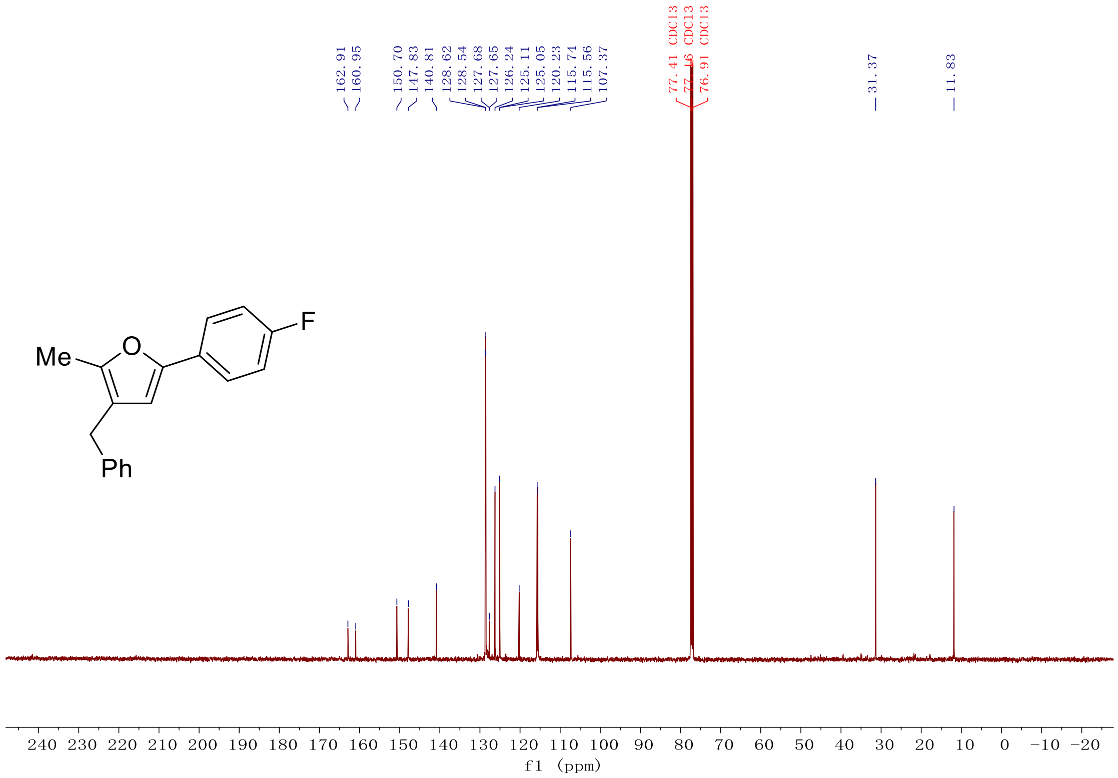

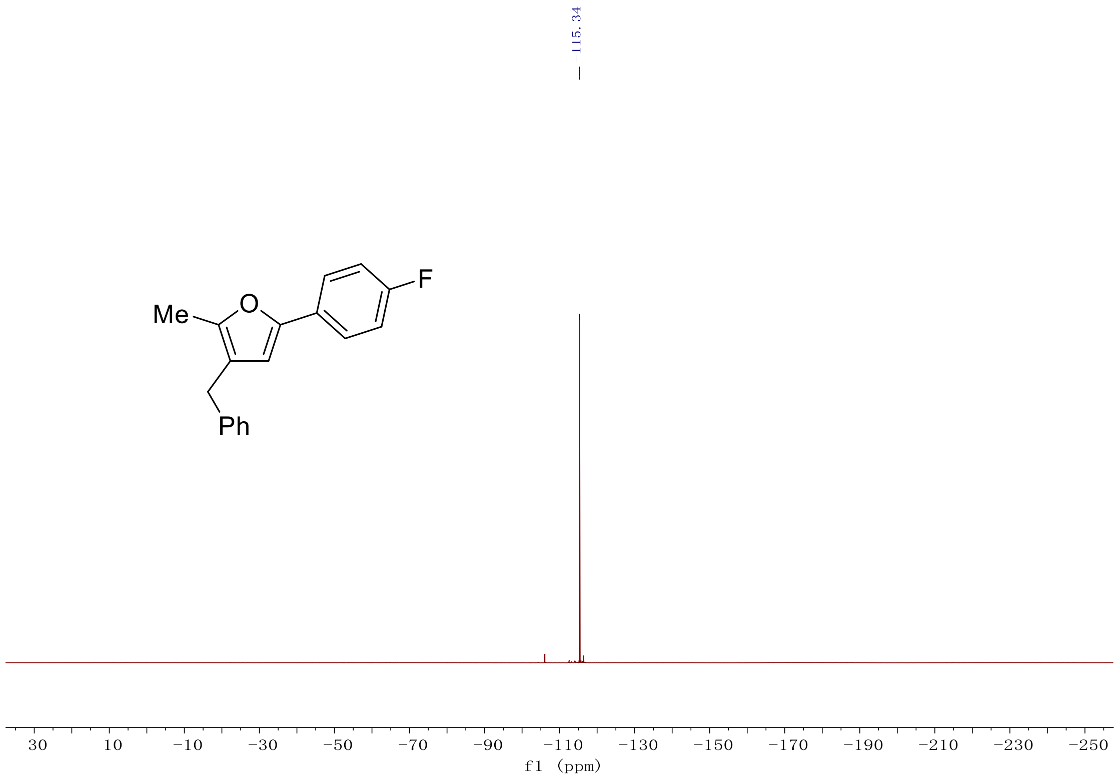

3-Benzyl-5-isopropyl-2-phenylfuran (3t)

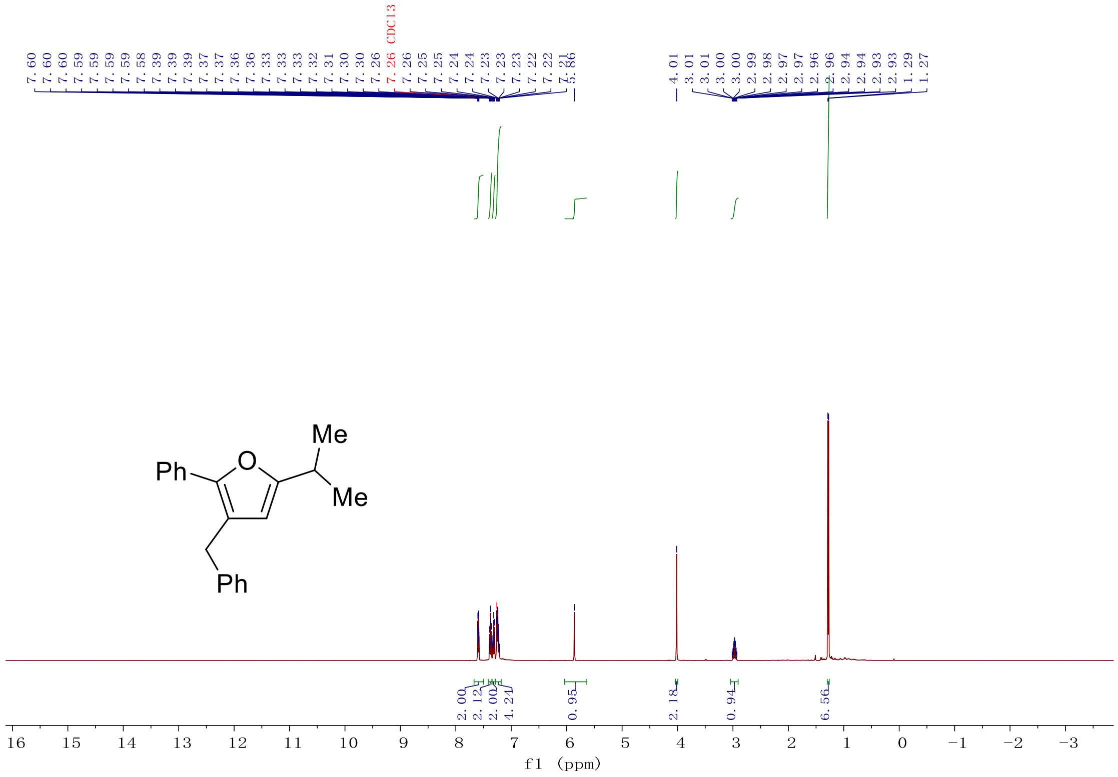

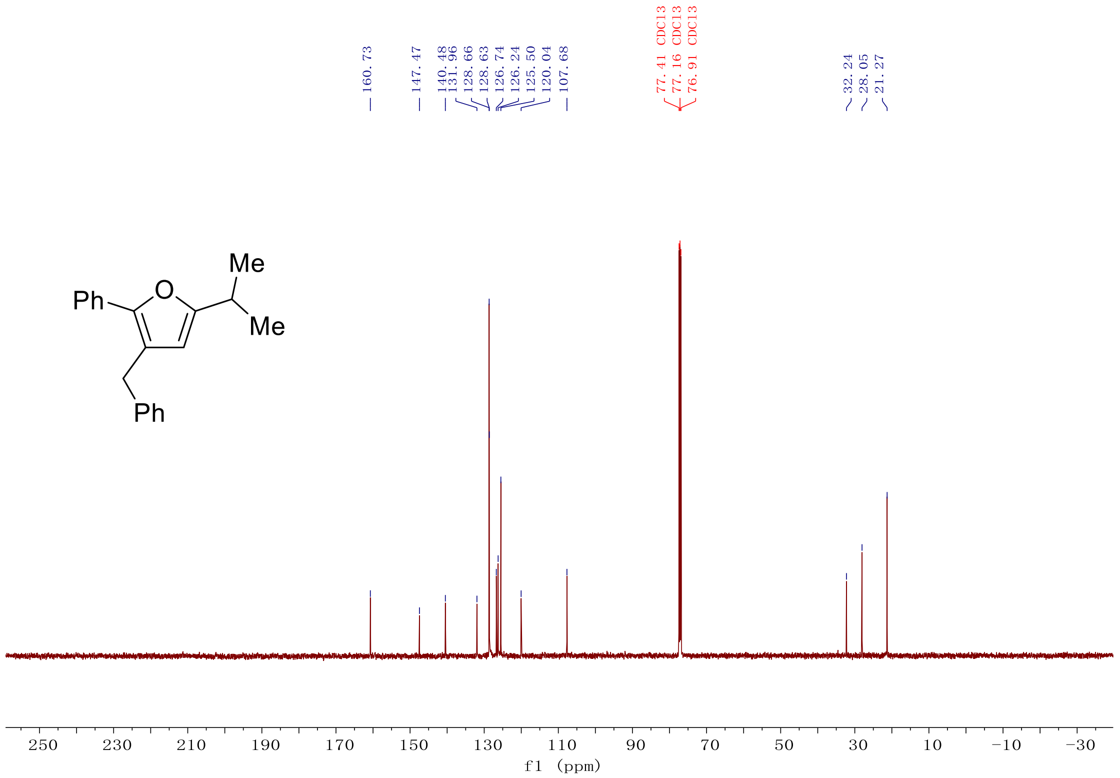

5-Cyclopropyl-2-(4-fluorophenyl)-3-(4-methoxybenzyl)furan (3u)

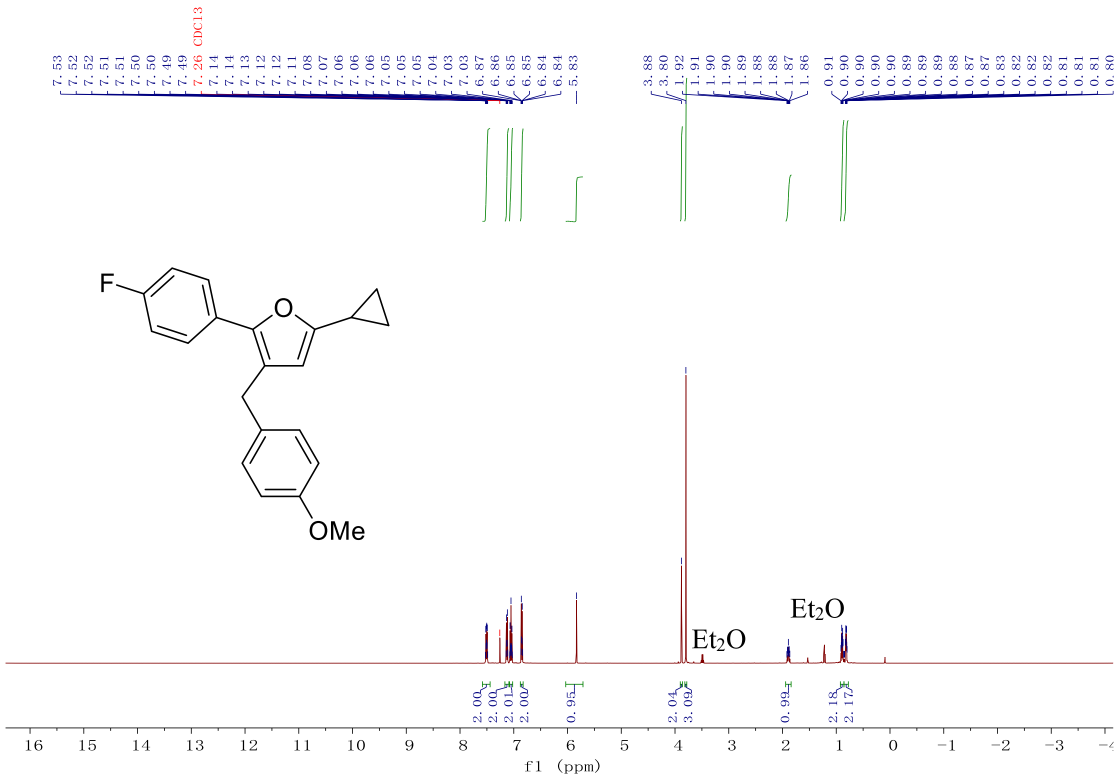

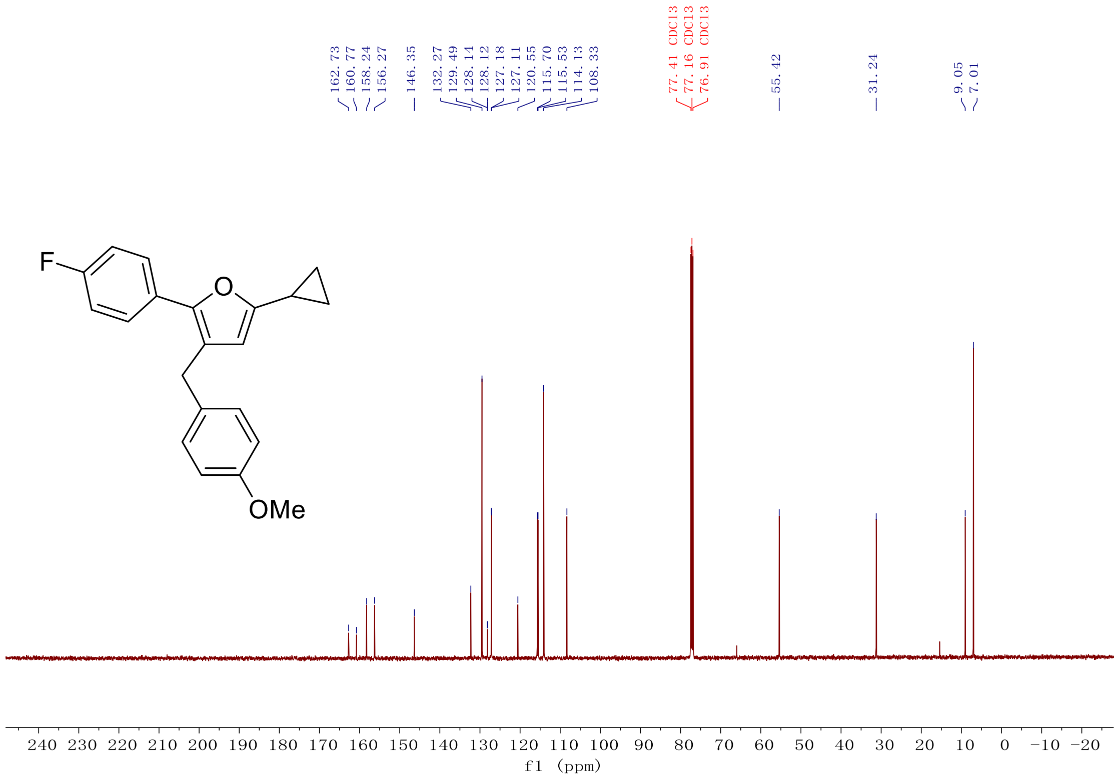
162.73
160.77
158.24
156.27
146.35
132.27
129.49
128.14
128.12
127.18
127.11
120.55
115.70
115.53
114.13
108.33
77.41 CDCl3
77.16 CDCl3
76.91 CDCl3
55.42
31.24
9.05
7.01
F
O
OMe
240 230 220 210 200 190 180 170 160 150 140 130 120 110 100 90 80 70 60 50 40 30 20 10 0 −10 −20
f1 (ppm)

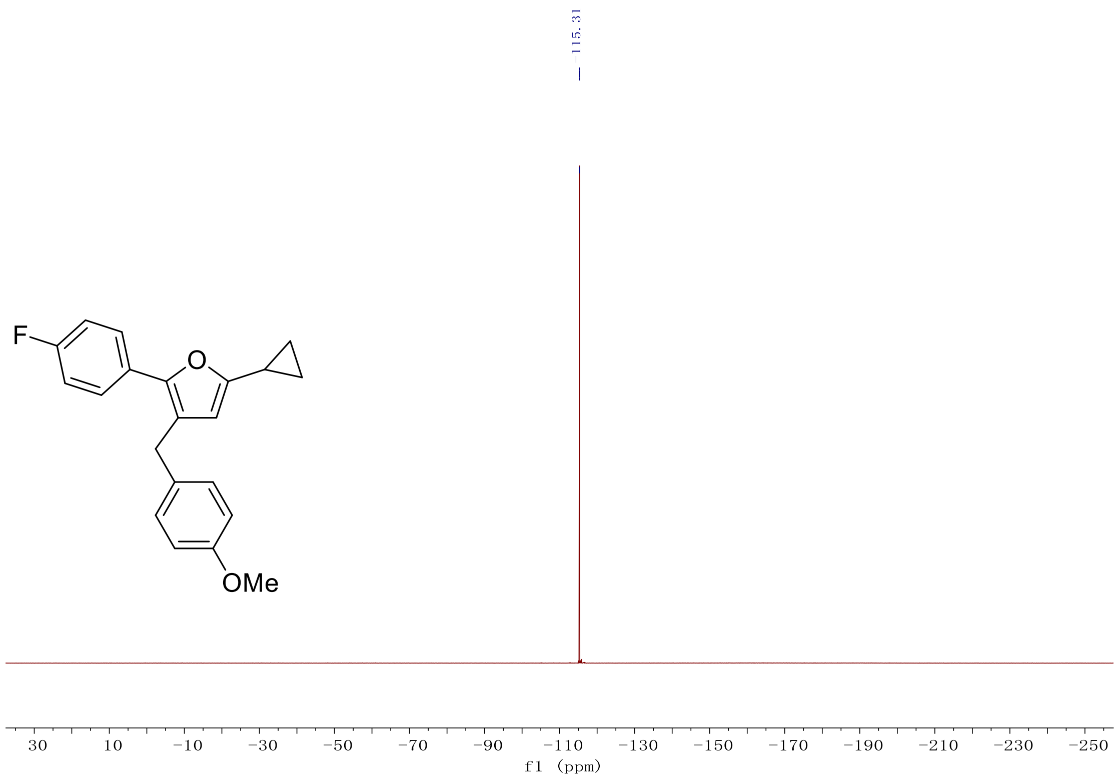
−115.31
F
O
OMe
30 10 −10 −30 −50 −70 −90 −110 −130 −150 −170 −190 −210 −230 −250
f1 (ppm)

5-(3-Chloropropyl)-3-(4-methylbenzyl)-2-phenylfuran (3v)

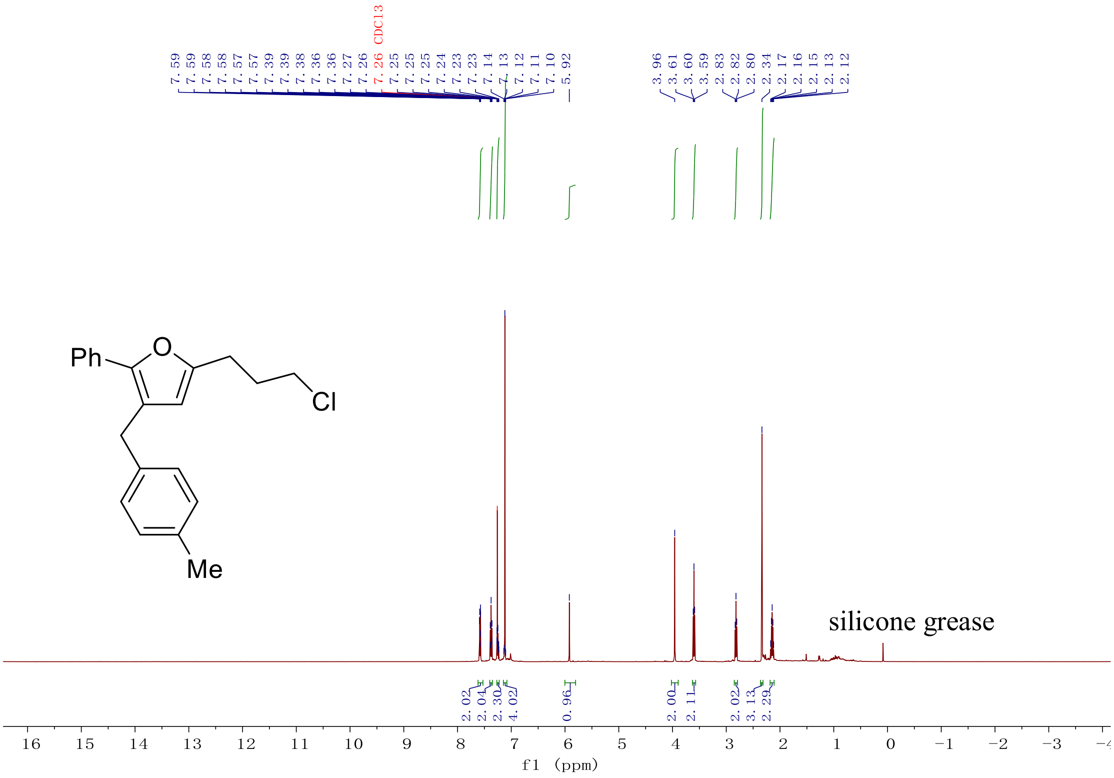

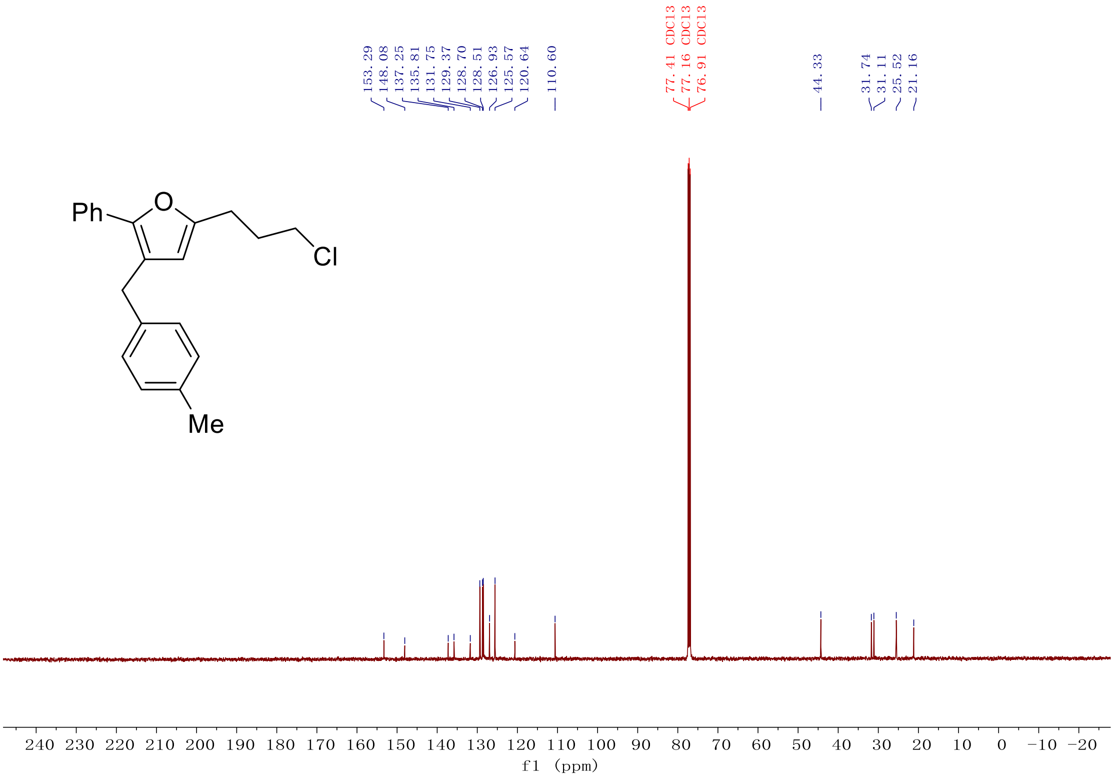

5-Butyl-3-(4-methylbenzyl)-2-phenylfuran (3w)

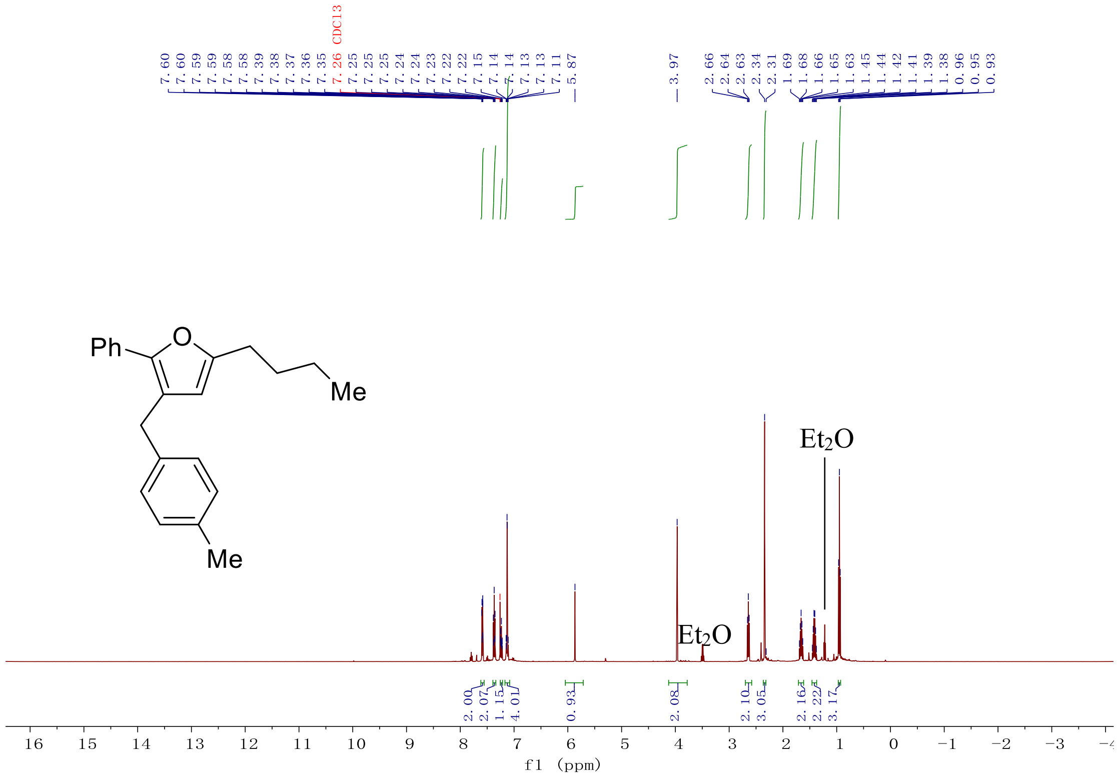

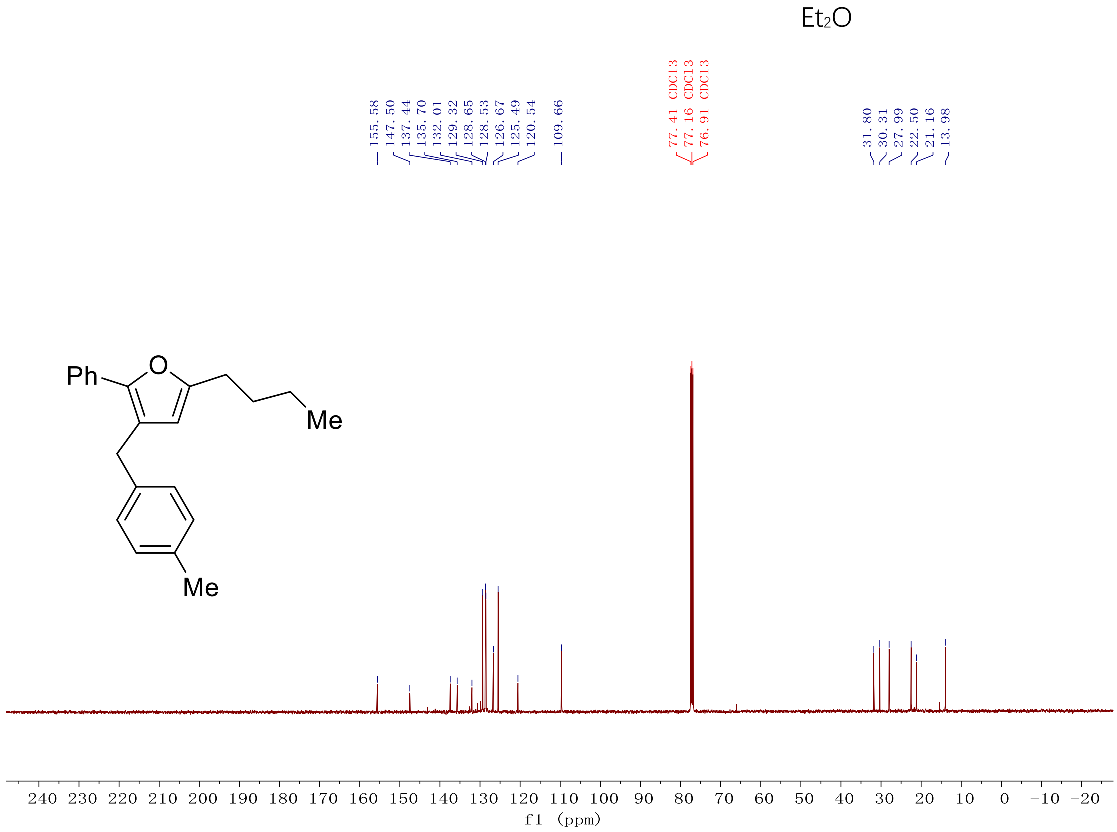

2-Phenyl-4,5,6,7-tetrahydrobenzofuran (3x)

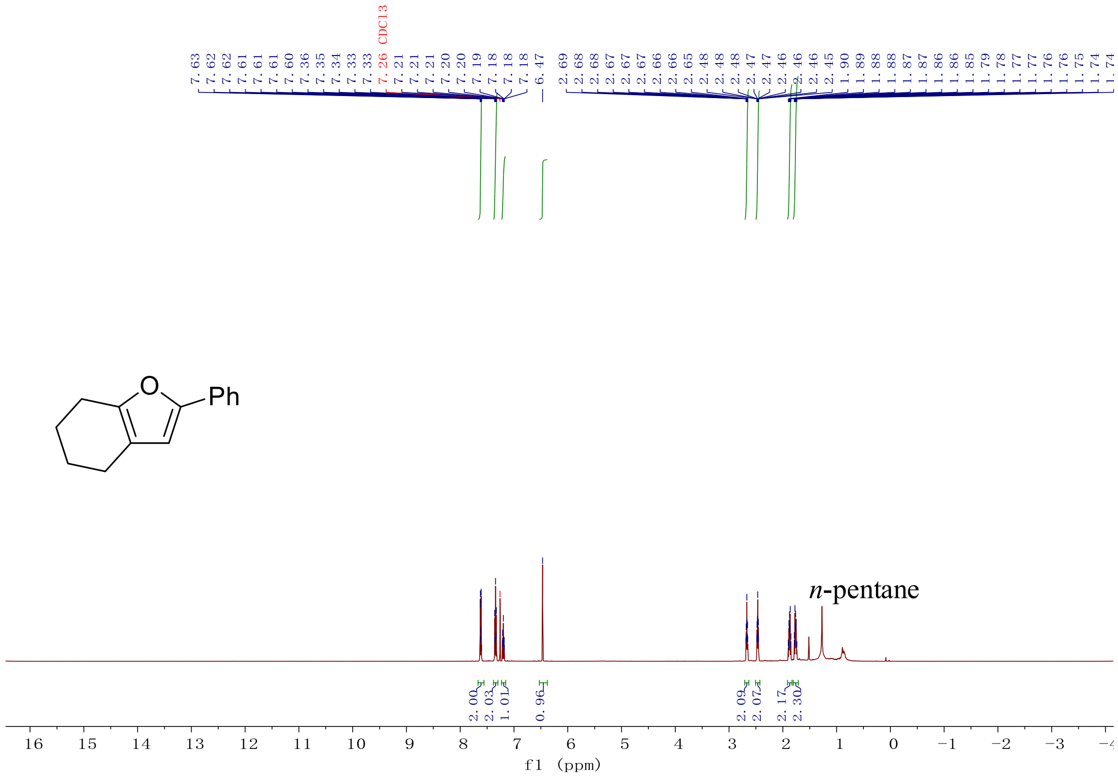

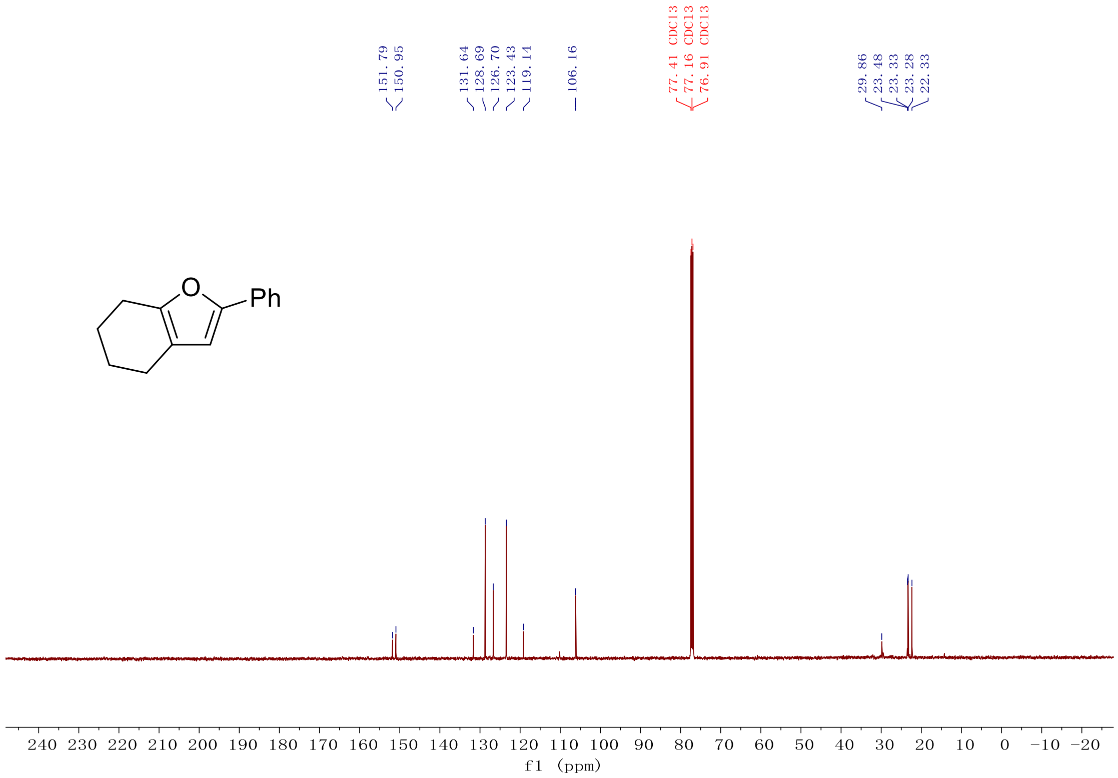

2,5-Diphenyl-3-(*p*-tolylmethyl-*d*)furan (6)

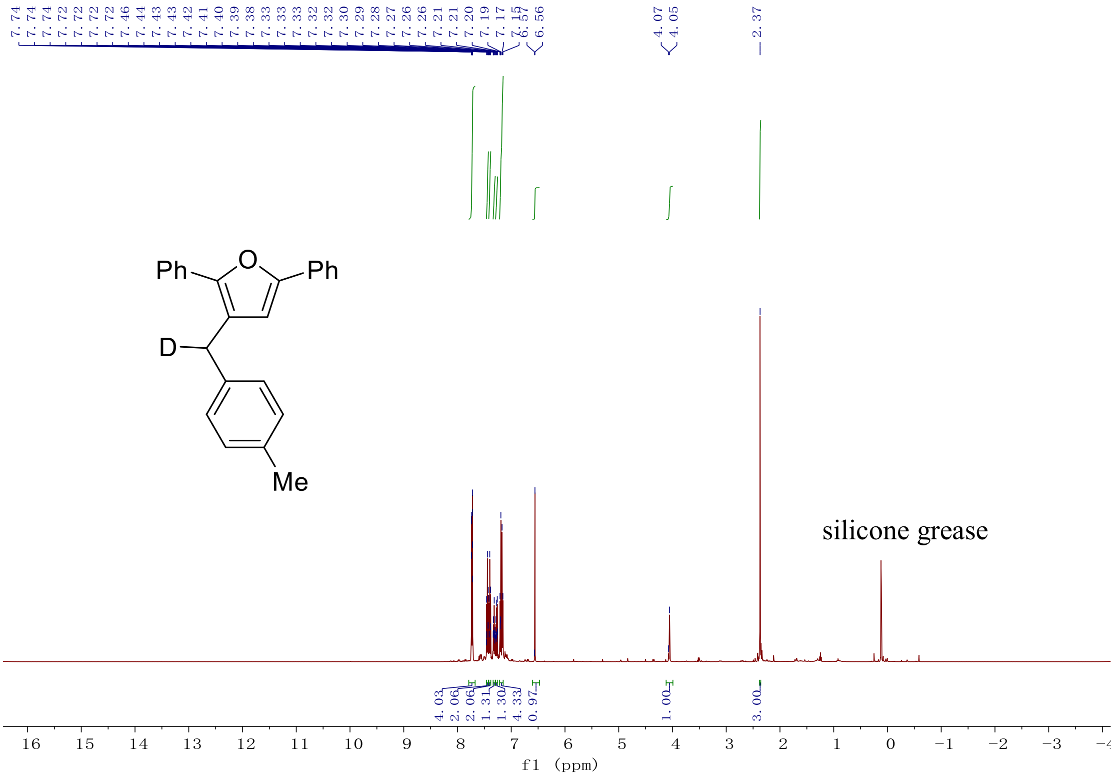

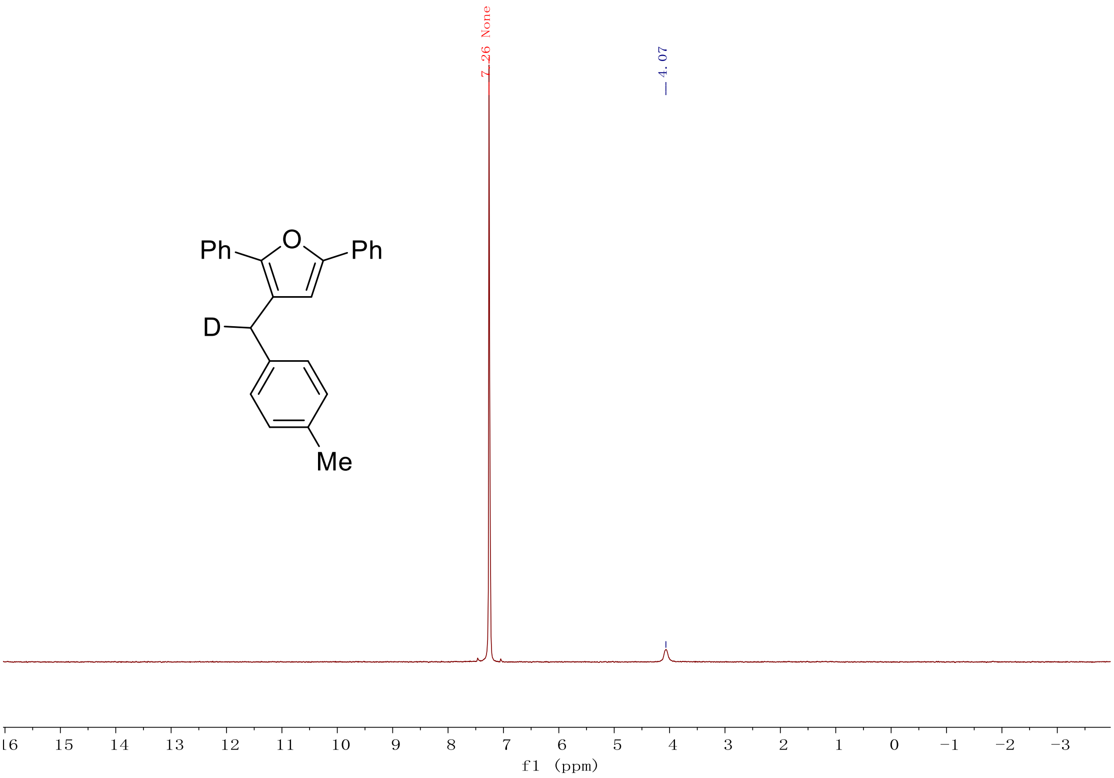

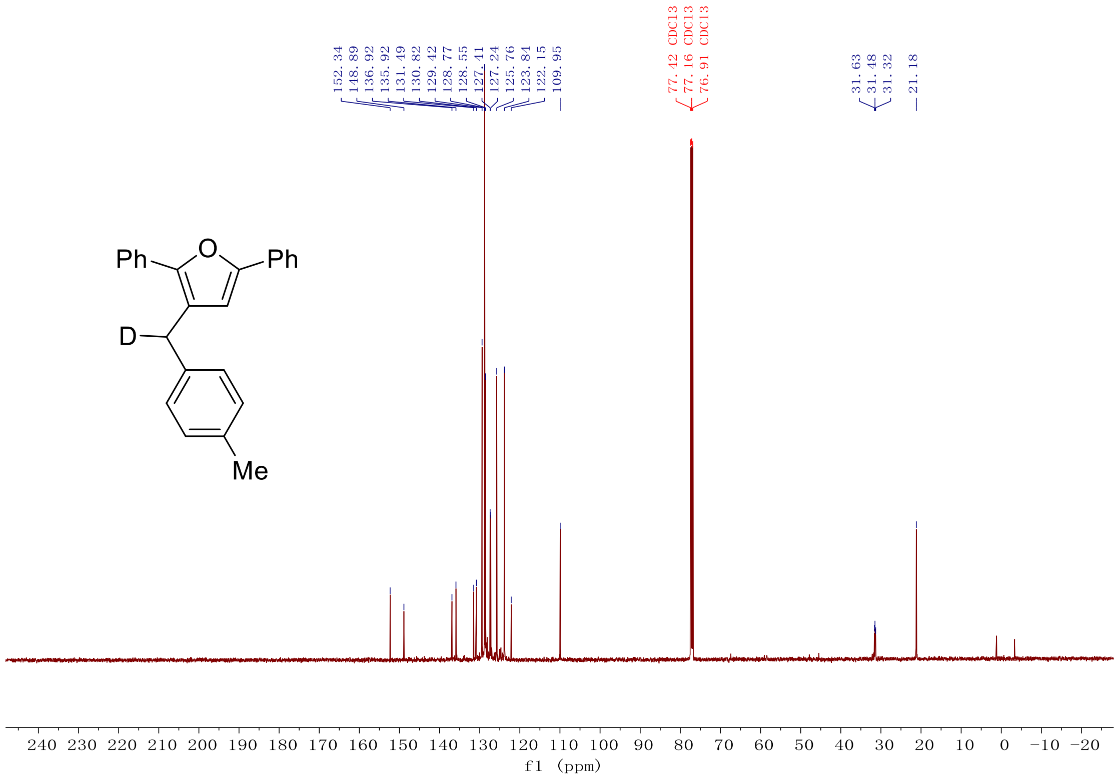

3-(4-Methylbenzyl)-2,5-diphenylfuran-4-*d* (7)

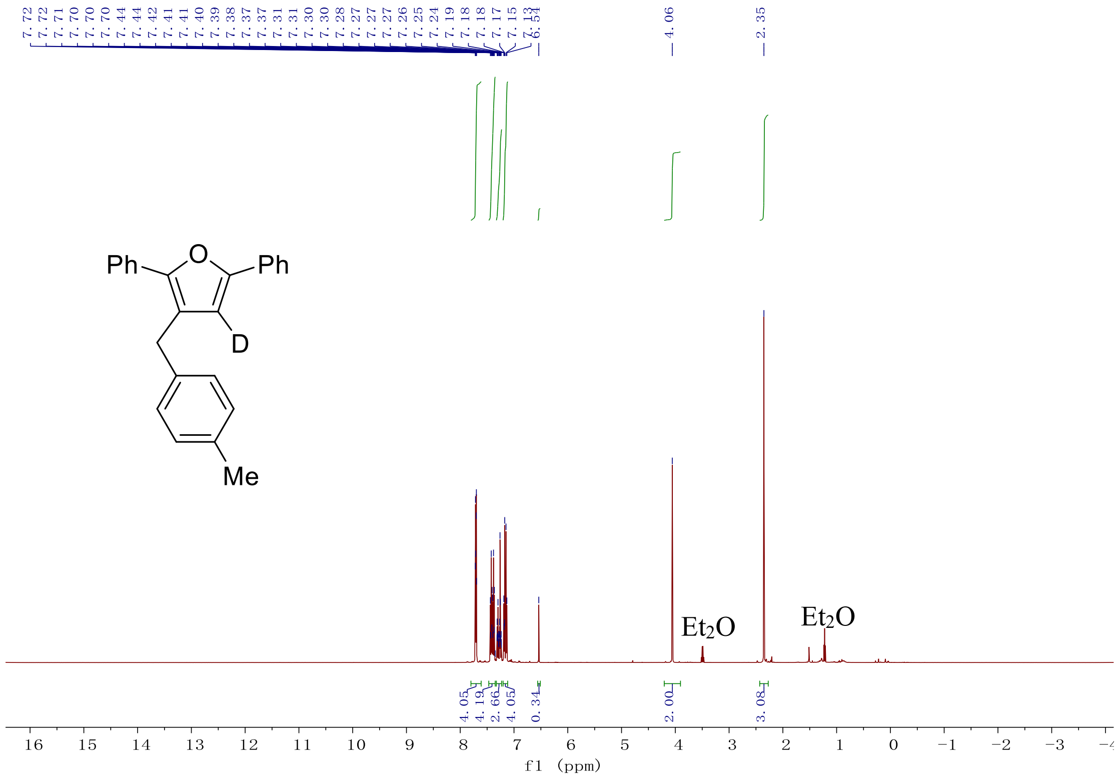

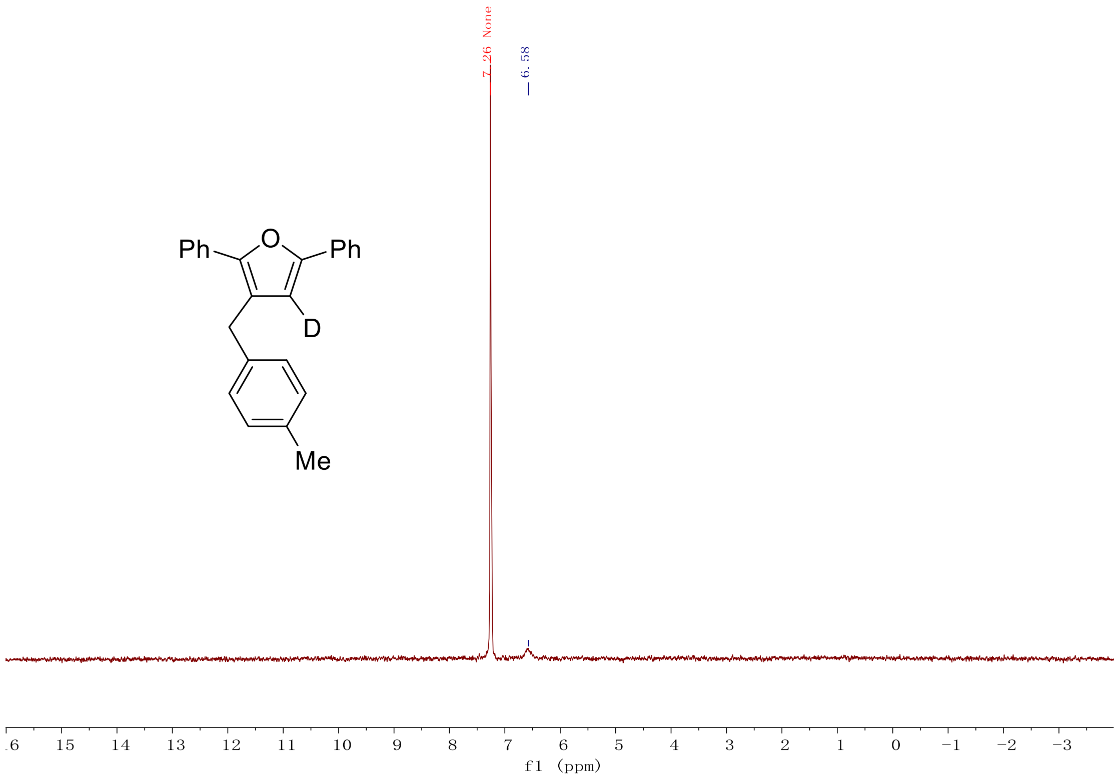
7.26 None
6.58
Ph
O
Ph
D
Me
f1 (ppm)

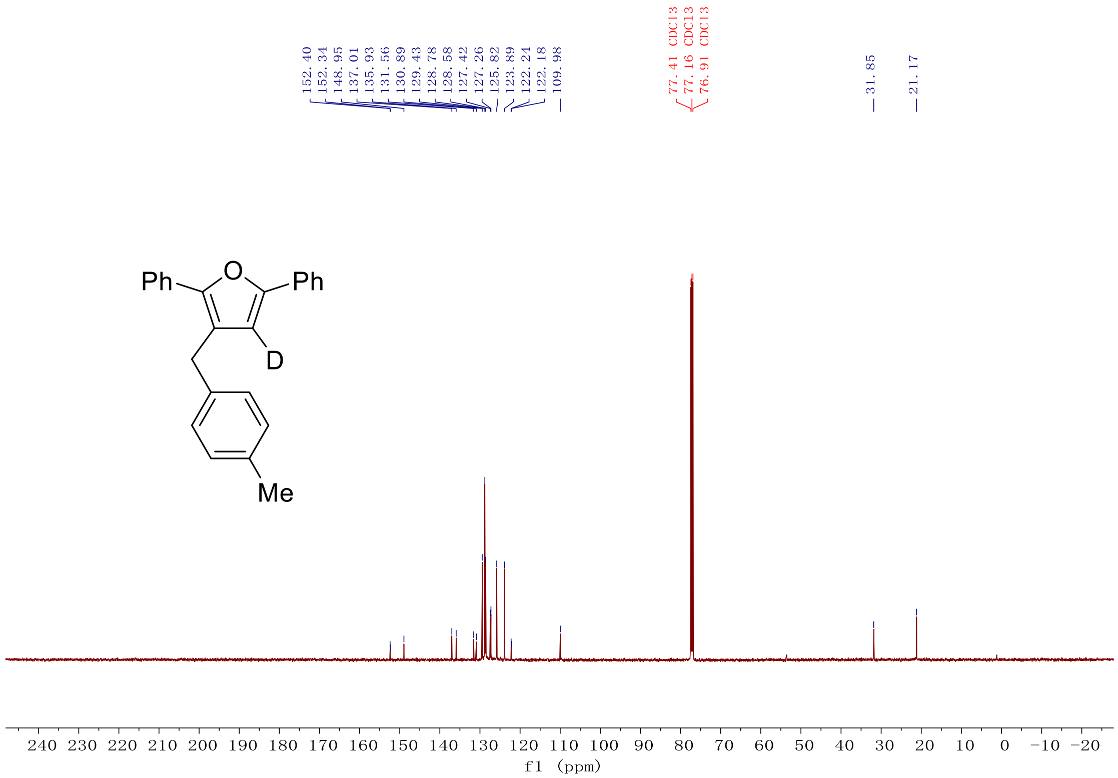
152.40
152.34
148.95
137.01
135.93
131.56
130.89
129.43
128.78
128.58
127.42
127.26
125.82
123.89
122.24
122.18
109.98
77.41 CDCl3
77.16 CDCl3
76.91 CDCl3
31.85
21.17
Ph
O
Ph
D
Me
f1 (ppm)
